# Gonadotropins for pubertal induction in males with hypogonadotropic hypogonadism: systematic review and meta-analysis

**DOI:** 10.1093/ejendo/lvad166

**Published:** 2023-12-21

**Authors:** Emma C Alexander, Duaa Faruqi, Robert Farquhar, Ayesha Unadkat, Kyla Ng Yin, Rebecca Hoskyns, Rachel Varughese, Sasha R Howard

**Affiliations:** Centre for Endocrinology, William Harvey Research Institute, Barts and the London School of Medicine and Dentistry, Queen Mary University of London, London EC1M 6BQ, United Kingdom; Faculty of Life Sciences and Medicine, King’s College London, Guy’s Campus, London SE1 1UL, United Kingdom; Faculty of Life Sciences and Medicine, King’s College London, Guy’s Campus, London SE1 1UL, United Kingdom; Faculty of Life Sciences and Medicine, King’s College London, Guy’s Campus, London SE1 1UL, United Kingdom; Centre for Endocrinology, William Harvey Research Institute, Barts and the London School of Medicine and Dentistry, Queen Mary University of London, London EC1M 6BQ, United Kingdom; Centre for Endocrinology, William Harvey Research Institute, Barts and the London School of Medicine and Dentistry, Queen Mary University of London, London EC1M 6BQ, United Kingdom; Department of Paediatric Endocrinology, Great Ormond Street Hospital NHS Trust, London WC1N 3JH, United Kingdom; Centre for Endocrinology, William Harvey Research Institute, Barts and the London School of Medicine and Dentistry, Queen Mary University of London, London EC1M 6BQ, United Kingdom; Department of Paediatric Endocrinology, Royal London Children’s Hospital, Barts Health NHS Trust, London E1 1BB, United Kingdom

**Keywords:** gonadotropins, hypogonadotropic hypogonadism, spermatogenesis, puberty, Kallmann syndrome

## Abstract

**Objective:**

Hypogonadotropic hypogonadism is characterized by inadequate secretion of pituitary gonadotropins, leading to absent, partial, or arrested puberty. In males, classical treatment with testosterone promotes virilization but not testicular growth or spermatogenesis. To quantify treatment practices and efficacy, we systematically reviewed all studies investigating gonadotropins for the achievement of pubertal outcomes in males with hypogonadotropic hypogonadism.

**Design:**

Systematic review and meta-analysis.

**Methods:**

A systematic review of Medline, Embase, Global Health, and PsycINFO databases in December 2022. Risk of Bias 2.0/Risk Of Bias In Non-randomized Studies of Interventions/National Heart, Lung, and Blood Institute tools for quality appraisal. Protocol registered on PROSPERO (CRD42022381713).

**Results:**

After screening 3925 abstracts, 103 studies were identified including 5328 patients from 21 countries. The average age of participants was <25 years in 45.6% (*n* = 47) of studies. Studies utilized human chorionic gonadotropin (hCG) (*n* = 93, 90.3% of studies), human menopausal gonadotropin (*n* = 42, 40.8%), follicle-stimulating hormone (FSH) (*n* = 37, 35.9%), and gonadotropin-releasing hormone (28.2% *n* = 29). The median reported duration of treatment/follow-up was 18 months (interquartile range 10.5-24 months). Gonadotropins induced significant increases in testicular volume, penile size, and testosterone in over 98% of analyses. Spermatogenesis rates were higher with hCG + FSH (86%, 95% confidence interval [CI] 82%-91%) as compared with hCG alone (40%, 95% CI 25%-56%). However, study heterogeneity and treatment variability were high.

**Conclusions:**

This systematic review provides convincing evidence of the efficacy of gonadotropins for pubertal induction. However, there remains substantial heterogeneity in treatment choice, dose, duration, and outcomes assessed. Formal guidelines and randomized studies are needed.

**Significance**

Hypogonadotropic hypogonadism presents in around 1 in 10 000 live births and is a key cause of absent, partial, or arrested puberty. It has substantial consequences: infertility, reduced sexual function, poor bone health, psychological distress, and reduced quality of life. In this systematic review, we describe current practice and pubertal outcomes with the treatment of adolescent and young adult male patients with gonadotropins. We find that gonadotropins induce significant increases in testicular volume, penile size, and testosterone in over 98% of analyses. Spermatogenesis is achieved for the majority of patients, particularly in those receiving combined follicle-stimulating hormone and human chorionic gonadotropin. These findings emphasize the importance of treatment with gonadotropins for pubertal induction or completion and provide the impetus for definitive guidelines and randomized controlled trials.

## Introduction

Hypogonadotropic hypogonadism (HH) often presents with absent, partial, or arrested pubertal development in adolescence.^1^ Hypogonadotropic hypogonadism can be congenital (CHH) or acquired. In males, CHH affects between 1 in 4400 and 15 000 live births.^2^ The condition may be secondary to a lack of gonadotropin-releasing hormone (GnRH) secretion from the hypothalamus or abnormal gonadotropin (luteinizing hormone [LH] and follicle-stimulating hormone [FSH]) secretion from the anterior pituitary gland, leading to reduced endogenous sex steroid production from the gonads. Congenital HH may be idiopathic (IHH) or, when associated with hypo- or anosmia, it is termed Kallmann syndrome.^3^ Acquired HH occurs due to direct damage to the hypothalamus or pituitary (eg, due to surgery, radiotherapy, and trauma) leading to impaired secretion of gonadotropins.^4^

In males, the hypothalamic–pituitary–gonadal axis is normally activated from birth to 6 months of age, manifesting as “mini-puberty,” leading to a rise in gonadotropins (LH and FSH) and sex steroids and resultant penile and testicular growth.^5,6^ In healthy males, FSH leads to the proliferation of Sertoli cells in the testes and, hence, testicular growth, facilitating post-pubertal spermatogenesis. During the mini-puberty period, Sertoli cells do not express androgen receptors, so spermatogenesis is not stimulated in infancy.^7,8^ The long-term significance of the mini-puberty period is shown in animal and human studies; in rhesus monkeys, suppression of mini-puberty with a GnRH agonist leads to lower post-pubertal testicular volumes and sperm counts.^9^ A prospective cohort study of boys born to healthy mothers has also shown that serum testosterone in infancy correlates with adult total sperm counts.^10^ In male patients with HH, the absence of gonadotropin stimulation during mini-puberty and then adolescent puberty will therefore impede testicular maturation with resultant small testicular volumes and is frequently associated with micropenis and/or undescended testes (cryptorchidism).

The role of LH is to stimulate Leydig cells to produce testosterone, and hence, low or absent concentrations of testosterone are seen in males with HH. Inhibin B and AMH, peptides secreted by Sertoli cells, are important biomarkers in the diagnosis of HH and for monitoring therapeutic response to gonadotropins for the induction of puberty and spermatogenesis (although their use can be limited by the lack of universally accepted threshold values).^11^ The wider sequelae of congenital or acquired HH in later life include low testicular volumes, reduced sexual function, poor bone health, psychological distress, reduced quality of life, and ultimately infertility.^12-15^

These consequences provide a clinical rationale for induction of puberty in male patients with CHH, traditionally with testosterone from the age of 12 years.^14,15^ However, whilst exogenous testosterone can increase serum testosterone and induce virilization, it will not promote testis development nor induce spermatogenesis in adolescent and young adult men with this condition.^16^ Potential for fertility is a key goal for patients presenting to the clinic and requesting treatment. Consequently, there has been increasing interest in the use of gonadotropins for pubertal induction, which not only will elevate testosterone but will also induce testicular growth and spermatogenesis.^16^ The Endo-ERN guideline on pubertal induction in patients with congenital gonadotropin deficiency was published in 2022, but based on the available evidence, no firm conclusion was drawn regarding the optimal therapy to induce or sustain puberty in CHH.^14^ Two prior systematic reviews have focused primarily on spermatogenesis, with less of a focus on pubertal outcomes.^17,18^ This systematic review therefore aims to review the published evidence regarding the efficacy of gonadotropin therapy for the attainment of pubertal outcomes in males with HH.

## Methods

### Registration and search strategy

This review was registered on PROSPERO (CRD42022381713). The conduct of this review complied with the Declaration of Helsinki. As no human subjects were involved in the review, no independent ethical committee review was conducted.

We searched MEDLINE, Embase, Global Health, and PsycINFO, from inception until December 2022 and scanned reference lists of shortlisted studies. Our search terms combined (hypogonadotrophic hypogonadism [and derivatives] AND males [and derivatives] AND gonadotropins [and derivatives] AND treatment [and derivatives] AND puberty/outcomes [and derivatives]) (see Box 1).

### Eligibility criteria

The population was males with HH (including congenital, idiopathic, or mixed populations). Eligible studies administered gonadotropin therapy, including FSH (urinary or recombinant), human chorionic gonadotropin (hCG), LH, human menopausal gonadotropin (hMG) (combination therapy of LH and FSH^19^), or GnRH. Comparators were any or none. The assessed primary outcome was efficacy encompassing testicular volume, penile length, sperm count, and spermatogenesis rate and biochemical outcomes including testosterone and inhibin B. Secondary outcome was safety (adverse effects). All study designs were eligible for inclusion.

Exclusion criteria are follows: no gonadotropins administered; no pubertal outcomes included; no primary results; intervention of <6 months; animal study; full text not available; fewer than 10 males with HH included; fewer than five patients treated with gonadotropins; patient groups with mixed aetiologies of hypogonadism (eg, Prader Willi); acquired HH only; conference abstracts; not published in English/no translation available; published prior to 1990; outcomes not separated by the patient group; outcomes not quantified; female subjects; and duplicates.

### Data extraction and risk of bias assessment

Retrieved titles and abstracts, and shortlisted full texts, were screened by two independent researchers on the Covidence platform (www.covidence.org). Any discrepancies were resolved by discussion. Eligible studies underwent data extraction using a pre-piloted extraction form. Each data extraction was performed by one researcher and checked by a second.

Quality appraisal was performed by two independent researchers with discrepancies resolved by discussion or by the senior author. We used the Cochrane Risk of Bias for Non-Randomized Studies of Interventions (ROBINS-I) tool for non-randomized studies, the Cochrane Risk of Bias 2.0 tool for randomized controlled trials (RCTs), and National Heart, Lung, and Blood Institute (NHLBI) Quality Assessment Tools for single arm pre–post, cohort, and case-control studies.^20-22^ Studies returning critical/high risk of bias were excluded from data synthesis, as well as studies scoring below 7 after NHLBI scoring. Studies thought to contain duplicate data were considered singly or with prioritization of the larger cohort for data synthesis.

### Data synthesis and meta-analyses

Meta-analyses of pre–post values for testicular volume, penile length, and biochemical parameters were not performed due to known risk of bias with pre–post change studies.^23^ Data syntheses for these outcomes were performed, identifying the proportion of studies performing statistical analyses that reported a significant change in these outcomes and visualizing these with modified Albatross plots of sample sizes and *P*-values.^24^ Meta-analyses of other outcomes were undertaken using R Studio version 2022.12.0+353. For reporting of spermatogenesis, some studies used semen analysis post-ejaculation, and others used rates after surgical extraction of sperm. Rate from ejaculation was used for consistency when both were reported. A proportional meta-analysis for spermatogenesis rates and adverse effect rates was conducted, using the random effects model and produced a point estimate with 95% confidence interval (CI).^25^ Heterogeneity was assessed using Cochran's *Q* and inconsistency using *I*^2^. In view of high levels of heterogeneity in study design and treatment schedules, the random effect model was used for all analyses.

## Results

**Figure 1.**
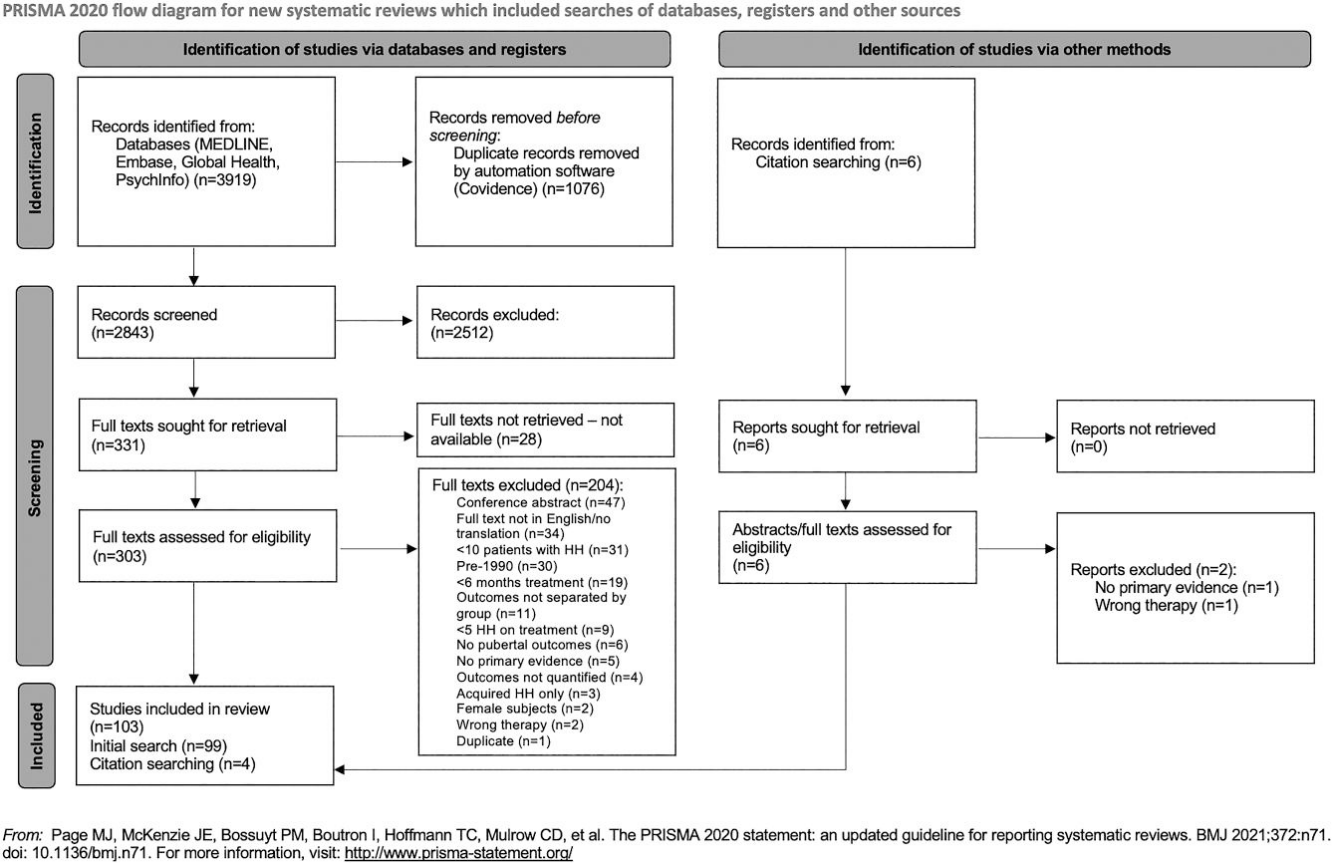
PRISMA flow diagram for study screening and selection.

The Preferred Reporting Items for Systematic Reviews and Meta-Analyses (PRISMA) flow diagram illustrating the study screening process is shown in Figure 1.^26^ Of 3925 retrieved abstracts, 103 studies met inclusion criteria (76 pre–post observational studies, 19 comparative non-randomized studies, six RCTs, and two other). These described 5328 patients across 21 countries. A summary of all included studies is in Tables S1 and S2.

The distribution of average ages (median or mean) of participants across all studies was from 13 to 43 years. The median of the average age was 25.3 years (interquartile range [IQR] 21.4-30.6 years) and was <25 years in 45.6% (*n* = 47) of studies. Eight studies investigated cohorts in which the average age was under age 18 years.^16,27-33^

### Summary of treatment, cohorts, and pubertal status across all studies

Across all 103 studies, hCG was assessed in 90.3% (*n* = 93), followed by hMG in 40.8% (*n* = 42) of studies, FSH in 35.9% (*n* = 37), and GnRH in 28.2% (*n* = 29). Testosterone was used as a comparative or combination therapy, or as pre-treatment, for 26.2% (*n* = 27) of studies. Treatment doses, frequency, and duration were highly variable across all studies. The average weekly dosage of hCG ranged from 625 to 15 000 IU (median 4500, IQR 3750-6000). The average weekly dose of FSH ranged from 150 to 787.5 IU (median 412.5, IQR 225-450). The median dose for pulsatile GnRH dosing was 10 µg every 90 min (range from 2 µg every 90 min to 20 µg every 90 min with further dose titration if required). The median duration of the evaluated treatment course was 18 months (IQR 10.5-24).

All cohorts included some subjects with IHH or CHH, as per inclusion criteria. Fifty studies (48.5%) had cohorts exclusively with IHH or CHH, with no patients with acquired/pituitary causes (Table S1), and the remainder were mixed cohorts. In total, 1266 patients (23.8% of 5328 total) were explicitly described as having Kallmann syndrome.

Pubertal status of the cohorts was quantified in 67 studies, and of those where results were available a median of 80% (IQR 59%-100%) of participants were pre-pubertal or had a lack of spontaneous puberty (Tables S1 and S2). Eight other studies described genital Tanner stages or average testicular volumes where the average was <Tanner Stage 2 or average testicular volume of <4 mL. Pubertal status was affected by intrinsic disease severity and variability in pre-treatment (with androgens and with gonadotropins) across studies.

### Quality appraisal

The median NIHLBI score for observational studies was 9/12 (IQR 8-10) (Tables S1 and S2). Studies scored well for clearly stating the study question or objective (100%) and selecting participants who were eligible or representative (100%) but infrequently had an adequately large sample size, with the threshold set at 30 participants (44.7%). Overall ratings showed that, of six RCTs, one study had a high risk of bias, four studies had some concerns, and one study had a low risk of bias. Of non-randomized studies of interventions (comparative) studies, nine studies had a moderate risk of bias, nine had a serious risk of bias, and one study had a critical risk of bias. Overall, 44.0% of non-randomized or randomized comparative studies appraised using RoB 2.0 or ROBINS-I had serious (randomized) or high (non-randomized) risk of bias in at least one domain (Table S3). Studies scored well for low risk of bias in measurement of outcomes (100%) due to the use of objective measurements as primary outcomes and in low risk of bias due to deviations from intended measurements (84%). Five studies were excluded from outcome synthesis due to low scoring or insufficient outcome reporting. Additionally, 14 pairs or groups of studies were identified as potentially containing the same or similar cohorts, and duplicate data were excluded from data synthesis where identified.

### Clinical outcomes in all cohorts

#### Testicular volume

We identified 52 statistical analyses of change in testicular volume after administration of gonadotropins across 37 papers. Of these, 98.1% (*n* = 51) found that administration of gonadotropins caused a significant increase in size of the testicles. Treatment options included 30.8% (*n* = 16) for hCG + FSH, 21.2% (*n* = 11) hCG + hMG, 19.2% (*n* = 10) hCG alone, 17.3% (*n* = 9) GnRH, and 11.5% (*n* = 6) other combinations/various. Figure S1 illustrates a plot of *P*-values against sample sizes.

**Figure 2.**
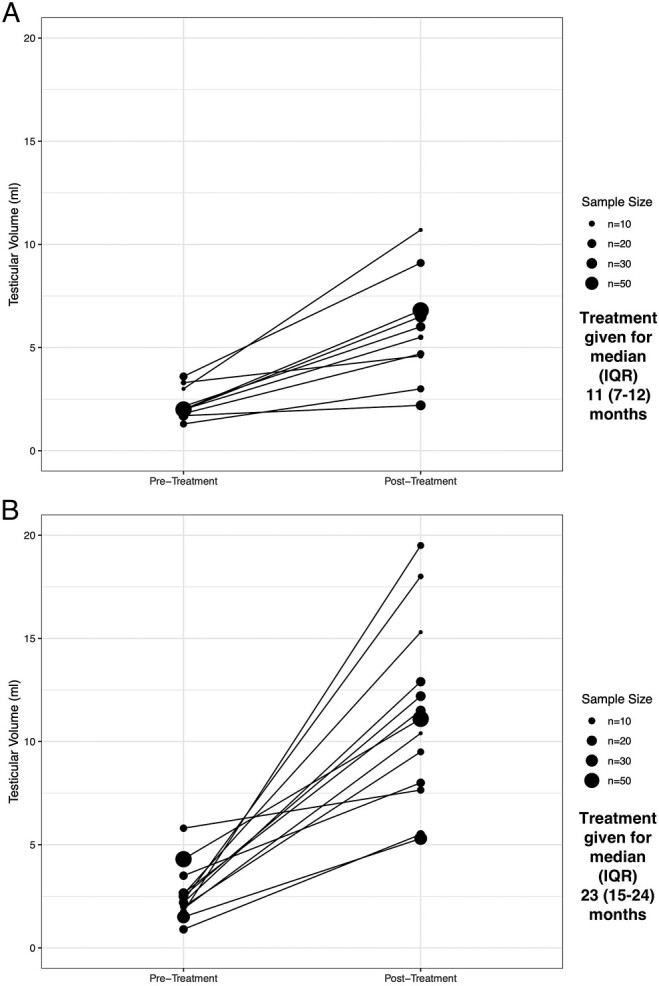
Plot of pre- and post-treatment average testicular volumes after treatment with (A) hCG and (B) hCG + FSH. Bilateral volumes were halved for plotting, and all methods of measurement were included. IQR, interquartile range.

Figure 2 and Table S4 describe the numerical change in testicular volumes among studies of the two most frequently prescribed treatments for pre-pubertal subjects, hCG alone, and hCG + FSH, in studies where 75%+ were pre-pubertal or had pre-pubertal onset of disease. From a broadly similar baseline, the combination of hCG + FSH appears to induce a greater increase in average testicular volumes than hCG alone.

#### Penile size

**Figure 3.**
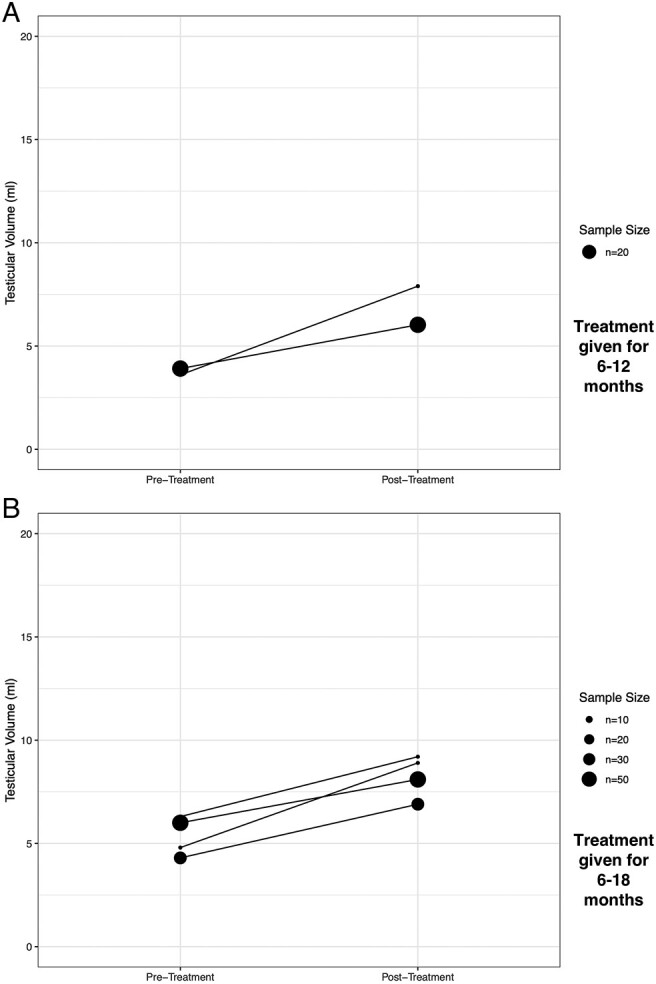
Plot of pre- and post-treatment average penile length after treatment with (A) hCG and (B) hCG + FSH.

Fourteen studies evaluated the impact of gonadotropins on penile size. All 20 analyses (100%) in these studies found that gonadotropins induced a significant increase in penile length (Figure S2). Increase in penile length appeared similar in those receiving hCG alone or in combination with FSH in the majority pre-pubertal studies (Figure 3 and Table S5).

#### Spermatogenesis

**Figure 4.**
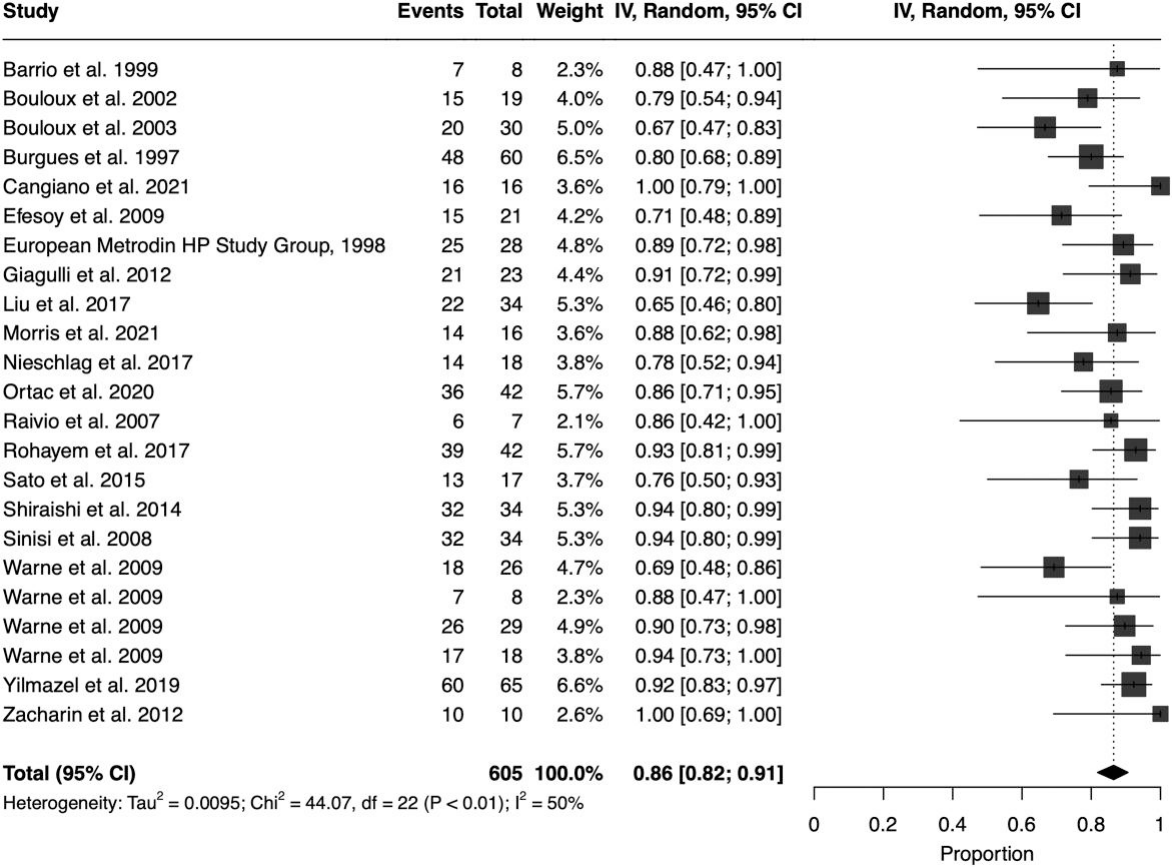
Meta-analysis of proportions for spermatogenesis achieved by hCG + FSH in patients with hypogonadotropic hypogonadism—pooled proportion (random effect) 0.86 (95% CI 0.82-0.91). CI, confidence interval.

We conducted a meta-analysis of the proportions of patients achieving spermatogenesis across the treatment groups of hCG, hCG + FSH, hCG + hMG, and GnRH (Figure 4 for hCG + FSH and Figures S3-S5 for other treatments) in all cohorts. There was substantial heterogeneity for hCG, with *χ*^2^ = 47.5, *P* < .01, *I*^2^ = 83%; for hCG + hMG, *χ*^2^ = 89.5, *P* < .01, *I*^2^ = 79%; and for GnRH, *χ*^2^ = 35.9, *P* < .01, *I*^2^ = 69%. There was moderate heterogeneity for hCG + FSH: *χ*^2^ = 44.1, *P* < .01, *I*^2^ = 50%.

For studies of hCG, the pooled proportion of patients achieving spermatogenesis using a random effect model was 40% (95% CI 25%-56%). For hCG + hMG, the pooled proportion was 76% (95% CI 68%-84%). For GnRH, the pooled proportion was 76% (95% CI 65%-86%). The highest proportions achieving spermatogenesis was with hCG + FSH, where the pooled proportion was 86% (95% CI 82%-91%). Spermatogenesis rates in the majority pre-pubertal cohorts reporting testicular volumes for hCG alone and hCG + FSH are also reported in Table S4.

### Biochemical parameters

Fifty analyses were conducted assessing the pre–post treatment difference in total testosterone concentrations; 98% of these (*n* = 49) reported a significant increase in testosterone concentrations after treatment with gonadotropins (Figure S6). Among studies measuring testosterone concentrations in nanograms per millilitre or nanograms per decilitre, the median pre-treatment average was 0.4 ng/mL (0.26-0.5), and the median post-treatment value was 4.7 ng/mL (3.9-6.5).

Sixteen studies included data on serum concentration of inhibin B in patients treated with gonadotropins. In general, treatment was described as resulting in increased inhibin B concentration. Among studies using concentrations of picograms per millilitre, the median pre-treatment value was 63 pg/mL (47-84) and the median post-treatment value was 103 pg/mL (99-121). Five out of seven analyses assessing significance found that there was a significant increase in inhibin B post-treatment.^31,34-36^

### Clinical outcomes in pre-pubertal cohorts with CHH/IHH never treated with androgens

A minority of studies (11 studies) reported results for patients with CHH or IHH who were previously untreated with androgens (either as whole cohort or as an analysed subgroup).^16,31,37-45^ These studies are summarized in Table S6. The median average age across these studies was 21 years. Median pre- and post-treatment testicular volumes were 1.7 and 7.9 mL across five studies reporting testicular volumes. Spermatogenesis rates were reported in four studies and ranged from 75% (3/4) to 100% (12/12).^31,38,41,45^

### Direct comparison of gonadotropin regimes

Five randomized trials and 18 non-randomized comparative studies of different gonadotropins passed quality screening. Of the five RCTs, the comparisons included hCG and recombinant FSH (rFSH), with comparison of twice and three-times per week dosing;^46^ GnRH, against pre-treatment with rFSH followed by GnRH;^47^ treatment with hCG and urinary FSH (uFSH), versus hCG and uFSH plus oral 40 mg zinc once/day;^48^ a randomized crossover study of uFSH and testosterone enanthate 250 mg once weekly, versus hMG and hCG;^49^ and hCG and sequential, versus continual, uFSH.^50^

There were very few studies directly comparing key treatment options to one another (eg, hCG versus hCG + FSH) with respect to pubertal outcomes. For testicular volume, three studies performed statistical analyses comparing the change in testicular volume induced by hCG and by testosterone, and all three found that hCG induced a greater increase in testicular volume (Tables S6 and S7).^37,51,52^ One of these studies also found that hCG was superior to testosterone with regard to spermatogenesis.^52^ Five studies compared the utility of hCG + hMG versus GnRH; three found no significant difference in testicular volume between groups, one found an inconsistent effect, and one found that GnRH was superior.^30,53-56^ With regard to post-therapy testosterone concentration, three out of five studies comparing hCG + hMG versus GnRH found that hCG + hMG was superior.^30,53-56^

### Predictors of poor response

#### Initial testicular volume

Overall, 27 unique studies, with a median sample size of 26 patients, performed statistical analyses as to the role of initial testicular volume as a predictor of poor response to gonadotropins. On review, 16 comparisons found that lower initial testicular volume was related to poorer spermatogenesis outcomes, and 12 did not find an association. There was a trend towards studies with higher sample sizes showing more significant results (Figure S7). Six unique studies evaluated the impact of initial testicular volume on final testicular volume, and five out of six found that smaller initial testicular volumes were associated with a smaller final testicular volume after treatment.^16,57-60^ One study found no significant association.^61^

#### Cryptorchidism

All five studies (median sample size 26) that evaluated the impact of a history of cryptorchidism on post-treatment testicular volume found that cryptorchidism resulted in significantly lower post-treatment testicular volumes.^28,36,38,62,63^ Five unique cohorts evaluated the effect of cryptorchidism on spermatogenesis outcomes and performed statistical analyses, with mixed results: two cohorts found cryptorchidism was significantly associated with lower attainment of spermatogenesis,^64,65^ and three found no significant relationship.^38,66,67^ Seven other studies described that patients with cryptorchidism had lower or delayed attainment of spermatogenesis but did not report statistical significance.^16,35,36,46,62,68,69^ In general, studies did not describe whether gonadotropins induced testicular descent (this occurred in one case in one study^70^)—the majority of patients had previously resolved cryptorchidism treated with orchidopexy in childhood.

#### Kallmann syndrome versus normosmic CHH

Overall, 16 studies evaluated the difference in outcomes between patients with Kallmann syndrome and normosmic CHH/IHH. Five out of eight statistical analyses evaluating post-treatment testicular volumes found no significant difference between Kallmann syndrome and normosmic CHH/IHH,^29,71-74^ and three studies found that normosmic patients had significantly larger final testicular volumes.^36,53,75^ Four out of five studies analysing spermatogenesis-related outcomes reported no significant difference between Kallmann and normosmic cohorts,^72,73,75,76^ and one reported that outcomes were worse in patients with olfactory impairment.^77^ Other studies described differences in spermatogenesis outcomes that generally appeared worse in Kallmann than in normosmic cohorts, but without statistical analyses.^16,31,32,67,71,74,78,79^

### Adverse effects

Thirty-six unique studies reported the presence or absence of adverse events. Adverse events most frequently described were gynaecomastia, acne, and injection site reactions/pain. The meta-analysed pooled proportion of each key adverse effect is reported in Table 1 and illustrated in Figure 5, with forest plots in Figures S8-S19. There was a high degree of heterogeneity for most outcomes.

**Figure 5.**
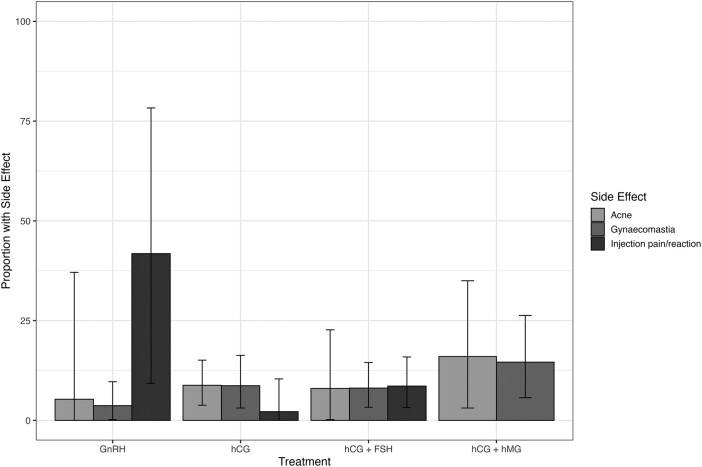
Percentage of patients experiencing key adverse effects: meta-analysis of proportions (with 95% confidence interval) using a random effects model. FSH, follicle-stimulating hormone; GnRH, gonadotropin-releasing hormone; hCG, human chorionic gonadotropin; hMG, human menopausal gonadotropin.

**Table 1.** Pooled percentage of patients experiencing key adverse effects by gonadotropin.

Treatment	Adverse effect	# of studies	Pooled percentage
hCG	Gynaecomastia	5^29,37,62,76,80^	9% (95% CI 3%-16%) *χ*^2^ = 7.66, *P* = .10, *I*^2^ = 48%
	Acne	3^29,76,80^	9% (95% CI 4%-15%) *χ*^2^ = 0.57, *P* = .75, *I*^2^ = 0%
	Injection site reaction/pain	2^37,76^	2% (95% CI 0%-10%) *χ*^2^ = 1.60, *P* = .21, *I*^2^ = 37%
hCG + FSH	Gynaecomastia	8^16,27,46,57,76,81-83^	8% (95% CI 3%-14%) *χ*^2^ = 17.46, *P* = .01, *I*^2^ = 60%
	Acne	6^16,33,46,57,76,81^	8% (95% CI 0%-23%) *χ*^2^ = 48.23, *P* < .01, *I*^2^ = 90%
	Injection site reaction/pain	5^33,46,63,76,84^	9% (95% CI 3%-16%) *χ*^2^ = 5.48, *P* = .24, *I*^2^ = 27%
	Anti-FSH antibodies	5^46,63,76,85,86^	0% (95% CI 0%-1%) *χ*^2^ = 0.36, *P* = .99, *I*^2^ = 0%
hCG + hMG	Gynaecomastia	4^53,55,75,87^	15% (95% CI 6%-26%) *χ*^2^ = 11.43, *P* < .01, *I*^2^ = 74%
	Acne	3^30,56,75^	16% (95% CI 3%-35%) *χ*^2^ = 9.47, *P* < .01, *I*^2^ = 79%
	Injection site reaction/pain	1^30^	One study only—0 of 20
GnRH	Gynaecomastia	4^29,54,55,71^	4% (95% CI 0%-10%) *χ*^2^ = 1.52, *P* = .68, 0, *I*^2^ = 0%
	Acne	2^54,56^	5% (95% CI 0%-37%) *χ*^2^ = 4.12, *P* = .04, *I*^2^ = 76%
	Injection site reaction/pain	5^30,53,56,64,88^	42% (95% CI 9%-78%) *χ*^2^ = 17.54, *P* < .01, *I*^2^ = 83%

Abbreviations: CI, confidence interval; FSH, follicle-stimulating hormone; GnRH, gonadotropin-releasing hormone; hCG, human chorionic gonadotropin; hMG, human menopausal gonadotropin.

## Discussion

Gonadotropin therapy to induce puberty in adolescents and young men with HH is hypothesized to lead to improved outcomes for testicular development and spermatogenesis as compared to classical therapy with testosterone. However, there is little consensus or standardization in the use of gonadotropins in this age group. This systematic review and meta-analysis therefore represents the most comprehensive synthesis of pubertal outcomes in patients with HH, encompassing testicular volume, penile length, biochemical outcomes, and spermatogenesis. We found a high level of study heterogeneity in treatment selection, dosing and duration, as well as study-level bias. However, there remained striking evidence of the efficacy of gonadotropins for induction of puberty, with >95% of pre–post statistical analyses describing significant increases in testicular volume, penile length, and testosterone. We found that combination therapy of hCG + FSH appears to lead to a demonstrable increase in testicular volumes, and spermatogenesis rates, above hCG alone, although there was substantial study heterogeneity. Whilst many studies included mixed populations of patients and only a small minority of studies were restricted to only patients with CHH/IHH not previously treated with androgens, the findings in this subgroup mirrored those in the wider analysis. Our findings provide robust synthesized evidence that underlines the importance of HH as a rare endocrine disease where appropriate medical therapy can have a remarkable impact, to reverse the clinical phenotype and restore fertility for the majority of patients.

With regard to treatment safety, we determined pooled proportions experiencing key adverse effects, which were acne, gynaecomastia, and injection site reaction or pain. For some treatment options and effects, 95% CIs for these estimates remained large. However, these data will be vital for counselling patients. In general, across all included studies, gonadotropin therapy was well tolerated.^30,53,75^

With regard to predictors of poor response, studies consistently found that the presence of small initial testicular volumes or cryptorchidism at initiation of treatment was related to smaller final testicular volume. Evidence regarding the impact of these factors on rates of spermatogenesis was more mixed. These data highlight the necessity for formalized guidelines for patients with more severe CHH, potentially with a period of pre-treatment with FSH to increase Sertoli cell numbers prior to combined gonadotropin therapy.^47^

In the context of greater awareness and more thorough diagnostic testing, paediatric and adult endocrinologists may see increasing numbers of patients presenting with HH, with increasing demand for appropriate treatment to induce puberty and fertility. However, what is also a striking finding from this review is the high variability in dosing strategies and treatment options employed globally. This illustrates that best practice guidelines and prospective longitudinal data collection, facilitated by the use of international electronic registries, are required. One best practice guideline, developed by four academic centres in the UK, provides a framework for induction of puberty using rFSH and hCG in adolescents aged 12 years and above.^89^

### Limitations

There are several limitations to this systematic review. Meta-analyses of observational data are highly liable to bias due to substantial confounding between studies, and this was evident in our review through our findings of variability in cohorts, dosing, and treatment duration, as well as outright statistical heterogeneity. Metelli and Chaimani^90^ acknowledged the various risks of bias that arise from meta-analysing observational data and also advised that they may be acceptable when observational data represent the only source of available evidence. We conducted our analyses with acknowledgement of these caveats.

As with any systematic review, publication bias is present. Participant data may have been duplicated across more than one study, although we screened and excluded where data this was clearly the case. We separately analysed FSH and hMG, with the latter assumed to contain FSH and LH in combination, but some misclassification may have occurred depending on the terminology used by the study authors.^19^ Pubertal status was reported in studies in varying ways and can be influenced by pre-treatment as well as HH itself. Various studies described prior treatment before study enrolment, in particular with testosterone, although a prior systematic review found that testosterone pre-treatment does not impact the efficacy of gonadotropins.^17^ We did not exclude studies in which participants were pre-virilized, and we also included studies in which patients with IHH/CHH were studied alongside patients with acquired HH, which may reduce the specificity of our conclusions. This approach reflects the fact that most studies included broad cohorts of patients with HH with various causes. Studies may have collected adverse effect data inconsistently. It was not possible to synthesize studies and identify time taken until maximal testicular volumes/sperm counts were achieved, due to *a priori* differences in study design and follow-up, with differing baseline characteristics, lack of reporting of subgroups, and differing lengths of follow-up. Finally, we only included studies published or translated in English, which may bias the results.

## Conclusions

This systematic review and meta-analysis provides convincing evidence regarding the efficacy of gonadotropins for the attainment of pubertal outcomes and spermatogenesis in patients with HH. However, we identified substantial heterogeneity globally with regard to treatment choice, dose, duration, and outcomes assessed. Guidelines and ongoing research are needed to determine optimal therapies and decision-making for patients with hypogonadotropic hypogonadism.

## Supplementary material

Supplementary material is available at *European Journal of Endocrinology* online.

## Supplementary Material

lvad166_Supplementary_Data

## Data Availability

The data that support the findings of this study are available from the corresponding author, S.R.H., upon reasonable request.
