# Supplementary material for: Gonadotropins for pubertal induction in males with hypogonadotropic hypogonadism: systematic review and meta-analysis
Source: Eur J Endocrinol. 2023 Dec 21;190(1):S1–S11. doi: 10.1093/ejendo/lvad166 (PMC10773669; doi:10.1093/ejendo/lvad166)
Supplement: lvad166_Supplementary_Data [file lvad166_supplementary_data.docx]

| **Supplementary Box 1: Search strategy** |
| --- |
| \| 1. \| (therap* or treatment$ or injection or intervention or medication or medicine or administ* or replacement).mp. [mp=ti, ab, hw, tn, ot, dm, mf, dv, kf, fx, dq, bt, nm, ox, px, rx, ui, sy, cw, tc, id, tm] \| \| --- \| --- \| \| 2. \| (male* or men or man or boy$ or XY or father$ or son$).mp. [mp=ti, ab, hw, tn, ot, dm, mf, dv, kf, fx, dq, bt, nm, ox, px, rx, an, ui, sy, cw, tc, id, tm] \| \| 3. \| (hypogonadotropic hypogonadism or hypogonadotrophic hypogonadism or Secondary hypogonadism or Central hypogonadism or gonadotropin-releasing hormone deficiency or gonadotrophin-releasing hormone deficiency or gonadotropin deficiency or gonadotrophin deficiency or kallmann syndrome).mp. [mp=ti, ab, hw, tn, ot, dm, mf, dv, kf, fx, dq, bt, nm, ox, px, rx, an, ui, sy, cw, tc, id, tm] \| \| 4. \| (FSH or LH or HCG or gnrh or follicle stimulating hormone or luteinizing hormone or luteinising hormone or gonadotropin* or gonadotrophin*).mp. [mp=ti, ab, hw, tn, ot, dm, mf, dv, kf, fx, dq, bt, nm, ox, px, rx, an, ui, sy, cw, tc, id, tm] \| \| 5. \| (pubert* or pubescen* or sperm* or tanner or fertil* or testic* or gonad* or inhibin or inhbb or antimullerian or anti-mullerian or AMH or testosterone).mp. [mp=ti, ab, hw, tn, ot, dm, mf, dv, kf, fx, dq, bt, nm, ox, px, rx, an, ui, sy, cw, tc, id, tm] \| \| Final search: \| 1 and 2 and 3 and 4 and 5 \| |

| **Supplementary Table 1: Summary of included studies – congenital/idiopathic HH cohorts** | | | | | | | |
| --- | --- | --- | --- | --- | --- | --- | --- |
| **Citation** | **Country** | **Population description** | **Number of participants** | **Population age at baseline** | **Pubertal status at baseline** | **Therapy** | **Overall quality score [NHLBI for observational, ROB 2.0 or ROBINS-I otherwise]** |
| Abbasi et al. 2008 ^1^ | Iran | Azoospermic infertile men with idiopathic hypogonadotropic hypogonadism | 56 | Mean 31 years (range 20-50) | Not stated | 3 months of hCG, 5000 IU three times a week, then a combination of hCG and 75 IU hMG three times a week up to 24 months (n=56) | 9 |
| Aydogdu et al. 2013 ^2^ | Turkey | Never-treated patients with idiopathic hypogonadotropic hypogonadism | 77 | Mean 21 +/- 1.4 years | Mean Tanner stage 1.3 +/- 0.5 | Three treatment groups - one of hCG 5000 IU twice weekly, one of IM testosterone (Sustanon Ⓡ 250mg) once every 3 weeks, one of daily testosterone gel (Testogel Ⓡ 250mg/5g) | Moderate |
| Bayram et al. 2015 ^3^ | Turkey | Idiopathic Hypogonadotropic Hypogonadism | 16 | 27.5 +/- 10.5 years | 100% prepubertal onset | Intramuscular injections of hCG 1500 IU administered 3 times weekly for 6 months group 1 (n=16) | 8 |
| Boepple et al. 2008 ^4^ | United States | Men with idiopathic hypogonadotropic hypogonadism | 12 | 25-45y | IHH patients 100% failure to go through puberty by age 18 | Long-term pulsatile GnRH therapy | 8 |
| Bouloux et al. 2002 ^5^ | Three Europea  n countries | Severe isolated hypogonadotropic hypogonadism (a combination of idiopathic, Kallmann) | 19 | Mean 25.9 +/- 7.7 years | 100% testicular volume <4ml | hCG 2,000 IU subcutaneous injection twice weekly for 6 months, then 18 months of hCG and rhFSH 150 IU subcutaneous injection three times a week, both with dose titration (n=19) | 8 |
| Cangiano et al.. 2021 ^6^ | Italy | Congenital hypogonadotropic hypogonadism | 19 (7 Kallmann) | Aged 14-23 | 15/19 BTV <8ml = 78.9% | FSH 75 IU thrice/week and hCG 250 IU 2 or 3 times/week for most patients. Thirteen patients underwent pretreatment of 75 IU FSH three/week for 4 months before combination. (n=19) | 8 |
| Chen et al. 2019 ^7^ | China | Isolated (congenital) hypogonadotropic hypogonadism (Kallmann or normosmic) | 107 (49 with Kallmann) | hCG/TU group : 21.8 +/- 3.7 years    hCG group: 22.7 +/- 4.1 years | Genital Tanner stage of 1.8±1.0 or 1.7±0.7 | Group 1 (n=54) intramuscular injection of 2000 IU hCG injected twice per week together with oral T undecanoate soft capsules, 40 mg twice daily. In the first 6 months, hCG doses were adjusted according to patients' serum T levels. The maximum dose of hCG used in our study was 8000 IU twice per week.  Group 2: (n=53) intramuscular injection of 2000 IU hCG injected twice per week. | Moderate |
| Chen et al. 2020 ^8^ | China | Patients with congenital hypogonadotropic hypogonadism with poor responses to human chorionic gonadotropin | 110 | Age 15-43 years | 60% TV <4ml | hCG 2000 IU twice a week, with dose titration, for minimum 12 months (n=110) | 11 |
| Christiansen et al. 2002 ^9^ | Denmark | Idiopathic Hypogonadotropic Hypogonadism or Kallmann Syndrome | 11 (3 with Kallmann) | Median age 28 years (range 22-33) | 64% had TV <4ml at baseline; all absent of puberty or pubertal arrest | 5ug GnRH/pulse and dosage gradually increased until the serum testosterone was in normal range (n=11) | 9 |
| Delemarre-Van de Waal and Odink 1993 ^10^ | The Netherlands | Isolated idiopathic hypogonadotropic hypogonadism or Kallmann syndrome | 19 (7 with Kallmann) | 15.2-23.0 | 100% of patients had absence of puberty or pubertal arrest | 1) GnRH infusion at 2 ug per pulse every 90 minutes then hCG if in the presence of azoospermia and adequate testicular volume (n=19)  2) GnRH infusion with increasing pulse frequency and pulse dose every 3 months over 18 months (n=3) | 8 |
| Dwyer et al. 2013 ^11^ | United States | Congenital hypogonadotropic hypogonadism (GnRH-deficient men with prepubertal testes (4 mL), no cryptorchidism, and no prior gonadotropin therapy) | 13 | Mean 28 +/- 2 in rFSH pre-treatment group, mean 24 +/- 2 in GnRH only group | All had prepubertal testes <4 mL | Either 24 months of pulsatile GnRH (n=6) or 4 months pretreatment with rFSH (75-150 IU SC OD) followed by 24 months of pulsatile GnRH (n=7) | Some concerns |
| European Metrodin HP Study Group, 1998 ^12^ | Europe (eight clinical centres) | Primary complete isolated hypogonadotropic hypogonadism | 28 (11 with Kallmann) | Mean 22.8 +/- 6.1 years | 100% had lack of spontaneous puberty; 17 were prepubertal | hCG 2000 IU 2x/week for 3-6 months then FSH 150 IU SC 3x/week + hCG 2000 IU 2x/week (or dose required to normalise serum testosterone) for 18 months (n=28) | 9 |
| Fahmy et al. 2004 ^13^ | Egypt | Azoospermic men with hypogonadotropic hypogonadism (Kallmann or isolated) unresponsive to gonadotropin therapy | 15 (1 Kallmann) | 29 to 52 years (mean 38.71+/-6.2). | 100% history of failed puberty, with testes 6-8ml in volume | 75 IU hMG thrice weekly and 5000 IU hCG 1-2 times per week for >6 months (n=15) as pre-treatment, continuation of gonadotropic therapy for an additional 6 months for n=9 in addition to ICSI | 7 |
| Fan et al. 2022 ^14^ | China | Congenital hypogonadotropic hypogonadism | 52 | Mean 20.7 +/- 5.3 years | 100% no spontaneous puberty by age 15 and Tanner stage 1-2 | SC pulsatile GnRH via infusion pump, 10 ug every 90 minutes for median 13.5 (11.3-24 months) (n=32) | 10 |
| Fuse et al. 1996 ^15^ | Japan | Hypogonadotropic hypogonadism (idiopathic or Kallmann) | 16 (3 with Kallmann) | Aged 17-33 years | 50% TV <4ml; all had prepubertal onset | hCG 1000 IU of hCG+ hMG 75 IU, twice per week, with dose titration of both (n=16) hCG alone (n=8) hCG+hMG (n=8) | 9 |
| Gong et al. 2015 ^16^ | China | Peri-pubertal boys with hypogonadotropic hypogonadism, either Kallmann or normosmic idiopathic | 35 (23 with Kallmann) | Range 10-16 | Over 80% of patients in each group were Tanner Stage I at baseline | 1) 1000 IU hCG IM twice per week for 3 months, then once every other day for 3 months, then increased to 2000 IU twice per week / 1500 IU every other day for 3 months, then 2000 IU every other day for three months (n=22)  2) 8-10 ug of GnRH SC injection via pump every 90 minutes (n=12) | Moderate |
| Hao et al. 2020 ^17^ | China | Noncryptorchid patients with partial and complete congenital hypogonadotropic hypogonadism | 122 | 20.6 ± 4.3 years | 100% absent puberty by 18 years; 83/122 had complete CHH with TV 1.8 +/- 0.7 ml | hCG 2000-5000 U IM twice weekly for 3 months followed by IM hMG 75-150 U twice weekly combined with hCG, titrated to testosterone (n=122) | 10 |
| Hao et al. 2021 ^18^ | China | Congenital hypogonadotropic hypogonadism | 28 | 24.23 +/- 4.63 years | Not stated | Pulsatile GnRH (n=10) SC via portable pump with 10ug administered every 90 minutes | 6 |
| Hosseinifar et al. 2013 ^19^ | Iran | Idiopathic hypogonadotropic Hypogonadism | 58 | 34.75 +/- 1.10 years (in the responders group n=20)   35.31 +/- 0.75 years (in the non responders group n=38) | Not stated | 5000 units hCG intramuscular, 3 times per week, followed by 75 IU hMG intramuscular 2 times per week. | 9 |
| Kilciler et al. 2002 ^20^ | Turkey | Idiopathic hypogonadotropic hypogonadism | 14 (0 Kallmann) | Mean age, 21.1+/- 1.2 years | 100% failed to have spontaneous puberty by age 18 years | hCG 5000 IU three times a week IM + hMG (Pergonal, 75 IU FSH plus 75 IU LH) (n=14) | 8 |
| Kirk et al. 1994 ^21^ | United Kingdom | Idiopathic isolated hypogonadotropic hypogonadism | 26 (14 with Kallmann) | Aged 17-48 (median 21.5) years | Mean TV was < 3ml in all patients | 1) hCG 2000 IU twice weekly for 6 weeks then 1000 IU twice weekly (n=23);   2) Combined hCG 500 IU IM twice weekly and hMG 300 IU IM three times weekly for 6 months minimum (n=9) | 8 |
| Li et al. 2020 ^22^ | China | Patients with congenital hypogonadotropic hypogonadism with and without the FGFR1 mutation | 39 (28 with Kallmann) | 20.00 (18.75-24.75) in FGFR1 group and 24.12+/- 5.75 in FGFR1 negative group | 100% had no puberty development by 18 years | hCG IM 2000-3000 IU and 75 IU hMG twice a week with dose titration (n=22)  SC gonadorelin at 10ug/90 minutes with dose titration (n=17) | 10 |
| Liu et al. 2019 ^23^ | China | Congenital hypogonadotropic hypogonadism patients with and without cryptorchidism | 223 (111 with Kallmann) | Mean - 18.9 +/- 4.6 years (cryptorchidism group), mean 20.4 +/- 4.8 (others) | Not stated. All patients had CHH. Basal TV mean between 1.6-2.2 ml. | Combined hCG (2000 twice weekly) and hMG (75U twice weekly) with dose titration to hCG 2500-5000U and hMG 150U. | 10 |
| Liu et al. 2016 ^24^ | China | Congenital hypogonadotropic hypogonadism with azoospermia without puberty development | 223 (111 with Kallmann) | 22.4 +/- 2.3 years | 100% "without puberty development" | IM hCG 2000-5000 IU twice weekly for 6 months then hMG (75-150 U) IM twice weekly (n=223) | 10 |
| Liu et al. 2017 ^25^ | China | Isolated hypogonadotropic hypogonadism | 67 (27 with Kallmann) | The mean age of the IHH patients was 24.1 ± 4.9 years in Group A (hCG/FSH) and 22.3 ± 4.0 years in Group B (FSH/zinc). | 100% without spontaneous puberty | Group A: 2000 U hCG twice a week for 18 months and 75 U uFSH three times a week every other 3 months for 6-18 months Group B: 2000 U hCG twice a week for 18 months and 75 U uFSH three times a week every other 3 months for 6-18 months PLUS oral 40mg/day zinc for 18 months | Some concerns |
| Liu et al. 2021 ^26^ | China | Congenital hypogonadotropic hypogonadism | 41 (24 with Kallmann syndrome) | Between 12 and 18 years | Some with no spontaneous puberty, some with delayed progression. | hCG/HMG n=20 group: 1000-2000 IU hCG IM injection for 3 months once every other day or twice per week, titrated to testosterone. Subsequently hMG containing 75 U of FSH and 75 U of LH was added once daily, titrated to testosterone. Duration was 6 to 21 months.  GnRH group n=21: 8 to 10 ug of GnRH (200 ug/ml) were injected SC every 90 min via an injection pump. Duration was 6-26 months. | Moderate |
| Mao et al 2017 ^27^ | China | Congenital hypogonadotropic hypogonadism | 202 (95 with Kallmann syndrome) | 21.5 +/- 3 (hCG/HMG group), 27.1 +/- 7 (GnRH group) | All patients (100%) had absence of pubertal development by age 18 | Group 1: 2000 IU hCG 2 times/week for 3 months then continued treatment combined with hMG 75-150 U 2 times/week for 12 months (n = 182)   Group 2: 10 ug per 90 min SC GnRH, dose adjusted to target LH and FSH between 5-10 IU/l (n = 20) | Serious |
| Musabak et al. 2003 ^28^ | Turkey | Untreated idiopathic hypogonadotropic hypogonadism | 15 (0 with Kallmann) | Mean 21.3 ± 1.5 years | All patients (100%) had absent spontaneous puberty by age 18 | hCG 2000 IU and human postmenopausal gonadotropin (hMG) containing 75 IU FSH and 75 IU LH three times a week for 6 months (n=15) | 8 |
| Nachtigall et al. 1996 ^29^ | United States | Men with idiopathic hypogonadotropic hypogonadism | 31 | Aged 18-45 | 21/31 had no history of partial or complete previous spontaneous puberty | Pulsatile GnRH administered SC every 2 hours via infusion pump at a dosage individualised to sustain normal testosterone | 8 |
| Nachtigall et al. 1997 ^30^ | United States | Men with classical GnRH deficiency, and men with adult-onset idiopathic hypogonadotropic hypogonadism that developed after puberty | 34 | Adult onset IHH: age, 27 to 57 years, mean 35 +/- 10 years; classic GnRH deficiency: age, 18 to 58 years | 0% of IHH post-pubertal group, not stated for GnRH deficiency group | Adult onset idiopathic hypogonadotropic hypogonadism SC GnRH via infusion pump every two hours for 6 months (n=5) and for <3 months (n=3); Testosterone (n=1); hCG (n=1). Classic GnRH deficiency: SC GnRH via infusion pump every two hours for 6 months (n=24) | 9 |
| Nie et al. 2021 ^31^ | China | Congenital hypogonadotropic hypogonadism patients with FGFR1 variants | 25 | / | All patients had absent or incomplete puberty by age 18 | hCG treatment + hMG treatment (n=14), testosterone (n=10), + GnRH (n=1) | 6 |
| Oktenli et al. 2003 ^32^ | Turkey | Idiopathic hypogonadotropic hypogonadism | 35 (0 Kallmann) | Mean 21.34+/-1.53 years | 100% failed to have spontaneous puberty by age 18 | hCG 2000 IU and hMG (75 IU FSH + 75 IU LH) three times a week for 6 months (n=35) | 9 |
| Ortac et al. 2020 ^33^ | Turkey | Congenital hypogonadotropic hypogonadism | 112 | Mean 27.9 (15-51) years | Not stated | IM hCG 1500 IU twice per week for 6 months. If the patient remained azoospermic at the end of 6 months, FSH 75-150 IU twice per week (rFSH or hMG), was added. | Moderate |
| Ortac et al. 2019 ^34^ | Turkey | Azoospermic men with idiopathic hypogonadotropic hypogonadism | 112 | Mean 27.9 +/- 6.6 years (range 15-51) | Not stated | IM hCG 1500 IU twice per week titrated to testosterone/testicular development for 6 months, then FSH 75-150 IU twice per week titrated to FSH levels (n=112) | 10 |
| Pitteloud et al. 2002 ^35^ | United States | Idiopathic hypogonadotropic hypogonadal men undergoing GnRH therapy for 12-24 months | 76 | Age 28 +/- 0.9 (absent puberty) 27 +/- 0.9 (partial) 34 +/- 2 (complete) | 52/76 had absent puberty (68%) | Pulsatile GnRH SC via pump at 2 hour intervals from 5-25 ng/kg with dose titration (n=76) | 11 |
| Rohayem et al. 2020 ^36^ | Germany | Congenital hypogonadotropic hypogonadism | 25 (14 Kallmann) | / | 100% of patients had history of absent or arrested spontaneous puberty | In prepubertal subjects, 500 IU hCG SC twice weekly up to a maximum of 3x1500 IU hCG SC/week titrated to testosterone. In post-pubertal subjects, 2x1000-1500 IU hCG SC/week.  After 3-6 months of hCG, rFSH was added at 3x150 IE SC/week until gonadal maturation achieved. Then switched to exogenous testosterone substitution by gel or IM injections. (n=25) | 9 |
| Saal et al. 1991 ^37^ | Germany | Hypothalamic/hypogonadotropic hypogonadism (IHH or Kallmann) | 16 (8 Kallmann) | Aged 18-34.5 years | 10/16 (62.5%) had testes <4ml | hCG SC 1500-10,000 IU/ week for 1-14 months (n=12) prior to combined gonadotropin therapy - hCG 3000-15000 IU/week titrated to testosterone, and hMG either 450 IU/week or 225 IU/week, with increase up to 750 IU/week (n=16) | 7 |
| Sanyal et al. 2016 ^38^ | India | Idiopathic hypogonadotropic hypogonadism | 31 (8 Kallmann) | Median 31.5 (19-59.6) years | Not stated | Group 1 (n=12) received intramuscular injection of testosterone enanthate (250mg every 4 weeks) but then were moved onto testosterone undecanoate (4ml every 3 months) once it was available.  Group 2 (n=14) 5000IU SC hCG twice weekly. Group 3 (n=5) had hCG 5000 IU twice a week with FSH 100 IU subcutaneously twice weekly. | Critical |
| Schaison et al. 1993 ^39^ | France | Hypogonadotropic hypogonadism without cryptorchidism (idiopathic or Kallmann) | 10 (6 Kallmann) | Age 23-35 years | Not stated, mean testicular volume 4 +/- 2ml | Crossover study: 150 U of hMG was administered three times weekly along with 1500 IU hCG for 24 months, followed by urinary FSH 150 IU three times a week plus testosterone enanthate 250mg IM once weekly, for 24 months (n=5) or the other way round (n=5) | Some concerns |
| Schopohl 1993 ^40^ | Germany | Male patients with idiopathic hypothalamic hypogonadism or Kallmann syndrome | 36 (17 Kallmann) | Mean 23.6 ± 7.3 years (gonadotropin group) 21.1 ± 3.0 years (GnRH group) | Not stated | 3x2500 IU hCG per week IM with dose titration. After 2-3 months, 2x 150 IU of hMG were added, with dose titration. (n=18) GnRH with pump started at 4ug/bolus up to 16ug/bolus if no pituitary response seen (n=18) | Moderate |
| Schopohl et al. 1991 ^41^ | Germany | Idiopathic hypothalamic hypogonadism | 36 (17 Kallmann) | Mean 23.6 ± 7.3 years (gonadotropin group) 21.1 ± 3.0 years (GnRH group) | 21 patients (60%) | 2500 IU hCG three times a week IM with dose titration, and 150 IU hMG after 2-3 months (n=18)  GnRH 4-16ug bolus every 90-120 minutes (n=18) | Moderate |
| Shah et al. 2021 ^42^ | India | Congenital hypogonadotropic hypogonadism | 35 (13 Kallmann) | 24.8 ± 6.1 years | 20/35 had severe phenotype and all had TV <3ml | Group 1: No prior TRT: All the patients were concurrently initiated on hMG and hCG, hMG 75-150 U 2-3/week and initial hCG 500 U twice weekly with dose titration. n=17  Group 2: Prior TRT, previously received testosterone ranging from 50mg once a month to 200-250 mg testosterone enanthate every 2-3 weeks. hMG and hCG, hMG 75-150 U 2-3/week and initial hCG 1000-2000U twice weekly with dose titration. n=18 | 11 |
| Sykiotis et al. 2010 ^43^ | USA | Men with idiopathic hypogonadotropic hypogonadism who received GnRH therapy | 90 | Aged >18 years | 16/23 had absent puberty | Pulsatile GnRH administered SC every 2 hours for at least 12 months. Dose initially 5-25 ng/kg per pulse and progressively increased (max. <800 ng/kg) to achieve mid-normal serum T levels (300-500 ng/dl). | 10 |
| Trabado et al. 2011 ^44^ | France | Men with congenital hypogonadotropic hypogonadism, treated or untreated | 280 (41 Kallmann) | Mean 28.8+/- 10.1 (CHH) | All absent or incomplete by age 17 | Combination therapy of hCG (1500 IU IM twice/three times weekly) and recombinant/extractive human FSH (150 IU SC twice/three times weekly) n=88  Testosterone enanthate (n=101) 250mg IM every 3 weeks | Serious |
| Vicari et al. 1992 ^45^ | Italy | Males with isolated/congenital hypogonadotropic hypogonadism | 17 | Mean 21.8 +/- 1.4 (18-37) | All had failure of puberty by age 18; 47% had baseline TV <4ml | hCG 1500IU IM, three times a week to reach maximal testicular volume, then one ampoule of hMG (containing 75 IU FSH and 75 IU LH) was added, twice weekly | 9 |
| Yesilova et al. 2000 ^46^ | Turkey | Idiopathic hypogonadotropic hypogonadism | 29 (0 Kallmann) | Mean 21.71 +/- 1.49 years | 100% had failure to enter spontaneous puberty by age 18 | All patients (29 IHH men) 2000 IU hCG and hMG containing 75 IU FSH and 75 IU LH three times a week for 6 months | 8 |
| Yilmazel et al. 2019 ^47^ | Turkey | Patients with idiopathic hypogonadotropic hypogonadism | 65 (0 Kallmann) | Mean 19.8 years | 35/65 patients had initial TV <4ml | 1500-2500 IU hCG twice a week for 3-6 months followed by addition of u/rFSH 150 IU 2-3 times a week up to 450 IU twice a week | 9 |
| Zhang et al. 2021 ^48^ | China | Males with congenital hypogonadotropic hypogonadism | 73 (46 Kallmann) | Mean 25.04 +/- 5.35 years | Not stated (cohort included partial or absent secondary sexual characteristics) | Group 1: Gonadorelin (GnRH analogue) 10ug every 90 minutes Group 2: Combined gonadotropin therapy (hCG and hMG) hCG initially injected IM for first 3-6 months (2000 IU 3-times-weekly) then hMG (75-150 IU 3-times-weekly) added for 3 months, cyclically repeated.  Group 3: hCG monotherapy (2000 IU IM. 1-3 times weekly) Group 4: Testosterone undecanoate orally or IM | 8 |
| Zhang et al. 2015 ^49^ | China | Idiopathic hypogonadotropic hypogonadism | 67 (30 Kallmann) | Mean 23.1 +/- 5.1 years in continual FSH group, and 24.1+/- 4.9 years in sequential FSH group | Average baseline genital Tanner stage was 1.6-1.7 across groups | All patients received 2000 U hCG twice a week from start to 18th month, IM. Group A: 75 U uFSH three times a week from 6-18th month, IM (n=33) and Group B: 75 U uFSH three times a week from 6-18th month, every other 3 months, IM (n=34). | Low |
| Zhang et al. 2019 ^50^ | China | Azoospermic men with congenital hypogonadotropic hypogonadism | 28 (16 Kallmann) | hCG/hMG group: 24 ± 5 (16-34) years Pulsatile GnRH group: 27 ± 3 (23-32) years | Not stated (included absent and delayed puberty) | Group 1: Pulsatile gonadorelin Pump (PCP) n=10. Gonadorelin (GnRH analogue) 10 ug every 90 min SC via infusion pumps with dose titration Group 2 hCG/hMG combined n=18. hCG IM 3-6 months 2000 IU three times per week until the serum testosterone level was >3 ng/ml or testicular volume = 3 ml. Then hMG (75 IU three times per week) was added for 3 months, followed by hCG alone again for another 3 months and then HCG +HMG for an additional 3 months cyclically. | Serious |
| Zhao et al. 2013 ^51^ | China | Idiopathic hypogonadotropic hypogonadism | 74 (38 Kallmann) | Population age 27.3 ± 3.4 years | 93.2% had absent pubertal development | hCG or hCG + hMG were combined in gonadotropin group (n=37)  hCG + Testosterone or hCG + hMG + testosterone (n=25) | Serious |

| **Supplementary Table 2: Summary of included studies – mixed cohorts** | | | | | | | |
| --- | --- | --- | --- | --- | --- | --- | --- |
| **Citation** | **Country** | **Population description** | **Number of participants** | **Population age at baseline** | **Pubertal status at baseline** | **Therapy** | **Overall quality score [NHLBI for observational, ROB 2.0 or ROBINS-I otherwise]** |
| Barrio et al. 1999 ^52^ | Spain | Hypogonadotropic hypogonadism (isolated hypogonadotropic hypogonadism and panhypopituitarism-related hypogonadotropic hypogonadism) | 14 (4 with Kallmann) | Group A (IHH group): 15.44 +/- 1.97 years. Group B (PHH): 18.1 +/- 3.24 years. | 100% prepubertal | Both groups (Group A, n=7 isolated hypogonadotropic hypogonadism) (Group B, n=7 panhypopituitarism-associated HH) were treated with IM injections of hCG at a dosage of 1,000- 1,500 IU every alternate day of the week. The optimal dosage of hCG was regulated by monitoring the serum testosterone level every 3 months and adjusting the dosage accordingly.   Both groups were treated with subcutaneous injections of a purified preparation of FSH at a dosage of 75-100 IU. The FSH dosage was increased up to 100 IU three times a week if testicular growth and the sperm count were unsatisfactory. | 9 |
| Bouloux et al. 2003 ^53^ | 6 centres across Australia, Denmark, Germany and the United Kingdom | Hypogonadotropic hypogonadism – a combination of idiopathic, Kallmann, and other causes | 49 (15 with Kallmann) | Group A (225 IU twice a week rFSH) - mean 30.1 years (5.6); or Group B (150 IU rFSH thrice a week) - mean 30.7 years (8.0) | Not stated | All patients 1500 IU hCG twice a week with titration to testosterone levels for 16 weeks. Subsequently, Group A: 225 IU twice a week FSH SC for 48 weeks (n=15). Group B: 150 IU thrice a week FSH SC for 48 weeks (n=15. | Some concerns |
| Bouvattier 1999 ^54^ | France | Hypogonadal males with wish to increase their testicular volume; a combination of idiopathic, Kallmann, congenital or acquired hypopituitarism, and one with Prader Willi | 37 (7 with Kallmann) | 16.4 +/- 1.8 | 89% had initial TV <4ml | hCG 1500IU i.m. twice-weekly for 6 months  Then hCG + hMG 75mg three-times-weekly for 2 years  n=37 | 9 |
| Buchter et al. 1998 ^55^ | Germany | Patients with hypothalamic disorders (idiopathic hypogonadotropic hypogonadism, Kallmann syndrome) and hypopituitarism | 42 (10 with Kallmann syndrome) | Mean age of the patients of group Ia (IHH+ Kal, GnRH) was 30.1 +/- 5.0 years (19- 35 years), in group Ib (IHH+Kal, hCG+hMG) mean age was 29.1 +/-7.2 years (21-43 years), while in group II (hypopituitarism, hCG/hMG) patients had a mean age of 34.1 +/- 6.1 years (23-48 years) | Not stated | 1a) Pulsatile GnRH 5-20ug every 120 minutes subcutaneously (n=6)  1b) IM/SC hCG 1000-2500 iU twice a week and 75-150 IU hMG three times a week in 20 courses (n=18)  2) IM/SC hCG 1000-2500 iU twice a week and 75-150 IU hMG three times a week in 30 cycles (n=21) | Serious |
| Burgues et al. 1997 ^56^ | Spain | Hypogonadotropic hypogonadism (combination of idiopathic, Kallmann, and hypopituitarism) | 60 (16 with Kallmann syndrome) | Mean 26.3 +/- 0.85 years | 86.70% had prepubertal onset | SC injection of FSH-HP 150 IU three times a week and hCG 2500 IU twice a week for 6 months minimum (n=60) | 10 |
| Celik et al. 2021 ^57^ | Turkey | Hypogonadotropic hypogonadism (congenital, Kallmann, and acquired) | 36 (2 with Kallmann) | 28.5 +/- 8.5 years | Baseline mean TV was 1.6 ml for hCG group | hCG 1000-2000 IU, 2-3/ week (n=17)  IM Testosterone 250mg every 21 days with dose titration (n=19) | Serious |
| Chen et al. 2021 ^58^ | Taiwan | Azoospermic men (congenital and acquired) with hypogonadotropic hypogonadism | 17 (4 with Kallmann) | 36.1 +/- 3.5 years | 35.3% TV <4ml | hCG 3000 IU SC twice a week for 24 weeks, with addition of hMG 75 IU twice per week for those who did not initiate spermatogenesis | 9 |
| Delemarre-Van de Waal 1993 ^59^ | The Netherlands | Hypogonadotropic hypogonadism (idiopathic, Kallmann, tumour or combined pituitary deficiency) | 38 (11 with Kallmann) | Mean age 18.9 years (range 13.4-26.0) | 24/38 had mean testicular volume <4ml at baseline | GnRH via infusion pump via indwelling catheter of between 2-10-20ug per pulse every 90 minutes (n=36), with patients with poor results having hCG 1500-3000 IU twice a week IM (n=24) | 9 |
| Depenbusch et al. 2002 ^60^ | Germany | Gonadotropin deficiency due to idiopathic hypogonadotropic hypogonadism (IHH), Kallmann syndrome or pituitary insufficiency | 13 (4 with Kallmann) | Range 19-38 | Not stated | Pre-treatment with testosterone, then hCG/hMG therapy with 150 IU hMG subcutaneously 3x/week and adapted hCG doses ranging from 500 IU twice a week to 2500 IU twice a week, with maintenance via hCG once spermatogenesis initiated (n=12) and pulsatile GnRH (n=1) | 7 |
| Efesoy et al. 2009 ^61^ | Turkey | Infertile men, subgroup with hypogonadotropic hypogonadism | 21 | 23.19 +/- 7.79 years | Not stated | hCG 1500 IU 2-3 times per week until serum testosterone in normal range then 100-150 IU of FSH 2-3 times per week (n=21) | 9 |
| Farhat et al. 2010 ^62^ | Saudia Arabia | Congenital or acquired hypogonadotropic hypogonadism | 87 (12 with Kallmann) | Median 28 years (18-64) | Not stated | IM hCG 1500u and IM hMG 75u three times a week, with dose increase if ongoing poor sperm production, plus IM testosterone enanthate (variable dosing/regimen) (n=87) | 9 |
| Farshchi et al. 2009 ^63^ | Iran | Hypogonadotropic hypogonadism (idiopathic, Kallmann, and pituitary insufficiency) | 102 | 22.7 +/- 6.3 years | Average baseline testicular volume was 3.4 +/- 1.9ml | IM Testosterone enanthate 250mg once a month until normalisation of secondary characteristics (n=75), if not previously treated with testosterone, then hCG 1500 IU three times per week + hMG 75 IU SC three times per week until successful spermatogenesis (n=102) | 9 |
| Giagulli et al. 2012 ^64^ | Italy | Congenital, prepubertal-onset and postpubertal-onset hypogonadotropic hypogonadism | 23 (2 with Kallmann) | Median between 28-36 years across groups, age range 18-50 | 12/23 were prepubertal-onset HH | hCG 5000 IU/week IM and rhFSH 75 IU/day IM for a minimum of 12 months (n=23) | 9 |
| Gu et al. 2021 ^65^ | China | Hypopituitarism (acquired or congenital) leading to hypogonadism | 22 | 22.8 +/- 3.7 years | All referred for absent or incomplete puberty; baseline TV was 0.75ml (IQR 0.48-1.48) | Pulsatile GnRH via infusion pump, 10-12ug/90 minutes (n=22) | 9 |
| Guo et al. 1997 ^66^ | United Kingdom | Isolated hypogonadotropic hypogonadism (idiopathic, Kallmann, or acquired) | 10 (2 with Kallmann) | 26-46 (mean 34) | All presenting with delayed puberty; final status not stated | hCG ranging from 2000-4000 IU twice a week at baseline (n=10) then (n=6) commenced escalating dose from baseline of 2000 IU twice a week for 1 month, then 3000 IU for 1 month, 4000 IU for 1 month, 5000 IU for 1 month, 6000 IU twice per week for one month. Testosterone (Sustanon) 125-250mg every 2 weeks (n=3) during past two years. | 8 |
| Huang et al. 2022 ^67^ | China | Congenital hypogonadotropic hypogonadism and pituitary stalk interruption syndrome | 64 (39 with Kallmann) | 24.6 +/- 5.5 years (CHH) | All CHH patients (n=64) had no/arrested puberty over age 16 years | Pulsatile gonadorelin (GnRH agonist) SC at 10ug/90 minutes with adjustment of dose to LH concentrations (n=64) | 10 |
| Ishikawa et al. 2007 ^68^ | Japan | Hypogonadotropic Hypogonadism (primary or acquired) | 26 (2 with Kallmann) | 26.0 +/- 12.6 years | 39% had absence of puberty; 35% had testicular volume <4 ml | divided into 2 groups    1. small testicular volume < 4mL n=9    2. large testicular volume >4mL n=17    Both groups were given 5,000 international units hCG IM three times per week with 150 IU hMG | 7 |
| Kliesch et al. 1994 ^69^ | Germany | Hypogonadotropic hypogonadal men (hypothalamic or pituitary (congenital or acquired)) | 26 (8 with Kallmann) | Mean ages of the men were 31 (8;21-43) years (hCG/hMG with IHH or Kallmann), 31 (7;19-39) years (GnRH) and 34 (4;26-41) years (hCG/hMG with pituitary deficiency) | Mean TV was 5.3-6.4 ml in IHH/Kallmann group, 15.5 ml in pituitary group | 1) hCG 1000-2500 IE twice a week and 75-150 IE hMG three times a week IM or SC (IHH or Kallmann)  2) Pulsatile GnRH 5-20 ug every 120 minutes (IHH or Kallmann)  3) hCG 1000-2500 IE twice a week and 75-150 IE hMG three times a week IM or SC (pituitary-deficient) | Serious |
| Kung et al. 1994 ^70^ | Hong Kong | Hypogonadotropic hypogonadism (idiopathic, Kallmann or hypopituitarism) | 17 (2 with Kallmann) | hCG median 40 years, hCG + hMG median 36 years | 47% (8/17) TV <4ml at baseline | hCG IM 2-3 times a week 1500 IU with titration to testosterone level (n=6), with addition after 6 months of 75 IU hMG (75/75 FSH/LH) IM three times a week (n=11) | 8 |
| Lin et al. 2019 ^71^ | China | Hypogonadotropic hypogonadism (congenital and acquired) | 220 (50-55% with Kallmann) | 20.4 +/- 8.4 years (GnRH) and 20.6 +/- 7.8 years (hCG/hMG) | Variable (some acquired/some CHH with absent puberty by age 18) | 5000-10000 HCG IU IM once or twice a week and 75-150 IU hMG once or twice a week with adjustment for testosterone/spermatogenesis (n=117)  GnRH 10ug every 90 minutes via SC infusion (n=103) | High |
| Liu et al. 2009 ^72^ | Australia | Gonadotropin-deficient men (pituitary, idiopathic or Kallmann) | 75 (17 with Kallmann) | 34 +/- 1 years | 58/74 (78%) had prepubertal onset | hCG 1500 or 2000 IU twice weekly, usually subcutaneous. If there was no sperm present by 6 months, FSH was added: FSH initially urinary derived 75 IU three=times-weekly, or recombinant FSH 75-150 IU three-times-weekly. | 11 |
| Liu et al. 1999 ^73^ | Australia | Hypogonadotropic hypogonadism due to pituitary or hypothalamic disease | 10 (2 Kallmann) | Median age 37 (range 26-48) years | 70% (7/10) had TV < 4ml | 2000 IU SC hCG twice weekly for 3 months, titrated if required (n=10); if poor response then after 6 months addition of recombinant FSH 150 IU SC three times a week for 18 months in addition to hCG (n=7). | 7 |
| Liu et al. 2002 ^74^ | Australia | Hypogonadotropic hypogonadism patients seeking fertility (idiopathic, Kallmann, or pituitary) | 29 (7 with Kallmann) | Median 36 years (range 26-52) | 20/29 (69%, those with IHH or Kallmann) had absent spontaneous puberty | hCG 1500 or 2000 IU twice weekly with dose titration up to four times weekly for 3-6 months, then addition of FSH if inadequate. uFSH 75 IU three times weekly or rFSH 150 IU three times weekly. Dose titration up to 150 IU FSH daily. | 9 |
| Ma et al. 2021 ^75^ | China | Male hypogonadotropic hypogonadism patients were analysed and compared between 4 subgroups: Kallman, nCHH, PSIS and acquired HH | 160 (61 with Kallmann syndrome) | Mean: 22.06 +/- 6.07 years | Patients with CHH had absence of puberty by 18 years | Group 1 regimen: hCG 5000U and hMG 150U were injected IM once a week, n = 160 Group 2 regimen: hCG 2000-3000U and hMG 75-150U were injected IM twice weekly, n = 223 | 10 |
| Mao et al. 2015 ^76^ | China | Idiopathic hypogonadotropic hypogonadism or congenital combined pituitary hormone deficiency | 75 (21 with Kallmann syndrome) | 19.4 +/- 2.3 years (IHH), 20.3 +/- 3.1 (combined pituitary deficiency) | All patients had congenital combined pituitary hormone deficiency or IHH with lack of puberty by age 18 | IM hCG 2000U twice weekly for three months followed by IM hCG 2000U and hMG 150U twice weekly for at least 24 months | 10 |
| Mastrogiacomo 1991 ^77^ | Italy | Patients with hypogonadotropic hypogonadism (pre and postpubertal onset) | 15 | Mean - 31.8 +/- 4.7 years - postpubertal, 27.5 +/- 7.3 years - prepubertal | 10/15 had prepubertal onset | hCG/FSH group: treated with 1000-2000 IU hCG + 75 IU hFSH, IM., 2-3 times per week.  LHRH: treated with LHRH 5ug/120 minutes by SC infusion. | 6 |
| Matsumoto et al. 2008 ^78^ | United States | Hypogonadotropic hypogonadism with azoospermia and mean TV < 6ml | 36 (13 with Kallmann syndrome) | 29 years (range 20-44 years) | Two patients (6%) were Tanner stage 1; all had testicular volume <6 ml | hCG 1000 U IM or SC every other day with dose titration to normalise testosterone levels before initiation of rFSH on alternate days 150-300 IU IU SC. | 9 |
| McLachlan et al 1990 ^79^ | United States | Hypogonadotropic hypogonadism (pre and post-pubertal onset) | 20 | Median 31 (20-59) years | 65% (13/20) had prepubertal onset | 2000 IU, IM, 3x/week for 6 months then add hMG 75-150 IU if insufficient response, 3x/week, for a total of 14 months. | 9 |
| Miyagawa et al. 2005 ^80^ | Japan | Hypogonadotropic hypogonadism (primary and secondary) | 36 (5 with Kallmann) | Age range 11 to 42 years | 23/36 (64%) TV <4ml | hCG 3000 IU and hMG 75 IU IM twice a week for 12-48 months, decreased after maximum testicular development to biweekly 3000 IU hCG and 150 IU hMG (if desiring to continue) or biweekly 125/250mg testosterone enanthate | 10 |
| Morris et al. 2021 ^81^ | UK | Hypogonadotropic hypogonadism (idiopathic or acquired) with azoospermia | 16 | Median 35 years | 4/12 with values available had TV <4ml | hCG 2000 IU three times a week; once testosterone sufficient, FSH 75 IU SC added three times a week, with titration up to 150IU SC three times a week (n=16) | 9 |
| Nieschlag et al. 2017 ^82^ | Australia, Germany, Italy, Poland, Spain, United Kingdom | Azoospermic men with hypogonadotropic hypogonadism (Kallmann, idiopathic, pituitary and other) | 23 | 31.5 years (20-50 years) | Not stated | Pre-treatment: hCG alone twice-weekly for 16 weeks (1500-3000 IU) to normalise testosterone or achieve spermatogenesis. Men with normalised testosterone and who were azoospermic then started 52 weeks of combined hCG and corifollitropin alfa (150 ug, once every fortnight) treatment. | 9 |
| Oguz et al. 2015 ^83^ | Turkey | Isolated hypogonadotropic hypogonadism | 44 | Mean 33.2 years (18-54) | Not stated | Group 1: n=19 patients were treated with testosterone, 6 months, exact dose not stated Group 2: n=25 patients were treated with hCG, 6 months, exact dose not stated | 8 |
| Okada et al. 1992 ^84^ | Japan | Hypogonadotropic growth-hormone deficient patients (idiopathic and acquired) | 20 | Mean 23.6 +/- 5.7 years (20-43) (males) | All had infantile external genitalia by age 15 | Group A: (n=3) no hGH and received testosterone until gonadotropin therapy was started. Dose variable, between hCG 3000 3 times per week to 5000 twice per week, and hMG 75U/week to 150 U twice per week. Group B: (n=11) testosterone during or after hGH therapy, which was then switched to gonadotropin after or during hGH therapy. Combination hGH + testosterone and/or hGH/hMG. hCG dose ranged from 1500 once/week to 5000 twice a week, hMG from 75 U once a week to 150U twice a week. Group C: (n=6) no testosterone, received gonadotropin after or during hGH therapy. Combination hGH + testosterone and/or hGH/hMG. Dose range hCG 3000 once/week to 5000 twice/week, hMG from 75 once/week to 75 twice/week. | 8 |
| Oldereid et al. 2010 ^85^ | Norway | Hypogonadotropic hypogonadal men referred for induction of spermatogenesis (idiopathic, syndromic and acquired) | 17 (1 Kallmann) | / | 11/13 who completed treatment had not undergone spontaneous puberty | Urinary hCG SC 2500-5000 IU twice-three times a week for 3 months, titrated to testosterone; if no spermatogenesis then hMG or recombinant FSH (rFSH) 75-150 IU three times a week were added (up to 225 IU when unresponsive) (n=17) | 8 |
| Raivio et al. 2007 ^86^ | Finland | Boys with prepubertal onset of hypogonadotropic hypogonadism (idiopathic, Kallmann, pituitary) | 14 (2 Kallmann) | Aged 9.9-17.7 years | All had prepubertal onset with TV <3ml | Pretreatment with rhFSH three times a week SC 60-150 IU/dose for 2 months-2.8 years, with pubertal induction by hCG dose between 500 IU per 2 weeks to 4000 IU per week, 1-3 times per week SC) (n=14) | 8 |
| Rehman et al. 2014 ^87^ | Pakistan | Hypogonadotropic hypogonadism (mixed aetiologies) | 26 | 18-43 (29.38 +/- 7.94) years | Not stated | 1500 U hCG and 75 U hMG on alternate days, with addition of rFSH follitropin alpha 150 U on alternate days for non-responders to hMG (n=26) | 10 |
| Resorlu et al. 2009 ^88^ | Turkey | Idiopathic hypogonadotropic hypogonadism  or secondary hypogonadotropic hypogonadism | 17 | 25-38 years | 11/17 had idiopathic prepubertal HH | Group 1 n=11, men with idiopathic HH, intramuscular doses of 5000 IU hCG applied three times a week. From the third month onward, 75 IU recombinant human FSH subcutaneous daily dose was added to hCG treatment. Treatment continued until pregnancy occurred.  Group 2: n=6, men with secondary HH, intramuscular doses of 5000 IU hCG applied two times a week. From the third month onward, 75 IU recombinant human FSH subcutaneous daily dose was added to hCG treatment. Treatment continued until pregnancy occurred. | 8 |
| Rohayem et al. 2016 ^89^ | Germany | Hypogonadotropic Hypogonadism (Kallmann, congenital, MPHD) | 51 (11 Kallmann) | 22-47 years | 41/51 had either absent puberty or pubertal arrest | 1500 IU hCG twice a week, followed by combined hCG/hMG or recombinant FSH (rFSH) 3 x 150 IU SC/week after 3 months with dose titration to testosterone levels. | 10 |
| Rohayem et al. 2017 ^90^ | Germany | Hypogonadotropic hypogonadism (Kallmann, congenital, MPHD, CHARGE) | 60 (20 Kallmann) | Mean age 15.5 (prepubertal) and mean 18.8 years (fully virilised) | 34/60 (56.7%) were prepubertal or had early arrested puberty with lack of virilisation by testosterone | 250-500 IU hCG SC injection twice a week, incremental increases every 6 months to a maximum of 3x2500 IU hCG per week. rFSH (follitropin alpha) 3x(75-)150 IU SC/week were added when adequate testosterone reached (n=34)  In the testosterone virilised group, hCG 1500 IU SC twice a week, with increase after 6-9 months up to 3x2500 IU hCG SC/week, with addition of rFSH (follitropin alpha) 150 IU three times a week after 3 months. (n=26) | 9 |
| Sato et al. 2015 ^91^ | Japan | Hypogonadotropic hypogonadism (Kallmann, isolated, pituitary, CHARGE) | 55 (7 Kallmann) | Mean age 15.7 years | Not stated | hCG monotherapy and hCG-rFSH combination therapy; hCG dose ranging from 500 U twice a week to 4000 U three times a week; then rFSH dose ranging from 75 U twice a week/150 U once a week to 225 U three times a week; others had testosterone monotherapy. n=17 received hCG-rFSH and underwent semen testing. | 8 |
| Shankar et al. 2022 ^92^ | United States | Adolescent boys with hypogonadotropic hypogonadism (congenital or prepubertally acquired) | 17 (14 Kallmann) | Mean 15.5 +/- 0.9 years | 16/17 (94.1%) genital Tanner stage 1 | Corifollitropin alfa (FSH agonist) priming 100-150ug SC once every two weeks; at week 12, they entered combined treatment of CFA with hCG SC twice a week between 500-5000 IU titrated to testosterone and oestrogen levels; total duration 64 weeks (n=17) | 10 |
| Shiraishi et al. 2014 ^93^ | Japan | Male hypogonadotropic hypogonadism (primary and secondary) | 37 (3 Kallmann) | Mean 26.8 +/- 8.5 years (hCG/FSH group) | 49% had absence of puberty | hCG SC for 6 months, followed by 75 IU rhFSH three times per week for 2 years (n=31) or Testosterone enanthate 250mg IM injection every 3 weeks (n=6) | 9 |
| Shiraishi et al. 2021 ^94^ | Japan | Hypogonadotropic hypogonadism (primary and secondary) | 26 | hCG mean 34.2 ± 5.2 years, TRT mean 34.7 ± 4.1 years | Not stated | Continue testosterone replacement IM 250mg testosterone enanthate every 3 weeks (all patients) then to continue this treatment (n=10) or hCG 3000 IU twice a week titrated to testosterone/side effects (n=16) | Moderate |
| Sinisi et al. 2008 ^95^ | Italy | Males with never-treated prepubertal-onset hypogonadotropic hypogonadism | 20 (5 Kallmann) | 20.4 +/- 2.1 years | Tanner Stage 1-2 for all | Pre-treatment: IM hCG 2000 IU twice weekly to normalise testosterone and induce puberty.   Group 1: Continued hCG alone 2000 IU IM twice weekly  Group 2: hCG 2000 IU IM twice weekly plus rFSH (75IU) twice weekly | 9 |
| Sinisi et al. 2010 ^96^ | Italy | Hypogonadotropic hypogonadal men with failed induction of spermatogenesis on hCG alone | 34 (6 Kallmann) | Age range between 22.7 +/- 1.5 to 24.9 +/- 1.7 years across four groups | All had prepubertal onset. 11/34 (32.4%) had TV <4ml. | All patients received 2000 IU hCG twice a week for several months before introducing rFSH, with arms: 1) rFSH 75 IU twice a week (n=10)  2) rFSH 75 IU three times a week (n=10)  3) hp-FSH 75 IU twice a week (n=7)  4) hp-FSH 75 IU three times a week (n=7) | Moderate |
| Trinh et al. 2021 ^97^ | Vietnam | Male hypogonadotropic hypogonadism | 19 (5 Kallmann) | Mean 30.2 +/- 5.6 years | 16/19 had total absence of puberty, Tanner Stage 1/2 | hCG administered every 3 days (dose ranging 3000-10,000 IU) in combination with clomiphene citrate at 25mg per day. Supplementation with FSH or hMG 75IU was added every 3 days for spermatogenesis if the patient wanted to have children. Dose was maintained until spermatozoa appeared in the semen. Average hCG dose was 5000 IU. | 9 |
| Unluhizarci et al. 2020 ^98^ | Turkey | Hypogonadotropic hypogonadism | 13 | Median 26 years (18.8–33.5) | Not stated | 1,500 U hCG IM, three times a week plus 75 U of follitropin alpha SC three times a week | 8 |
| Wang et al. 2014 ^99^ | China | Male patients with hypogonadotropic hypogonadism resulting from pituitary stalk interruption or other causes | 38 | Adolescents - median 15.5 (13.8-16.4) years  Young adults - median 21.2 (20.1-24) years | Groups 1 and 3 (n=38) had median Tanner stage of 1 | Pre-treatment: hCG (2000 IU) twice-weekly for 3 months Treatment phase: combined hCG (2000 IU) and hMG (75IU) twice-weekly for 6 months | 10 |
| Warne et al. 2009 ^100^ | Japan, Europe, Australia and United States | Men with idiopathic or acquired hypogonadotropic hypogonadism | 100 (29 Kallmann) | Europe (n=32) = mean 25.9 (7.7) Australia (n=10) = 36.0 (7.0) United States (n=36) = 29.8 (5.0) Japan (n=22) = 31.2 (8.8) | 9/100 were Tanner Stage I at baseline | Pre-treatment: hCG to normalise serum T. Starting dose of 1000 IU three-times-weekly or 2000 IU twice-weekly for 3-6 months. Treatment phase: hCG continued at the same dose and combined with r-hFSH (150 IU) three-times-weekly for up to 18 months. | 9 |
| Yang et al. 2012 ^101^ | China | Idiopathic hypogonadotropic hypogonadism or delayed puberty | 242 (2 Kallmann) | Mean: 23.1 +/- 6.2 years age range: 15-52 years | 90.5% had testicular volume <4ml | Group 1: IM hCG 2,000 IU, every 3 days, n=84 Group 2: Combination of hCG/hMG (2,000 IU/ 75 IU, every 3 days) n=74 Group 3: n=84, Oral testosterone undecanoate, 120-160 mg per day for 2-3 weeks and then dosage adjusted to 40-120 mg per day for maintenance | Serious |
| Young et al. 1999 ^102^ | France | Men with acquired/congenital HH or eugonadic patients with prostate cancer | 40 (6 Kallmann) | CHH - 24 +/- 5 years Acquired HH - 40 +/- 10 years Eugonadic post treatment - 66 +/- 6 years | 12/40 had CHH with absent spontaneous puberty | Group 1 (n=10): hCG therapy, 1500 IU, IM., twice-weekly for 6 months Group 2 (n=8): testosterone treatment, 250mg IM., 3-week-intervals | 10 |
| Zacharin et al. 2012 ^103^ | Australia and India | Males with hypogonadotropic hypogonadism (idiopathic, congenital and acquired) | 19 (5 Kallmann) | Median - 18.9 years (14.5-31.0) | 100% of patients were prepubertal | Group 1 (n=9): hCG alone, initially 500 IU twice-weekly with increases to 1000 IU at 6 months and as puberty progressed, to 1500 IU twice-weekly Group 2 (n=10): 500-1500 IU hCG twice-weekly, with addition of 150-300 IU recombinant FSH three-times-weekly | Serious |

| **Supplementary Table 3 – Quality appraisal of randomised or non-randomised comparative studies** | | | | | | | | | | |
| --- | --- | --- | --- | --- | --- | --- | --- | --- | --- | --- |
| **Randomised controlled trials** | | | | | | | | | | |
| **Citation** | **Title** | **Risk of bias arising from the randomization process** | **Risk of bias due to deviations from the intended interventions (effect of assignment to intervention)** | **Risk of bias due to deviations from the intended interventions (effect of adhering to intervention)** | | **Missing outcome data** | | **Risk of bias in measurement of the outcome** | **Risk of bias in selection of the reported result** | **OVERALL CONSENSUS** |
| Bouloux et al. 2003 ^53^ | Induction of Spermatogenesis by Recombinant Follicle-Stimulating Hormone (Puregon) in Hypogonadotropic Azoospermic Men Who Failed to Respond to Human Chorionic Gonadotropin Alone | Some concerns | Low | Low | | Some concerns | | Low | Some concerns | Some concerns |
| Dwyer et al. 2013 ^11^ | Trial of recombinant follicle-stimulating hormone pretreatment for GnRH-induced fertility in patients with congenital hypogonadotropic hypogonadism | Low | Low | Some concerns | | Some concerns | | Low | Low | Some concerns |
| Lin et al. 2019 ^71^ | Optimal treatment for spermatogenesis in male patients with hypogonadotropic hypogonadism | High | Low | Low | | Some concerns | | Low | High | High |
| Liu et al. 2017 ^25^ | The effectiveness of zinc supplementation in men with isolated hypogonadotropic hypogonadism | Low | Low | Some concerns | | Some concerns | | Low | Low | Some concerns |
| Schaison et al. 1993 ^39^ | Failure of Combined Follicle-Stimulating Hormone-Testosterone Administration to Initiate and/or Maintain Spermatogenesis in Men with Hypogonadotropic Hypogonadism | Some concerns | Low | Some concerns | | Low | | Low | Some concerns | Some concerns |
| Zhang et al. 2015 ^49^ | Sequential versus Continual Purified Urinary FSH /hCG in Men with Idiopathic Hypogonadotropic Hypogonadism | Low | Low | Low | | Low | | Low | Low | Low |
| **Non-randomised studies of interventions** | | | | | | | | | | |
| **Citation** | **Title** | **Bias due to confounding** | **Bias in selection of participants into the study** | **Bias in classification of interventions** | **Bias due to deviations from intended interventions** | | **Bias due to missing data** | **Bias in measurement of outcomes** | **Bias in selection of the reported result** | **OVERALL CONSENSUS** |
| Aydogdu et al. 2013 ^2^ | Effects of three different medications on metabolic parameters and testicular volume in patients with hypogonadotropic hypogonadism: 3-year experience | Moderate | Low | Low | Low | | Low | Low | Moderate | Moderate |
| Buchter et al. 1998 ^55^ | Pulsatile GnRH or human chorionic gonadotropin/human menopausal gonadotropin as effective treatment for men with hypogonadotropic hypogonadism: A review of 42 cases | Serious | Moderate | Moderate | Moderate | | Low | Low | Moderate | Serious |
| Celik et al. 2021 ^57^ | Role of testosterone to estradiol ratio in predicting the efficacy of recombinant human chorionic gonadotropin and testosterone treatment in male hypogonadism | Serious | Low | Moderate | Low | | Low | Low | Low | Serious |
| Chen et al. 2019 ^7^ | Testosterone undecanoate supplementation together with human chorionic gonadotropin does not impair spermatogenesis in males with isolated hypogonadotropic hypogonadism: A retrospective study | Moderate | Low | Moderate | Low | | Low | Low | Low | Moderate |
| Gong et al. 2015 ^16^ | Pulsatile GnRH is superior to HCG in therapeutic efficacy in adolescent boys with hypogonadotropic hypogonadodism | Moderate | Low | Low | Low | | Low | Low | Moderate | Moderate |
| Kliesch et al. 1994 ^69^ | High efficacy of gonadotropin or pulsatile gonadotropin-releasing hormone treatment in hypogonadotropic hypogonadal men | Serious | Moderate | Low | Low | | Low | Low | Moderate | Serious |
| Liu et al. 2021 ^26^ | Efficacy and safety of human chorionic gonadotropin combined with human menopausal gonadotropin and a gonadotropin-releasing hormone pump for male adolescents with congenital hypogonadotropic hypogonadism | Moderate | Low | Low | Low | | Low | Low | Moderate | Moderate |
| Mao et al. 2017 ^27^ | Pulsatile gonadotropin-releasing hormone therapy is associated with earlier spermatogenesis compared to combined gonadotropin therapy in patients with congenital hypogonadotropic hypogonadism | Serious | Moderate | Low | Low | | Moderate | Low | Low | Serious |
| Ortac et al. 2020 ^33^ | Efficacy of follitropin-alpha versus human menopausal gonadotropin for male patients with congenital hypogonadotropic hypogonadism | Moderate | Moderate | Low | Low | | Low | Low | Moderate | Moderate |
| Sanyal and Chatterjee 2016 ^38^ | Treatment preferences and outcome in male hypogonadotropic hypogonadism: an Indian perspective | Critical | Low | Moderate | Low | | Low | Low | Moderate | Critical |
| Schopohl et al. 1991 ^41^ | Comparison of gonadotropin-releasing hormone and gonadotropin therapy in male patients with idiopathic hypothalamic hypogonadism | Moderate | Moderate | Low | Moderate | | Moderate | Low | Moderate | Moderate |
| Schopohl et al. 1993 ^40^ | Pulsatile gonadotrophin releasing hormone versus gonadotrophin treatment of hypothalamic hypogonadisim males | Moderate | Moderate | Low | Moderate | | Moderate | Low | Moderate | Moderate |
| Shiraishi et al. 2021 ^94^ | Patient-reported outcomes and biochemical alterations during hormonal therapy in men with hypogonadotropic hypogonadism who have finished infertility treatment | Moderate | Low | Low | Low | | Low | Low | Moderate | Moderate |
| Sinisi et al. 2010 ^96^ | Efficacy of recombinant human follicle stimulating hormone at low doses in inducing spermatogenesis and fertility in hypogonadotropic hypogonadism | Moderate | Moderate | Moderate | Low | | Low | Low | Low | Moderate |
| Trabado et al. 2011 ^44^ | Estradiol levels in men with congenital hypogonadotropic hypogonadism and the effects of different modalities of hormonal treatment | Serious | Low | Low | Low | | Low | Low | Moderate | Serious |
| Yang et al. 2012 ^101^ | Application of hormonal treatment in hypogonadotropic hypogonadism: more than ten years experience | Serious | Low | Moderate | Low | | Low | Low | Moderate | Serious |
| Zacharin et al. 2012 ^103^ | Addition of recombinant follicle-stimulating hormone to human chorionic gonadotropin treatment in adolescents and young adults with hypogonadotropic hypogonadism promotes normal testicular growth and may promote early spermatogenesis | Serious | Serious | Low | Low | | Low | Low | Moderate | Serious |
| Zhang et al. 2019 ^50^ | The Pulsatile Gonadorelin Pump Induces Earlier Spermatogenesis Than Cyclical Gonadotropin Therapy in Congenital Hypogonadotropic Hypogonadism Men | Serious | Serious | Low | Low | | Moderate | Low | Low | Serious |
| Zhao et al. 2013 ^51^ | A Network Investigation on Idiopathic Hypogonadotropic Hypogonadism in China | Moderate | Moderate | Serious | Serious | | Low | Low | Moderate | Serious |

| **Supplementary Table 4: Key studies investigating change in testicular volume and spermatogenesis in cohorts where the majority of patients lacked spontaneous puberty** | | | | | | | | | | | |
| --- | --- | --- | --- | --- | --- | --- | --- | --- | --- | --- | --- |
| **Citation** | **Population** | **Sample size in treatment arm** | **Age** | **Pubertal status** | **Pre-treatment** | **Treatment** | **Pre-treatment testicular volumes**  **(Ultrasound unless stated)** | **Post-treatment testicular volumes** | **Significance** | **Mean change** | **Spermatogenesis** |
| **hCG only treatment** | | | | | | | | | | | |
| Aydogdu et al. 2013 ^2^ | Never-treated men with idiopathic hypogonadotropic hypogonadism | 25 | Mean 21.4 +/- 1.3 years | Mean Tanner stage 1.3 +/- 0.5 | None | hCG 5000 IU twice weekly for 6 months | Mean 1.7 +/- 1.5 ml  via ultrasound | Mean 2.2 +/- 1.6 ml | p=0.0001 | 0.5 ml | / |
| Bayram et al. 2015 ^3^ | Men with idiopathic hypogonadotropic hypogonadism | 16 | Mean 27.5 +/- 10.5 years | 100% prepubertal onset | One patient had been previously treated; no medications permitted for 6 months before | hCG 1500 IU three times a week for 6 months | Mean 3.6 +/- 0.7 ml via Prader orchidometer | Mean 9.1 +/- 3.4 ml | p=0.0001 | 5.5 ml | / |
| Bouvattier 1999 ^54^ | Hypogonadal males with wish to increase their testicular volume (combined aetiologies) | 37 | Mean 16.4 +/- 1.8 years | 89% had initial TV <4ml | None | hCG 1500 IU twice a week for 6 months | Mean 1.98 +/- 1.2 ml | Mean 6.5 +/- 1 ml | p<0.0001 | 4.52 ml | / |
| Gong et al. 2015 ^16^ | Peri-pubertal boys with hypogonadotropic hypogonadism, either Kallmann or normosmic idiopathic | 22 | Mean 13.89 +/- 2.44 years | >80% Tanner Stage I at baseline | Not stated | hCG 1000-2000 IU two to three times a week for 12 months | Mean 2.15 +/- 2.05 ml via Prader orchidometer | Mean 6.01 +/- 1.33 ml | Not significant | 3.9 ml | / |
| Kirk et al. 1994 ^21^ | Male patients with idiopathic hypogonadotropic hypogonadism | 26 | Median 21.0 (range 18-48) years in those with cryptorchidism, 19.0 (16-27) in those without | Mean TV <3ml for all patients | In cryptorchid group, 10 had been treated with testosterone and 4 with hCG, 2 with GnRH; 3 in non-cryptorchid group had been treated with testosterone and 1 with hCG | hCG 2000 IU twice weekly for 6 weeks, then 1000 IU twice weekly | Mean 1.3 +/- 0.5ml in those with cryptorchidism, 1.8 +/- 0.8ml in those without via Prader orchidometer | Mean 3.0 +/- 1.6ml in those with cryptorchidism, 4.7 +/- 1.8ml in those without | Not stated | / | / |
| Sinisi et al. 2008 ^95^ | Men with never-treated prepubertal onset hypogonadotropic hypogonadism | 9 | Mean 19.4 +/- 1.87 years | Tanner stage 1-2 for all | None | hCG 2000 IU twice weekly for 6-12 months | Mean 3.33 +/- 1.0 ml via orchidometer | Mean 4.6 +/- 1.22 ml | Not stated | 1.27 ml | 2/9 (22.2%) |
| Vicari et al. 1992 ^45^ | Males with isolated/congenital hypogonadotropic hypogonadism – small testis subset | 8 | Mean 21.8 +/- 1.4 for whole cohort | All had failure of puberty by age 18; all in small testis subset had baseline TV <4ml | Four had no pre-treatment, others had pre-treatment with hCG or testosterone | hCG 1500 IU three times a week for a mean of 19 months | Mean 3 +/- 0.1 ml via orchidometer | Mean 10.7 +/- 0.5 ml after 24 months | p<0.05 | 7.7 ml | 5/8 (62.5%) |
| Yang et al. 2012 ^101^ | Males with idiopathic hypogonadotropic hypogonadism | 84 | Mean 18.8 +/- 2.5 years | 90.5% of whole cohort had TV <4 ml | Not stated | hCG 2000 IU every 3 days for 6-18 months | Mean 2.0 +/- 1.1 ml via Prader orchidometer | Mean 6.8 +/- 3.2 ml  Note: non-responders excluded | p=0.000 | 4.8 ml | 34/84 (40.5%) |
| Zacharin et al. 2012 ^103^ | Adolescent and young adult men with hypogonadotropic hypogonadism (idiopathic, congenital and acquired) | 9 | Median 18.1 (range 14.5-31.0) years | 100% prepubertal at diagnosis | 6 were treatment naïve, 3 previously testosterone-treated | hCG 500-1500 IU twice weekly for 9 months | Median combined TV 4ml (range 2-8 ml) via Prader orchidometer | Median combined TV 11ml (range 3-30 ml) | Not stated | / | 3/9 (33.3%) |
| **hCG + FSH** | | | | | | | | | | | |
| Barrio et al. 1999 ^52^ | Prepubertal adolescent males with hypogonadotropic hypogonadism | 14 | Mean 15.44 +/- 1.97 years (Isolated) and 18.1 +/- 3.24 (panhypopituitarism) | 100% prepubertal | Five patients had received testosterone therapy | hCG 1000-1500 IU and FSH 75-100 IU every two days until complete virilisation achieved | Mean 1.9 +/- 0.9 ml (isolated) and mean 2.6 +/- 1.7 ml (panhypopituitarism) via Prader orchidometer | Mean 10.4 +/- 3.9ml and mean 15.3 +/- 4.9 ml | p<0.05 (both) | 8.5 ml and 12.7 ml | 7/8 (87.5%) |
| Bouloux et al. 2002 ^5^ | Azoospermic men with hypogonadotropic hypogonadism – a combination of idiopathic, Kallmann, and other causes | 19 | Mean 25.9 +/- 7.7 years | All patients had mean TV <4 ml | Patients were excluded if they had testosterone in past 5 weeks and hCG in past 2 weeks | hCG 2000 IU twice weekly (with dose titration) then 18 months of hCG plus rFSH (follitropin alpha) 150-225 IU three times a week | Mean 2.5 +/- 1.0 ml via Prader orchidometer | Mean 12.2 +/- 4.9 ml | Not stated | 9.7 ml | 15/19 (78.9%) |
| Burgues et al. 1997 ^56^ | Azoospermic men with hypogonadotropic hypogonadism (combination of idiopathic, Kallmann, and hypopituitarism) | 60 | Mean 26.3 +/- 0.85 years | 86.7% prepubertal onset | 95% pre-treated (either gonadotropins or testosterone) | hCG 2500 IU twice weekly and FSH-HP 150 IU three times a week for minimum 6 months | Mean 4.3 +/- 0.5 ml via caliper measurements | Mean 11.1 +/- 1.0 ml | p<0.001 | 6.8 ml | 48/60 (80.0%) |
| Cangiano et al. 2021 ^6^ | Males with congenital hypogonadotropic hypogonadism | 19 | Mean 17.4 +/- 2.7 years | Absent puberty (bilateral TV <8ml) 84% | Pre-treatment with FSH (68%) and testosterone (37%) | Majority started hCG 250 IU 2-3/week and uFSH 75 IU three times a week; with uptitration to hCG 1000 IU twice weekly | Mean bilateral testicular volume 5.3 +/- 2.7 ml via Prader orchidometer | Median bilateral TV 23 ml (IQR 14-30 ml) | Not stated | / | 16/16 (100.0%) although five had low concentrations <0.1x10^6^/mL |
| Liu et al. 2017 | Men with isolated hypogonadotropic hypogonadism | 34 | Mean 24.1 +/- 4.9 years | 100% without spontaneous puberty | No prior treatment with GnRH or FSH | hCG 2000 IU twice a week for 18 months and 75 IU uFSH three times a week every other 3 months for 6-18 months | Median 1.5ml (IQR 1.0-3.0) via ultrasound | Median 5.3 ml (IQR 3.2-7.0) | p<0.001 | / | 22/34 (64.7%) |
| Raivio et al. 2007 ^86^ | Boys with prepubertal onset of hypogonadotropic hypogonadism (idiopathic, Kallmann, pituitary) | 14 | Aged 9.9-17.7 years | 100% with prepubertal onset and TV <3ml | Un-virilised and pre-pubertal; presumed untreated | rFSH pretreatment 180-450 IU/week for 2 months-2.8 years then hCG 500-4000 IU per week, 1-3/week | Mean 0.9 +/- 0.7 ml via ruler measurement | Median approximately 5-6ml (graphically visualised) | p<0.001 | / | 6/7 (85.7%) |
| Resorlu et al. 2009 ^88^ | Idiopathic prepubertal men with hypogonadotropic hypogonadism | 11 | Mean 30.1 years (range 26-38) | 100% prepubertal onset | Seven patients had received prior testosterone therapy | hCG 5000 IU three times a week and rFSH 75 IU from the third month for 18-24 months | Mean 5.7/5.9 ml (right/left) (range 2-9) via various methods | Mean 7.5/7.8 ml (right/left) (range 4-12) | Not stated | / | / |
| Rohayem et al. 2017 ^90^ | Congenital hypogonadotropic hypogonadism with absent puberty | 18 | Not stated for subgroup; mean 15.5-18.8 depending on if pre-treated for full cohort | 100% absent spontaneous puberty | 8/18 pre-virilised with testosterone | hCG 250-500 IU twice weekly to 2500 IU three times a week for 6 months-1 year, then rFSH 75-150 IU three times a week | Mean bilateral TV 3.5 +/- 1.7 (no pre-treatment); 4.6 +/- 2.4 ml (pre-treated) via Prader orchidometer | Mean bilateral TV 39 +/- 18 ml (no pre-treatment); 36 +/- 16 (pre-treated) | Not stated | / | Within whole cohort including other aetiologies, sperm were found in 21/23 (91%) of no pre-treatment group, 18/19 (95%) of pre-treated group |
| Shankar et al. 2022 ^92^ | Adolescent boys with hypogonadotropic hypogonadism (congenital or prepubertally acquired) | 17 | Mean 15.5 +/- 0.9 years | 94.1% genital Tanner stage 1 | Patients were excluded if any previous treatment | Corifollitropin alfa (CFA), a long-acting FSH agonist, 100-150 ug every 2 weeks for 12 weeks, then 52 weeks CFA combined with hCG 500-5000 IU twice weekly | Mean 2.2 +/- 1.9 ml via ultrasound | Mean 12.9 ml | Geometric mean fold increase from baseline was 9.43 (95% CI, 7.44-11.97) | 10.7 ml | / |
| Sinisi et al. 2010 ^96^ | Men with prepubertal onset hypogonadotropic hypogonadism | 14 | Mean 24.5 +/- 8.5 (2/week group) and 24.4 +/- 2.6 (3/week group) | 100% prepubertal onset; 4/17 TV <4ml at start | 3 previously treated with androgens, 2 with hCG | hCG 2000 IU twice a week for several months then hpFSH 75-150 IU 2-3/week for 12 months | Median 3.5ml (range 2-7) via Prader orchidometer | Median 8.0 (range 4-15ml) | Not stated | / | Absolute rate not described |
| Zacharin et al. 2012 ^103^ | Adolescent and young adult men with hypogonadotropic hypogonadism (idiopathic, congenital and acquired) | 10 | Median 20.9 (range 16-25) years | 100% prepubertal at diagnosis | 5 were treatment naïve, 5 previously testosterone-treated | hCG 500-1500 IU twice weekly and rFSH 150-300 IU three times a week from four months after dose increase of hCG, for 9 months | Median combined TV 4ml (range 2-8 ml) via Prader orchidometer | Median combined TV 19ml (range 8-50 ml) | Not stated | / | 10/10 (100%) |
| * Contains studies where 75%+ of the population were described as prepubertal or had prepubertal onset of disease, or average TV <4ml or Tanner < Stage 1.5 | | | | | | | | | | | |

| **Supplementary Table 5: Key studies investigating change in penile length in cohorts where the majority of patients lacked spontaneous puberty** | | | | | | | | | | |
| --- | --- | --- | --- | --- | --- | --- | --- | --- | --- | --- |
| **Citation** | **Population** | **Sample size in treatment arm** | **Age** | **Pubertal status** | **Pre-treatment** | **Treatment** | **Pre-treatment penile length** | **Post-treatment penile length** | **Significance** | **Mean change** |
| **hCG treatment** | | | | | | | | | | |
| Bayram et al. 2015 ^3^ | Men with idiopathic hypogonadotropic hypogonadism | 16 | Mean 27.5 +/- 10.5 years | 100% prepubertal onset | One patient had been previously treated | hCG 1500 IU three times a week for 6 months | Mean 3.6 +/- 0.7 cm | Mean 7.9 +/- 1.5 cm | p=0.0001 | 4.3 cm |
| Gong et al. 2015 ^16^ | Adolescent boys with hypogonadotropic hypogonadism, either Kallmann or normosmic idiopathic | 22 | Mean 13.89 +/- 2.44 years | >80% Tanner Stage I at baseline | Not stated | hCG 1000-2000 IU two to three times a week for 12 months | Mean 3.91 +/- 1.38 cm | Mean 6.03 +/- 1.10 cm | p<0.05 | 2.12 cm |
| **hCG + FSH treatment** | | | | | | | | | | |
| Barrio et al. 1999 ^52^ | Prepubertal adolescent males with hypogonadotropic hypogonadisms | 14 | Mean 15.44 +/- 1.97 years (Isolated) and 18.1 +/- 3.24 (panhypopituitarism) | 100% prepubertal | Five patients had received testosterone therapy | hCG 1000-1500 IU and FSH 75-100 IU every two days until complete virilisation achieved | Isolated HH – Mean 4.8 +/- 0.4 cm; Panhypopituitarism – Mean 6.3 +/- 1.8 cm | Isolated HH – Mean 8.9 +/- 0.8 cm; Panhypopituitarism – Mean 9.2 +/- 0.6 cm | p<0.05 for both | 4.1 cm and 2.9 cm |
| Burgues et al. 1997 ^56^ | Azoospermic men with hypogonadotropic hypogonadism (combination of idiopathic, Kallmann, and hypopituitarism) | 60 | Mean 26.3 +/- 0.85 years | 86.7% prepubertal onset | 95% pre-treated (either gonadotropins or testosterone) | hCG 2500 IU twice weekly and FSH-HP 150 IU three times a week for minimum 6 months | Mean 6.0 +/- 0.3 cm | Mean 8.1 +/- 0.3 cm | p<0.001 | 2.1 cm |
| Liu et al. 2017 ^25^ | Men with isolated hypogonadotropic hypogonadism | 34 | Mean 24.1 +/- 4.9 years | 100% without spontaneous puberty | No prior treatment with GnRH or FSH | hCG 2000 IU twice a week for 18 months and 75 IU uFSH three times a week every other 3 months for 6-18 months | Mean 4.3 +/- 1.0 cm | Mean 6.9 +/- 1.3 cm | p=0.001 | 2.6 cm |
| * Contains studies where 75%+ of the population were described as prepubertal or had prepubertal onset of disease, or average TV <4ml or Tanner < Stage 1.5 | | | | | | | | | | |

| **Supplementary Table 6: Summary of all papers with complete pre-pubertal cohorts of CHH/IHH (or subgroups) reporting no previous treatment with testosterone (or subgroups who had never been treated)** | | | | | | | | | | |
| --- | --- | --- | --- | --- | --- | --- | --- | --- | --- | --- |
| **Citation** | **Population** | **Sample size** | **Age** | **Pubertal status** | **Androgen exposure status** | **Gonadotropin regime** | **Testicular volume (TV) change** | **Penile length change** | **Spermatogenesis** | **Biochemical parameters** |
| Aydogdu et al. 2013 ^2^ | Never-treated patients with idiopathic hypogonadotropic hypogonadism | 77 | Mean 21 ± 1.4 years | Mean Tanner stage 1.3 +/- 0.5 | 0% previously treated | Three treatment groups - one of hCG 5000 IU twice weekly, one of IM testosterone (Sustanon Ⓡ 250mg) once every 3 weeks, one of daily testosterone gel (Testogel Ⓡ 250mg/5g), all for 6 months | Baseline TV 1.7 ± 1.5 ml, post-treatment 2.2 ± 1.6 ml, p=0.0001 | / | / | Testosterone baseline 36.4 ± 51.1 ng/dl, post-treatment 52.7 ± 45.4 ng/dl, p=0.115 |
| Cangiano et al.. 2021 ^6^ | Congenital hypogonadotropic hypogonadism | 19 | Aged 14-23 | 15/19 Bilateral TV <8ml = 78.9% | 12/19 previously untreated with testosterone | FSH 75 IU thrice/week and hCG 250 IU 2 or 3 times/week for most patients. Thirteen patients underwent pretreatment of 75 IU FSH three/week for 4 months before combination. | Among 12 previously untreated patients, median baseline bilateral TV was 5ml, and 20.5ml at 24 months post-treatment. There was no significant difference according to pre-treatment status. | / | 12/12 previously untreated patients achieved spermatogenesis, although 3/12 had low concentrations <0.1x10^6^/mL | / |
| Fan et al. 2022 ^14^ | Congenital hypogonadotropic hypogonadism | 52 | Mean 20.7 ± 5.3 years | 100% no spontaneous puberty by age 15 and Tanner stage 1-2 | 0% previously treated | SC pulsatile GnRH via infusion pump, 10 ug every 90 minutes for median 13.5 (11.3-24 months) | Baseline TV 2.0 (1.4-2.6) ml, post-treatment 7.8 (3.8-12.6) ml, p<0.0001 | / | / | Testosterone baseline 0.9 ± 1.2 nmol/L, post-treatment 7.3 ± 4.6 nmol/L, p<0.0001 |
| Kilciler et al. 2002 ^20^ | Idiopathic hypogonadotropic hypogonadism | 14 | Mean 21.1 ± 1.2 years | 100% failed to have spontaneous puberty by age 18 years | 0% previously treated with testosterone due to exclusion criteria | hCG 5000 IU three times a week IM + hMG (Pergonal, 75 IU FSH plus 75 IU LH) | / | / | / | Total Testosterone baseline 6.07 ± 1.87 nM/L, post-treatment 39.07 ± 3.82 nM/L, p=0.001 |
| Mao et al. 2015 ^76^ | Idiopathic hypogonadotropic hypogonadism or congenital combined pituitary hormone deficiency | 75 (53 IHH) | 19.4 +/- 2.3 years (IHH) | Subgroup with IHH and lack of puberty by age 18 | 21 out of 53 had not been exposed to testosterone therapy previously | IM hCG 2000U twice weekly for three months followed by IM hCG 2000U and hMG 150U twice weekly for at least 24 months | / | / | The success rate of spermatogenesis was 85.7% (18/21) in those IHH patients who had not received prior testosterone therapy, compared to 84% (27/32) in those who had, p=0.694 | / |
| Oktenli et al. 2003 ^32^  Similar cohort and therapy:  Musabak et al. 2003 ^28^  and  Yesilova et al. 2000 ^46^ | Idiopathic hypogonadotropic hypogonadism | 35 | Mean 21.34 ± 1.53 years | 100% failed to have spontaneous puberty by age 18 | Patients were newly diagnosed and hence presumed untreated | hCG 2000 IU and hMG (75 IU FSH + 75 IU LH) three times a week for 6 months | / | / | / | Free testosterone baseline 1.51 ± 0.66 pg/ml, post-treatment 18.18 ± 1.59 pg/ml, p<0.001 |
| Raivio et al. 2007 ^86^ | Boys with prepubertal onset of hypogonadotropic hypogonadism (idiopathic, Kallmann, pituitary) | 14 (4 either IHH or Kallmann) | Aged 9.9-17.7 years | All had prepubertal onset with TV <3ml | Un-virilised and pre-pubertal; presumed untreated | Pretreatment with rhFSH three times a week SC 60-150 IU/dose for 2 months-2.8 years, with pubertal induction by hCG dose between 500 IU per 2 weeks to 4000 IU per week, 1-3 times per week SC) | / | / | 2/2 IHH patients achieved spermatogenesis, 1/2 Kallmann syndrome patients achieved spermatogenesis | / |
| Rohayem et al. 2017 ^90^ | Hypogonadotropic hypogonadism (Kallmann, congenital, MPHD, CHARGE) | 60 (11 Kallmann and 10 CHH with absent puberty) | Prepubertal Group A had mean age 15.5 years | Group A subgroup were prepubertal or had early arrested puberty. | Prepubertal Group A were previously untreated with testosterone | 250-500 IU hCG SC injection twice a week, incremental increases every 6 months to a maximum of 3x2500 IU hCG per week. rFSH (follitropin alpha) 3x(75-150 IU) SC/week were added when adequate testosterone reached | In prepubertal Group A Kallmann: baseline bi-testicular volume 1.5 ± 0.6 ml, post-treatment 16 ± 8 ml; in CHH absent puberty baseline bi-testicular volume 1.9 ± 1.0 ml, post-treatment 26 ± 15 ml (all on ultrasound) | / | In prepubertal Group A Kallmann, final sperm concentration was 37 ± 36 million/ml; in CHH absent puberty, final sperm concentration was 37 ± 48 million/ml | In prepubertal Group A Kallmann: baseline inhibin B 20 ± 8 pg/ml, post-treatment max inhibin B 112 ± 66 pg/ml; in CHH absent puberty baseline inhibin B 31 ± 19 pg/ml, post-treatment max inhibin B 198 ± 145 pg/ml |
| Shah et al. 2021 ^42^ | Congenital hypogonadotropic hypogonadism | 35 (9 severe and untreated) | Mean 23.4 ± 6.7 years (severe + untreated subgroup) | 20/35 had severe phenotype and all had TV <3ml. Baseline TV for whole cohort was 3.6 ± 2.7 ml. | 17 out of 35 patients had not received prior testosterone replacement therapy | Group 1: No prior testosterone: All the patients were concurrently initiated on hMG and hCG, hMG 75-150 U 2-3/week and initial hCG 500 U twice weekly with dose titration. | Baseline TV in those with severe phenotype and no prior testosterone therapy was 1.78 ± 0.51 ml, post-treatment 7.5 ± 3.2 ml | / | Spermatogenesis was achieved in 8 out of 9 patients (89%) with severe phenotype and no prior testosterone therapy. Peak sperm concentration 7 (4-10) million/ml. | In severe phenotype and no prior testosterone therapy: baseline serum testosterone 0.29 (0.18-0.35) ng/ml, post-treatment highest trough serum total testosterone level 5.21 (4.7-10.5) ng/ml |

| **Supplementary Table 7 – Studies using statistical analyses comparing treatment modalities across key pubertal outcomes** | | | | | | | | | | |
| --- | --- | --- | --- | --- | --- | --- | --- | --- | --- | --- |
| **Study and treatment details** | | | | **Results of statistical analyses comparing treatment arms** | | | | | | |
| **Citation** | **Population** | **Treatment** | **Age at starting treatment** | **Testicular volume** | **Penile length** | **Inhibin B** | **AMH** | **Testosterone** | **Sperm count** | **Spermatogenesis** |
| **Non-randomised studies of interventions** | | | | | | | | | | |
| **Aydogdu et al. 2013^2^** | Never-treated patients with idiopathic hypogonadotropic hypogonadism | Three treatment groups - one of HCG 5000 IU twice weekly, one of IM testosterone (Sustanon Ⓡ 250mg) once every 3 weeks, one of daily testosterone gel (Testogel Ⓡ 250 mg/5g) | Mean 21 +/- 1.4 years | Mean TV went from 1.7 +/- 1.5 ml to 2.2 +/- 1.6 ml in hCG group.  p=0.025 (change greater in hCG group vs testosterone gel and IM)  p=0.013 (change greater in hCG group vs testosterone IM)  p=0.028 (change greater in hCG group vs testosterone gel) | / | / | / | Mean testosterone went from 36.4 +/- 51.1 ng/dl to 52.7 +/- 45.4 ng/dl in hCG group. p=0.0001 (change greater in testosterone gel group vs testosterone IM and hCG)  p=0.0001 (change greater in testosterone IM vs hCG group)  p=0.0001 (change greater in testosterone gel vs hCG group) | / | / |
| **Buchter et al. 1998^55^** | Patients with hypothalamic disorders (idiopathic hypogonadotropic hypogonadism, Kallmann syndrome) and hypopituitarism | 1a) Pulsatile GnRH 5-20ug every 120 minutes subcutaneously (n=6) 1b) IM/SC hCG 1000-2500 IU twice a week and 75-150 IU hMG three times a week in 20 courses (n=18) 2) IM/SC hCG 1000-2500 IU twice a week and 75-150 IU hMG three times a week in 30 cycles (n=21) | Mean age of the patients of group Ia (IHH+ Kal, GnRH) was 30.1 +/- 5.0 years (19 -35 years), in Group Ib (IHH+Kal, hCG+hMG) mean age was 29.1 +/-7.2 years (21-43 years), while in group II (hypopituitarism, hCG/hMG) patients had a mean age of 34.1 +/- 6.1 years (23-48 years) | / | / | / | / | / | / | / |
| **Celik et al. 2021^57^** | Hypogonadotropic hypogonadism | hCG 1000-2000 IU, 2-3/ week (n=17) IM Testosterone 250mg every 21 days with dose titration (n=19) | 28.5 +/- 8.5 years | Significantly greater change in mean testicular volume both left and right, p<0.001 for hCG compared to testosterone | / | / | / | No significant difference in total testosterone between hCG vs testosterone, p=0.264 | / | / |
| **Chen et al. 2019^7^** | Isolated hypogonadotropic hypogonadism | Group 1 (n=54) intramuscular injection of 2000 IU hCG injected twice per week together with oral T undecanoate soft capsules, 40 mg twice daily. In the first 6 months, hCG doses were adjusted according to patients' serum T levels. The maximum dose of hCG used in our study was 8000 IU twice per week.  Group 2: (n=53) intramuscular injection of 2000 IU hCG injected twice per week. | hCG/TU group : 21.8 +/- 3.7 years    hCG group: 22.7 +/- 4.1 years | No significant difference in testicular volume between hCG and hCG/TU, p =0.830 | / | / | / | No significant difference in testosterone between hCG and hCG/TU, p =0.411 | No significant difference in sperm count between hCG and hCG/TU, p =0.917 | / |
| **Gong et al. 2015^16^** | Peri-pubertal boys with hypogonadotropic hypogonadism | 1) 1000 IU hCG IM twice per week for 3 months, then once every other day for 3 months, then increased to 2000 IU twice per week / 1500 IU every other day for 3 months, then 2000 IU every other day for three months (n=22) 2) 10 ug of GnRH SC injection via pump every 90 minutes (n=12) | Range 10-16 | Significantly greater TV in GnRH group vs hCG group, p<0.05 | No significant difference in penile length between GnRH vs hCG | / | / | No significant difference in testosterone between GnRH vs hCG | / | / |
| **Kliesch et al. 1994^69^** | Hypogonadotropic hypogonadal men (hypothalamic or pituitary) | 1) hCG 1000-2500 IE twice a week and 75-150 IE hMG three times a week IM or SC (IHH or Kallmann)  2) Pulsatile GnRH 5-20 ug every 120 minutes (IHH or Kallmann)  3) hCG 1000-2500 IM twice a week and 75-150 IE hMG three times a week IM or SC (pituitary-deficient) | Mean ages of the men were 31 (8;21-43) years (hCG/hMG with IHH or Kallmann), 31 (7;19-39) years (GnRH) and 34 (4;26-41) years (hCG/hMG with pituitary deficiency) | No significant difference in testicular volume between hCG/hMG and GnRH when comparing IHH/Kallmann groups, but hypopituitarism group treated with hCG/HMG had significantly greater (p<0.05) increase in testicular volume than IHH/Kallmann group treated with GnRH. | / | / | / | No significant difference in testosterone values between groups | Patients with hypopituitarism and treated with hCG + hMG had greater concentrations than IHH/Kallmann patients treated with hCG + hMG or GnRH | / |
| **Liu et al. 2021^26^** | Congenital hypogonadotropic hypogonadism | hCG/HMG n=20 group: 1000-2000 IU hCG IM injection for 3 months once every other day or twice per week, titrated to testosterone. Subsequently hMG containing 75 U of FSH and 75 U of LH was added once daily, titrated to testosterone. Duration was 6 to 21 months.  GnRH group n=21: 8 to 10 ug of GnRH (200 ug/ml) were injected SC every 90 min via an injection pump. Duration was 6-26 months. | Between 12 and 18 years | No significant difference in TV between hCG/hMG and GnRH, mean 8.8 vs 8.6ml, p=0.898 | No significant difference in penile length between hCG/hMG and GnRH, mean 8.1 vs 6.7 cm, p=0.211 | No significant difference in inhibin b between hCG/hMG and GnRH, p=0.530 | No significant difference in AMH between hCG/hMG and GnRH, p=0.836 | p<0.001 - the hCG/hMG group had significantly greater testosterone at 12 months than the GnRH group, mean 584 ng/dL vs 180 ng/dL | / | / |
| **Mao et al. 2017^27^** | Congenital hypogonadotropic hypogonadism | Group 1: 2000 IU hCG 2 times/week for 3 months then continued treatment combined with hMG 75-150 U 2 times/week for 12 months (n = 182)  Group 2: 10 ug per 90 min SC GnRH, dose adjusted to target LH and FSH between 5-10 IU/l (n = 20) | 21.5 +/- 3 (hCG/HMG group), 27.1 +/- 7 (GnRH group) | No significant difference in TV between hCG/hMG and GnRH, mean 9.0 and 10.8ml, p=0.105 | / | / | / | Significantly greater testosterone in hCG/hMG group than GnRH group, 16.2nmol/l vs 8.3nmol/l, p= 0.001 | No significant difference in sperm count between hCG/hMG and GnRH, p=0.064 | No significant difference in spermatogenesis between hCG/hMG and GnRH, 76.9% vs 70.0%, p=0.580 |
| **Ortac et al. 2020^33^** | Congenital hypogonadotropic hypogonadism | IM hCG 1500 IU twice per week for 6 months. If the patient remained azoospermic at the end of 6 months, FSH 75-150 IU twice per week (rFSH or hMG), was added. | Average 27.9 (15-51) years | / | / | / | / | No significant difference in testosterone between hMG and hCG/FSH group at time of sperm detection, post treatment, p=0.177 | / | No significant difference in spermatogenesis between hMG and hCG/FSH group, post treatment, both 85.7%, p=1.00 |
| **Sato et al. 2015** | Hypogonadotropic hypogonadism | hCG monotherapy and hCG-rFSH combination therapy; hCG dose ranging from 500 U twice a week to 4000 U three times a week; then rFSH dose ranging from 75 U twice a week/150 U once a week to 225 U three times a week; others had testosterone monotherapy. n=17 received hCG-rFSH and underwent semen testing. | Mean age 15.7 years | / | / | / | / | / | No significance difference in sperm count between those having TRT or hCG as pretreatment before hCG + FSH, p=0.07 |  |
| **Schopohl 1993^40^** | Male patients with idiopathic hypothalamic hypogonadism or Kallmann syndrome | 3x2500 IU hCG per week IM with dose titrations. After 2-3 months, 2x 150 IU of hMG were added, with dose titration. (n=18)  GnRH with pump started at 4ug/bolus up to 16ug/bolus if no pituitary response seen (n=18) | 23.6 ± 7.3 years (gonadotropin group) and 21.1 ± 3.0 years (GnRH group) | Testicular increase was significantly higher in GnRH then hCG/hMG group (mean increase 8.1 +/- 2.0ml vs 4.8 +/- 1.8 ml) p<0.001 | / | / | / | Testosterone was significantly higher with hCG/hMG than during GnRH (22.5 ± 8.1 versus 16.8 ± 5.5 nmol/l) p<0.03 | / | Greater proportion of GnRH group achieved spermatogenesis, at earlier time; 12 ± 1.6 months for GnRH, 20 ± 2.3 months for hCG/hMG (p < 0.02) |
| **Shirashi et al. 2021^94^** | Hypogonadotropic hypogonadism | Continue testosterone replacement IM 250mg testosterone enanthate every 3 weeks (all patients) then to continue this treatment (n=10) or hCG 3000 IU twice a week titrated to testosterone/side effects (n=16) | hCG mean 34.2 ± 5.2 years, TRT mean 34.7 ± 4.1 years | Significantly greater testicular volume in hCG group compared to TRT group (17.7 ± 1.9 vs 9.4 ± 2.3) p<0.0001 | / | / | / | / | Significantly greater sperm concentration in hCG group than TRT group p<0.01 | Significantly greater spermatogenesis in hCG group than TRT group (81% vs 30%) <0.01 |
| **Sinisi et al. 2010^96^** | Hypogonadotropic hypogonadal men with failed induction of spermatogenesis on hCG alone | All patients received 2000 IU hCG twice a week for several months before introducing rFSH, with arms: 1) rFSH 75 IU twice a week (n=10) 2) rFSH 75 IU three times a week (n=10) 3) hp-FSH 75 IU twice a week (n=7) 4) hp-FSH 75 IU three times a week (n=7) | Age range between 22.7 +/- 1.5 to 24.9 +/- 1.7 years across four groups | No significant difference between groups (hCG and 4 FSH regimens) for final testicular volumes. | / | / | / | / | No significant difference in sperm count between rFSH and hpFSH. | / |
| **Trabado et al. 2011^44^** | Men with congenital hypogonadotropic hypogonadism, treated or untreated | Combination therapy of hCG (1500 IU IM twice/three times weekly) and recombinant/extractive human FSH (150 IU SC twice/three times weekly) n=88  Testosterone enanthate (n=101) 250mg IM every 3 weeks | Mean 28.8+/- 10.1 (CHH) | / | / | / | / | Bioavailable and free testosterone significantly greater in patients with CHH receiving testosterone vs combined gonadotropin therapy (p<0.05) but total testosterone – no difference | / | / |
| **Yang et al. 2012^101^** | Hypogonadotropic hypogonadism | Group 1: IM hCG 2,000 IU, every 3 days, n=84  Group 2: Combination of hCG/hMG (2,000 IU/ 75 IU, every 3 days) n=74  Group 3: n=84, Oral testosterone undecanoate, 120-160 mg per day for 2-3 weeks and then dosage adjusted to 40-120 mg per day for maintenance | Mean: 23.1 +/- 6.2 years age range: 15-52 years | Increase in testicular size in the hMG/hCG group was significantly more than in hCG (p=0.012) | / | / | / | / | The difference in sperm count was not statistically different between hCG and hCG+hMG p=0.641 | / |
| **Zacharin et al. 2012^103^** | Males with Hypogonadotropic hypogonadism | Group 1 (n=9): hCG alone, initially 500 IU twice-weekly with increases to 1000 IU at 6 months and as puberty progressed, to 1500 IU twice-weekly  Group 2 (n=10): 500-1500 IU hCG twice-weekly, with addition of 150-300 IU recombinant FSH three-times-weekly | Median - 18.9 years (14.5-31.0) | No significant difference in testicular volume between hCG and hCG+FSH at 9 months, p = 0.11 | / | No significant difference in inhibin B between hCG and hCG+FSH, p = 0.33 | / | No significant difference in testosterone between hCG and hCG+FSH, p = 0.10 | / | There was a significantly greater rate of spermatogenesis at 9 months in FSH+hCG group than hCG alone group, p = 0.003 |
| **Zhang et al. 2019^50^** | Azoospermic men with congenital hypogonadotropic hypogonadism | Group 1: Pulsatile gonadorelin Pump (PCP) n=10. Gonadorelin (GnRH analogue) 10 ug every 90 min SC via infusion pumps with dose titration  Group 2 hCG/hMG combined n=18. hCG IM 3-6 months 2000 IU three times per week until the serum testosterone level was >3 ng/ml or testicular volume = 3 ml. Then hMG (75 IU three times per week) was added for 3 months, followed by hCG alone again for another 3 months and then HCG +HMG for an additional 3 months cyclically. | hCG/hMG group: 24 ± 5 (16-34) years  Pulsatile GnRH group: 27 ± 3 (23-32) years | No significant difference in testicular volume between GnRH and hCG/hMG after treatment (p-value not stated) | No significant difference in penile length between GnRH and hCG/hMG after treatment (p-value not stated) | / | / | No significant difference in testosterone between GnRH and hCG/hMG after treatment (p-value not stated) | / | GnRH significantly more likely to achieve spermatogenesis then hCG/hMG, p=0.038, in Cox proportional hazards regression analysis, but no significant difference in χ2 analysis and no-inferior analysis |
| **Zhao et al. 2013^51^** | Idiopathic hypogonadotropic hypogonadism | hCG or hCG + hMG were combined in gonadotropin group (n=37) hCG + Testosterone or hCG + hMG + testosterone (n=25) | Population age 27.3 ± 3.4 years | No significant difference in testicular volume between gonadotropin + androgens and gonadotropin groups, p=0.419 | Gonadotropin + androgen group had significantly greater penis length than gonadotropin group (p=0.002) | / | / | Gonadotropin + androgens group had significantly greater proportion achieving normalisation of testosterone (p=0.004) | / | / |
| **Randomised controlled trials** | | | | | | | | | | |
| **Bouloux et al. 2003^53^** | Hypogonadotropic hypogonadism | All patients 1400 IU hCG twice a week with titration to testosterone levels for 16 weeks. Subsequently, Group A: 225 IU twice a week FSH SC for 48 weeks (n=15). Group B: 150 IU thrice a week FSH SC for 48 weeks (n=15. | Group A (225 IU twice a week FSH) - mean 30.1 years (5.6); or Group B (150 IU FSH thrice a week) - mean 30.7 years (8.0) | / | / | / | / | / | / | Onset of spermatogenesis had no significant difference between FSH groups (P>0.05) |
| **Dwyer et al. 2013^11^** | Congenital hypogonadotropic hypogonadism (GnRH-deficient men with prepubertal testes (4 mL), no cryptorchidism, and no prior gonadotropin therapy) | Either 24 months of pulsatile GnRH (n=6) or 4 months pretreatment with rFSH (75-150 IU SC OD) followed by 24 months of pulsatile GnRH (n=7) | Mean 28 +/- 2 in rFSH pretreatment group, mean 24 +/- 2 in GnRH only group | No significant difference in TV between groups (GnRH vs rFSH + GnRH) - p=0.06 for difference | / | / | / | / | No significant difference in sperm count between groups (GnRH vs rFSH + GnRH). 2/6 in GnRH group were azoospermic compared to none of the pre-treated group. Authors noted study underpowered. | / |
| **Liu et al. 2017^25^** | Isolated hypogonadotropic hypogonadism | Group A: 2000 U hCG twice a week for 18 months and 75 U uFSH three times a week every other 3 months for 6-18 months Group B: 2000 U hCG twice a week for 18 months and 75 U uFSH three times a week every other 3 months for 6-18 months PLUS oral 40mg/day zinc for 18 months | The mean age of the IHH patients was 24.1 ± 4.9 years in Group A (hCG/FSH) and 22.3 ± 4.0 years in Group B (FSH/zinc). | No significant difference in TV between groups, p=0.54 (hCG+FSH median 5.3 ml vs hCG+FSH+zinc median 4.2 ml) | No significant difference in penile length between groups, p=0.88 (hCG+FSH median 6.9 cm vs hCG+FSH+zinc median 6.9 cm) | / | / | No significant difference in testosterone between groups, p=0.28 (hCG+FSH vs hCG+FSH+zinc) | No significant difference in sperm concentration between groups, p=0.91 (hCG+FSH vs hCG+FSH+zinc) | No significant difference in spermatogenesis between groups, p=0.66 (hCG+FSH 64.7% vs hCG+FSH+zinc 69.7%) |
| **Schaison et al. 1993^39^** | Hypogonadotropic hypogonadism without cryptorchidism | Crossover study: 150 U of hMG was administered three times weekly along with 1500 IU hCG for 24 months, followed by urinary FSH 150 IU three times a week plus testosterone enanthate 250mg IM once weekly, for 24 months (n=5) or the other way round (n=5) | Age 23-35 years | / | / | / | / | Testosterone levels were significantly higher during FSH plus testosterone treatment than hMG-hCG, p<0.05 | No significant difference in sperm count between T+FSH vs HCG+hMG, p=not significant | / |
| **Zhang et al. 2015^49^** | Idiopathic hypogonadotropic hypogonadism | All patients received 2000 U hCG twice a week from start to 18th month, IM. Group A: 75 U uFSH three times a week from 6-18th month, IM (n=33) and Group B: 75 U uFSH three times a week from 6-18th month, every other 3 months, IM (n=34). | Mean 23.1 +/- 5.1 years in continual FSH group, and 24.1+/- 4.9 years in sequential FSH group | No significant difference in final testicular volume between continual vs sequential uFSH + hCG; median 4.8ml vs 5.3 ml, p=0.662 | No significant difference in final penile length between continual vs sequential uFSH + hCG, mean 6.7 vs 6.9 cm, p=0.912 | / | / | No significant difference in final testosterone between continual vs sequential uFSH + hCG, median 400 ng/dl vs 420 ng/dl, p=0.288 | No significant difference in final sperm concentration between continual vs sequential uFSH + hCG, median 1.1 (×10^6/ml) vs 1.5, p=0.317 | / |

| **Supplementary Table 8: Comparison of gonadotropin therapies across key outcomes** | |
| --- | --- |
| **Outcome** | **Treatment regimens** |
| **Testicular volume** | **hCG vs Testosterone**  hCG superior – 3 studies^2, 57, 94^  **hCG vs hCG + testosterone**  No difference – 1 study^7^  **GnRH vs hCG**  GnRH superior – 1 study^16^  **hCG + hMG vs GnRH**  No difference – 3 studies^26, 27, 50^  Inconsistent effect - 1 study^69^  GnRH superior – 1 study^40^  **hCG + four variable FSH regimens**  No difference – 1 study^96^  **hCG + hMG vs hCG**  hMG/HCG superior – 1 study^101^  **hCG vs hCG + FSH**  No difference – 1 study^103^  **hCG or hCG + hMG vs gonadotropins + testosterone**  No difference – 1 study^51^  **GnRH vs GnRH + FSH**  No difference – 1 study^11^  **hCG + FSH vs hCG + FSH + zinc**  No difference – 1 study^25^  **Continual vs sequential FSH + hCG**  No difference – 1 study^49^ |
| **Penile length** | **GnRH vs hCG**  No difference – 1 study^16^  **hCG + hMG vs GnRH**  No difference – 2 studies^26, 50^  **hCG or hCG + hMG vs gonadotropins + testosterone**  Gonadotropins + testosterone superior – 1 study^51^  **hCG + FSH vs hCG + FSH + zinc**  No difference – 1 study^25^  **Continual vs sequential FSH + hCG**  No difference – 1 study^49^ |
| **Inhibin B** | **hCG + hMG vs GnRH**  No difference – 1 study^26^  **hCG vs hCG + FSH**  No difference – 1 study^103^ |
| **AMH** | **hCG + hMG vs GnRH**  No difference – 1 study^26^ |
| **Testosterone** | **hCG vs testosterone**  Testosterone superior – 1 study^2^  No difference – 1 study^57^  **hCG + FSH vs Testosterone**  Total testosterone no difference, bioavailable and free testosterone higher – 1 study^44^  **hCG vs hCG + testosterone**  No difference – 1 study^7^  **GnRH vs hCG**  No difference – 1 study^16^  **hCG + hMG vs GnRH**  hCG/hMG superior – 3 studies^26, 27, 40^  No difference – 2 studies^50, 69^  **hMG vs hCG + FSH**  No difference – 1 study^33^  **hCG vs hCG + FSH**  No difference – 1 study^103^  **hCG or hCG + hMG (gonadotropins) vs gonadotropins + testosterone**  Gonadotropins + testosterone superior – 1 study^51^  **hCG+ FSH vs hCG + FSH + zinc**  No difference – 1 study^25^  **FSH + testosterone vs hMG + hCG**  FSH+testosterone superior – 1 study^39^  **Continual vs sequential FSH + hCG**  No difference – 1 study^49^ |
| **Sperm count or concentration** | **hCG vs hCG + testosterone**  No difference – 1 study^7^  **hCG+ hMG vs GnRH**  No difference – 1 study^27^  Inconsistent effect – 1 study^69^  **hCG vs testosterone**  hCG superior – 1 study^94^  **hCG > hCG + FSH vs testosterone > hCG + FSH**  No difference – 1 study^91^  **CG + four variable FSH regimens**  No difference – 1 study^96^  **hMG + HCG vs hCG**  No difference – 1 study^101^  **GnRH vs GnRH + FSH**  No difference – 1 study^11^  **hCG + FSH vs hCG + FSH + zinc**  No difference – 1 study^25^  **FSH + testosterone first (in crossover study) vs hMG + hCG first**  No difference – 1 study^39^  **Continual vs sequential FSH + hCG**  No difference – 1 study^49^ |
| **Spermatogenesis rate** | **hCG + hMG vs GnRH**  No difference – 1 study^27^  Inconsistent effect - 1 study^50^  **hCG vs hCG + testosterone**  No difference – 1 study^7^  **hMG vs hCG + FSH**  No difference – 1 study^33^  **hCG vs testosterone**  hCG superior – 1 study^94^  **hCG vs hCG + FSH**  hCG+FSH superior – 1 study^103^  **hCG + comparison of two FSH regimens**  No difference – 1 study^53^  **hCG + FSH vs hCG + FSH + zinc**  No difference – 1 study^25^ |

**References**

1. Abbasi H, Dadkhah A, Moshtaghi D & Hamiditabar MA. Gonadotropins in infertile men with idiopathic hypogonadotropic hypogonadism. *International Journal of Fertility and Sterility* 2008 **2** 113-114.

2. Aydogdu A, Bolu E, Sonmez A, Tasci I, Haymana C, Acar R, Meric C, Taslipinar A, Ozgurtas T & Azal O. Effects of three different medications on metabolic parameters and testicular volume in patients with hypogonadotropic hypogonadism: 3-year experience. *Clinical Endocrinology* 2013 **79** 243-251.

3. Bayram F, Elbuken G, Korkmaz C, Aydogdu A, Karaca Z & Caklr I. The Effects of Gonadotropin Replacement Therapy on Metabolic Parameters and Body Composition in Men with Idiopathic Hypogonadotropic Hypogonadism. *Hormone and Metabolic Research* 2015 **48** 112-117.

4. Boepple PA, Hayes FJ, Dwyer AA, Raivio T, Lee H, Crowley Jr WF & Pitteloud N. Relative roles of inhibin B and sex steroids in the negative feedback regulation of follicle-stimulating hormone in men across the full spectrum of seminiferous epithelium function. *Journal of Clinical Endocrinology and Metabolism* 2008 **93** 1809-1814.

5. Bouloux P, Warne DW & Loumaye E. Efficacy and safety of recombinant human follicle-stimulating hormone in men with isolated hypogonadotropic hypogonadism. *Fertility and Sterility* 2002 **77** 270-273.

6. Cangiano B, Goggi G, Federici S, Bresesti C, Cotellessa L, Guizzardi F, Vezzoli V, Duminuco P, Persani L & Bonomi M. Predictors of reproductive and non-reproductive outcomes of gonadotropin mediated pubertal induction in male patients with congenital hypogonadotropic hypogonadism (CHH). *Journal of Endocrinological Investigation* 2021 **44** 2445-2454.

7. Chen YW, Niu YH, Xu H, Wang DQ, Jiang HY, Pokhrel G, Wang T, Wang SG & Liu JH. Testosterone undecanoate supplementation together with human chorionic gonadotropin does not impair spermatogenesis in males with isolated hypogonadotropic hypogonadism: A retrospective study. *Asian Journal of Andrology* 2019 **21** 413-418.

8. Chen Y, Sun T, Niu Y, Wang D, Xiong Z, Li C, Liu K, Qiu Y, Sun Y, Gong J, Wang T, Wang S, Xu H & Liu J. Correlations Among Genotype and Outcome in Chinese Male Patients With Congenital Hypogonadotropic Hypogonadism Under HCG Treatment. *Journal of Sexual Medicine* 2020 **17** 645-657.

9. Christiansen P & Skakkebaek NE. Pulsatile gonadotropin-releasing hormone treatment of men with idiopathic hypogonadotropic hypogonadism. *Hormone Research* 2002 **57** 32-36.

10. Delemarre-Van de Waal HA & Odink RJ. Pulsatile GnRH treatment in boys and girls with idiopathic hypogonadotrophic hypogonadism. *Human reproduction (Oxford, England)* 1993 **8 Suppl 2** 180-183.

11. Dwyer AA, Sykiotis GP, Hayes FJ, Boepple PA, Lee H, Loughlin KR, Dym M, Sluss PM, Crowley Jr WF & Pitteloud N. Trial of recombinant follicle-stimulating hormone pretreatment for GnRH-induced fertility in patients with congenital hypogonadotropic hypogonadism. *Journal of Clinical Endocrinology and Metabolism* 2013 **98** E1790-E1795.

12. Anonymous. Efficacy and safety of highly purified urinary follicle-stimulating hormone with human chorionic gonadotropin for treating men with isolated hypogonadotropic hypogonadism. *Fertility and Sterility* 1998 **70** 256-262.

13. Fahmy I, Kamal A, Shamloul R, Mansour R, Serour G & Aboulghar M. ICSI using testicular sperm in male hypogonadotrophic hypogonadism unresponsive to gonadotrophin therapy. *Human Reproduction* 2004 **19** 1558-1561.

14. Fan HQ, Wang YC, He W, Zhou HW & Yang T. Changes in levels of testosterone, insulin sensitivity and metabolic profiles during GnRH therapy: Reciprocity between insulin sensitivity and pituitary responsiveness to GnRH in teenage and young male patients with congenital hypogonadotropic hypogonadis. *Clinical Endocrinology* 2022 **97** 783-791.

15. Fuse H, Akashi T, Kazama T & Katayama T. Gonadotropin therapy in males with hypogonadotropic hypogonadism: Factors affecting induction of spermatogenesis after gonadotropin replacement. *International Urology and Nephrology* 1996 **28** 367-374.

16. Gong C, Liu Y, Qin M, Wu D & Wang X. Pulsatile GnRH is superior to HCG in therapeutic efficacy in adolescent boys with hypogonadotropic hypogonadodism. *Journal of Clinical Endocrinology and Metabolism* 2015 **100** 2793-2799.

17. Hao M, Nie M, Yu BQ, Gao YJ, Wang X, Ma WL, Huang QB, Zhang R, Mao JF & Wu XY. Gonadotropin treatment for male partial congenital hypogonadotropic hypogonadism in Chinese patients. *Asian Journal of Andrology* 2020 **22** 390-395.

18. Hao M, Mao JF, Guan QB, Tian L, Han H, Lei HE, Zheng DM, Tian ZH, Nie M, Wang X, Yu BQ, Gao YJ & Wu XY. Efficacy and safety of pulsatile gonadotropin-releasing hormone therapy in patients with congenital hypogonadotropic hypogonadism: a multicentre clinical study. *Annals of Translational Medicine* 2021 **9** 962.

19. Hosseinifar H, Sabbaghian M, Chehrazi M, Modarresi T, Alipour FJ & Sadighi Gilani MA. Assessment of deoxyribonucleic acid fragmentation index, testicular volume, semen parameters, and hormone profile in gonadotropin-treated men with hypogonadotropic hypogonadism. *Urology* 2013 **82** 1291-1295.

20. Kilciler G, Ozata M, Oktenli C, Yavuz Sanisoglu S, Bolu E, Bingol N, Kilciler M, Caglayan Ozdemir I & Kutlu M. Diurnal leptin secretion is intact in male hypogonadotropic hypogonadism and is not influenced by exogenous gonadotropins. *Journal of Clinical Endocrinology and Metabolism* 2002 **87** 5023-5029.

21. Kirk JMW, Savage MO, Grant DB, Bouloux PMG & Besser GM. Gonadal function and response to human chorionic and menopausal gonadotrophin therapy in male patients with idiopathic hypogonadotrophic hypogonadism. *Clinical Endocrinology* 1994 **41** 57-63.

22. Li S, Zhao Y, Nie M, Ma W, Wang X, Ji W, Yang Y, Hao M, Yu B, Gao Y, Mao J & Wu X. Clinical characteristics and spermatogenesis in patients with congenital hypogonadotropic hypogonadism caused by FGFR1 mutations. *International Journal of Endocrinology* 2020 **2020** 8873532.

23. Liu Z, Mao J, Xu H, Wang X, Huang B, Zheng J, Nie M, Zhang H & Wu X. Gonadotropin-Induced Spermatogenesis in CHH Patients with Cryptorchidism. *International Journal of Endocrinology* 2019 **2019** 6743489.

24. Liu Z, Mao J, Wu X, Xu H, Wang X, Huang B, Zheng J, Nie M & Zhang H. Efficacy and outcome predictors of gonadotropin treatment for male congenital hypogonadotropic hypogonadism: A retrospective study of 223 patients. *Medicine (United States)* 2016 **95** e2867.

25. Liu YL, Zhang MN, Tong GY, Sun SY, Zhu YH, Cao Y, Zhang J, Huang H, Niu B, Li H, Guo QH, Gao Y, Zhu DL & Li XY. The effectiveness of zinc supplementation in men with isolated hypogonadotropic hypogonadism. *Asian Journal of Andrology* 2017 **19** 280-285.

26. Liu Y, Ren XY, Peng YG, Chen SK, Cheng XR, Qin M, Wang XL, Song YN, Fan LJ & Gong CX. Efficacy and safety of human chorionic gonadotropin combined with human menopausal gonadotropin and a gonadotropin-releasing hormone pump for male adolescents with congenital hypogonadotropic hypogonadism. *Chinese medical journal* 2021 **134** 1152-1159.

27. Mao JF, Liu ZX, Nie M, Wang X, Xu HL, Huang BK, Zheng JJ, Min L, Kaiser U & Wu XY. Pulsatile gonadotropin-releasing hormone therapy is associated with earlier spermatogenesis compared to combined gonadotropin therapy in patients with congenital hypogonadotropic hypogonadism. *Asian Journal of Andrology* 2017 **19** 680-685.

28. Musabak U, Bolu E, Ozata M, Oktenli C, Sengul A, Inal A, Yesilova Z, Kilciler G, Ozdemir IC & Kocar IH. Gonadotropin treatment restores in vitro interleukin-1beta and tumour necrosis factor-alpha production by stimulated peripheral blood mononuclear cells from patients with idiopathic hypogonadotropic hypogonadism. *Clinical and Experimental Immunology* 2003 **132** 265-270.

29. Nachtigall LB, Boepple PA, Seminara SB, Khoury RH, Sluss PM, Lecain AE & Crowley WF, Jr. Inhibin B secretion in males with gonadotropin-releasing hormone (GnRH) deficiency before and during long-term GnRH replacement: relationship to spontaneous puberty, testicular volume, and prior treatment--a clinical research center study. *The Journal of Clinical Endocrinology & Metabolism* 1996 **81** 3520-3525.

30. Nachtigall LB, Boepple PA, Pralong FP & Crowley Jr WF. Adult-onset idiopathic hypogonadotropic hypogonadism - A treatable form of male infertility. *New England Journal of Medicine* 1997 **336** 410-415.

31. Nie M, Yu B, Chen R, Sun B, Mao J, Wang X, Zhang H & Wu X. Novel rare variants in FGFR1 and clinical characteristics analysis in a series of congenital hypogonadotropic hypogonadism patients. *Clinical Endocrinology* 2021 **95** 153-162.

32. Oktenli C, Yesilova Z, Ozata M, Yaman H, Tuzun A, Dundar S, Sanisoglu SY, Musabak U, Erbil MK & Dagalp K. Gonadotropin treatment increases homocysteine levels in idiopathic hypogonadotropic hypogonadism: An indirect effect mediated by changes in body composition. *Journal of Endocrinology* 2003 **179** 35-39.

33. Ortac M, Hidir M, Cilesiz NC & Kadioglu A. Efficacy of follitropin-alpha versus human menopausal gonadotropin for male patients with congenital hypogonadotropic hypogonadism. *Turkish journal of urology* 2020 **46** 13-17.

34. Ortac M, Hidir M, Salabas E, Boyuk A, Bese C, Pazir Y & Kadioglu A. Evaluation of gonadotropin-replacement therapy in male patients with hypogonadotropic hypogonadism. *Asian Journal of Andrology* 2019 **21** 623-627.

35. Pitteloud N, Hayes FJ, Dwyer A, Boepple PA, Lee H & Crowley Jr WF. Predictors of outcome of long-term GnRH therapy in men with idiopathic hypogonadotropic hypogonadism. *Journal of Clinical Endocrinology and Metabolism* 2002 **87** 4128-4136.

36. Rohayem J, Zitzmann M, Laurentino S, Kliesch S, Nieschlag E, Holterhus PM & Kulle A. The role of gonadotropins in testicular and adrenal androgen biosynthesis pathways-Insights from males with congenital hypogonadotropic hypogonadism on hCG/rFSH and on testosterone replacement. *Clinical Endocrinology* 2021 **94** 90-101.

37. Saal W, Happ J, Cordes U, Baum RP & Schmidt M. Subcutaneous gonadotropin therapy in male patients with hypogonadotropic hypogonadism. *Fertility and Sterility* 1991 **56** 319-324.

38. Sanyal D & Chatterjee S. Treatment preferences and outcome in male hypogonadotropic hypogonadism: An Indian perspective. *Andrologia* 2016 **48** 601-602.

39. Schaison G, Young J, Pholsena M, Nahoul K & Couzinet B. Failure of combined follicle-stimulating hormone-testosterone administration to initiate and/or maintain spermatogenesis in men with hypogonadotropic hypogonadism. *Journal of Clinical Endocrinology and Metabolism* 1993 **77** 1545-1549.

40. Schopohl J. Pulsatile gonadotrophin releasing hormone versus gonadotrophin treatment of hypothalamic hypogonadism in males. *Human Reproduction* 1993 **8** 175-179.

41. Schopohl J, Mehltretter G, von Zumbusch R, Eversmann T & von Werder K. Comparison of gonadotropin-releasing hormone and gonadotropin therapy in male patients with idiopathic hypothalamic hypogonadism**Presented in part at the 31th Meeting of the German Endocrine Society, Münster, Germany, March 4 to 7, 1987 and at the 70th Meeting of the American Endocrine Society, New Orleans, Louisiana, June 8 to 11, 1988. *Fertility and Sterility* 1991 **56** 1143-1150.

42. Shah R, Patil V, Sarathi V, Lila AR, Zacharin M, Krishnappa B, Sehemby M, Jaiswal SK, Jadhav PL, Ramteke-Jadhav S, Shah N & Bandgar T. Prior testosterone replacement therapy may impact spermatogenic response to combined gonadotropin therapy in severe congenital hypogonadotropic hypogonadism. *Pituitary* 2021 **24** 326-333.

43. Sykiotis GP, Hoang XH, Avbelj M, Hayes FJ, Thambundit A, Dwyer A, Au M, Plummer L, Crowley Jr WF & Pitteloud N. Congenital idiopathic hypogonadotropic hypogonadism: Evidence of defects in the hypothalamus, pituitary, and testes. *Journal of Clinical Endocrinology and Metabolism* 2010 **95** 3019-3027.

44. Trabado S, Maione L, Salenave S, Baron S, Galland F, Bry-Gauillard H, Guiochon-Mantel A, Chanson P, Pitteloud N, Sinisi AA, Brailly-Tabard S & Young J. Estradiol levels in men with congenital hypogonadotropic hypogonadism and the effects of different modalities of hormonal treatment. *Fertility and Sterility* 2011 **95** 2324-2329.e2323.

45. Vicari E, Mongioi A, Calogero AE, Moncada ML, Sidoti G, Polosa P & D'Agata R. Therapy with human chorionic gonadotrophin alone induces spermatogenesis in men with isolated hypogonadotrophic hypogonadism - long-term follow-up. *International Journal of Andrology* 1992 **15** 320-329.

46. Yesilova Z, Ozata M, Kocar IH, Turan M, Pekel A, Sengul A & Ozdemir IC. The effects of gonadotropin treatment on the immunological features of male patients with idiopathic hypogonadotropic hypogonadism. *Journal of Clinical Endocrinology and Metabolism* 2000 **85** 66-70.

47. Yilmazel FK, Karabulut I, Yilmaz AH, Keskin E, Bedir F & Ozbey I. A review of hypogonadotropic hypogonadism cases followed up in our clinic in the last decade. *Urologia Journal* 2021 **88** 50-55.

48. Zhang L, Gao Y, Du Q, Liu L, Li Y, Dey SK, Banerjee S & Liao Z. Genetic Profiles and Three-year Follow-up Study of Chinese Males With Congenital Hypogonadotropic Hypogonadism. *Journal of Sexual Medicine* 2021 **18** 1500-1510.

49. Zhang M, Tong G, Liu Y, Mu Y, Weng J, Luo Z, Xue Y, Shi L, Wu X, Sun S, Zhu Y, Cao Y, Zhang J, Huang H, Niu B, Li H, Guo Q, Gao Y, Li Z, Ning G, Zhu D & Li X. Sequential versus continual purified urinary FSH/hCG in men with idiopathic hypogonadotropic hypogonadism. *Journal of Clinical Endocrinology and Metabolism* 2015 **100** 2449-2455.

50. Zhang L, Cai K, Wang Y, Ji W, Cheng Z, Chen G & Liao Z. The Pulsatile Gonadorelin Pump Induces Earlier Spermatogenesis Than Cyclical Gonadotropin Therapy in Congenital Hypogonadotropic Hypogonadism Men. *American journal of men's health* 2019 **13** 1557988318818280.

51. Zhao W, Ye H, Zhao X, Zhang Z, Sun S, Jiang Y, He M, Xu C, Hu R & Li Y. A network investigation on idiopathic hypogonadotropic hypogonadism in China. *International Journal of Endocrinology* 2013 **2013** 591012.

52. Barrio R, De Luis D, Alonso M, Lamas A & Moreno JC. Induction of puberty with human chorionic gonadotropin and follicle- stimulating hormone in adolescent males with hypogonadotropic hypogonadism. *Fertility and Sterility* 1999 **71** 244-248.

53. Bouloux PMG, Nieschlag E, Burger HG, Skakkebaek NE, Wu FCW, Handelsman DJ, Baker GHW, Ochsenkuehn R, Syska A, McLachlan RI, Giwercman A, Conway AJ, Turner L, Van Kuijk JHM & Voortman G. Induction of Spermatogenesis by Recombinant Follicle-Stimulating Hormone (Puregon) in Hypogonadotropic Azoospermic Men Who Failed to Respond to Human Chorionic Gonadotropin Alone. *Journal of Andrology* 2003 **24** 604-611.

54. Bouvattier C, Tauber M, Jouret B, Chaussain JL & Rochiccioli P. Gonadotropin treatment of hypogonadotropic hypogonadal adolescents. *Journal of Pediatric Endocrinology and Metabolism* 1999 **12** 339-344.

55. Buchter D, Behre HM, Kliesch S & Nieschlag E. Pulsatile GnRH or human chorionic gonadotropin/human menopausal gonadotropin as effective treatment for men with hypogonadotropic hypogonadism: A review of 42 cases. *European Journal of Endocrinology* 1998 **139** 298-303.

56. Burgues S & Calderon MD. Subcutaneous self-administration of highly purified follicle stimulating hormone and human chorionic gonadotrophin for the treatment of male hypogonadotrophic hypogonadism. *Human Reproduction* 1997 **12** 980-986.

57. Celik M, Ozcelik S, Bas S, Sariaydin M, Ozcelik M & Gozu H. Role of testosterone to estradiol ratio in predicting the efficacy of recombinant human chorionic gonadotropin and testosterone treatment in male hypogonadism. *Archives of endocrinology and metabolism* 2021 **65** 617-624.

58. Chen YK, Huang IS, Chen WJ, Huang CY, Ho CH, Huang EYH & Huang WJ. Reproductive outcomes of microdissection testicular sperm extraction in hypogonadotropic hypogonadal azoospermic men after gonadotropin therapy. *Journal of Assisted Reproduction and Genetics* 2021 **38** 2601-2608.

59. Delemarre-Van De Waal HA. Induction of testicular growth and spermatogenesis by pulsatile, intravenous administration of gonadotrophin-releasing hormone in patients with hypogonadotrophic hypogonadism. *Clinical Endocrinology* 1993 **38** 473-480.

60. Depenbusch M, von Eckardstein S, Simoni M & Nieschlag E. Maintenance of spermatogenesis in hypogonadotropic hypogonadal men with human chorionic gonadotropin alone. *European Journal of Endocrinology* 2002 **147** 617-624.

61. Efesoy O, Cayan S & Akbay E. The efficacy of recombinant human follicle-stimulating hormone in the treatment of various types of male-factor infertility at a single university hospital. *Journal of Andrology* 2009 **30** 679-684.

62. Farhat R, Al-zidjali F & Alzahrani AS. Outcome of gonadotropin therapy for male infertility due to hypogonadotrophic hypogonadism. *Pituitary* 2010 **13** 105-110.

63. Farshchi H, Shahnazi A & Azizi F. Effects of testosterone and gonadotropin therapy in men with hypogonadotropic hypogonadism. *International Journal of Endocrinology and Metabolism* 2009 **7** 242-247.

64. Giagulli VA, Triggiani V, Corona G, Carbone MD, Tafaro E, Licchelli B, Resta F, Sabba C, Maggi M & Guastamacchia E. Effectiveness of gonadotropin administration for spermatogenesis induction in Hypogonadotropic hypogonadism: A possible role of androgen receptor CAG repeat polymorphism and therapeutic measures. *Endocrine, Metabolic and Immune Disorders - Drug Targets* 2012 **12** 236-242.

65. Gu Y, Zhang S, Li X, Dou J, Lyu Z, Ba J, Guo Q, Zang L, Chen K, Du J, Pei Y, Mu Y-M & Gu W. The efficacy of pulsatile gonadotropin-releasing hormone therapy in male patients with hypogonadism caused by hypopituitarism. *Annals of Palliative Medicine* 2021 **10** 4642-4651.

66. Guo CY, Jones TH & Eastell R. Treatment of isolated hypogonadotropic hypogonadism effect on bone mineral density and bone turnover. *Journal of Clinical Endocrinology and Metabolism* 1997 **82** 658-665.

67. Huang Q, Mao J, Wang X, Yu B, Ma W, Ji W, Zhu Y, Zhang R, Sun B, Zhang J, Nie M & Wu X. Efficacy of Pulsatile Gonadotropin-Releasing Hormone Therapy in Male Patients: Comparison between Pituitary Stalk Interruption Syndrome and Congenital Hypogonadotropic Hypogonadism. *Endocrine Practice* 2022 **28** 521-527.

68. Ishikawa T, Ooba T, Kondo Y, Yamaguchi K & Fujisawa M. Assessment of gonadotropin therapy in male hypogonadotropic hypogonadism. *Fertility and Sterility* 2007 **88** 1697-1699.

69. Kliesch S, Behre HM & Nieschlag E. High efficacy of gonadotropin or pulsatile gonadotropin-releasing hormone treatment in hypogonadotropic hypogonadal men. *European Journal of Endocrinology* 1994 **131** 347-354.

70. Kung AWC, Zhong YY, Lam KSL & Wang C. Induction of spermatogenesis with gonadotrophins in Chinese men with hypogonadotrophic hypogonadism. *International Journal of Andrology* 1994 **17** 241-247.

71. Lin J, Mao J, Wang X, Ma W, Hao M & Wu X. Optimal treatment for spermatogenesis in male patients with hypogonadotropic hypogonadism. *Medicine* 2019 **98** e16616.

72. Liu PY, Baker HWG, Jayadev V, Zacharin M, Conway AJ & Handelsman DJ. Induction of spermatogenesis and fertility during gonadotropin treatment of Gonadotropin-Deficient infertile men: Predictors of fertility outcome. *Journal of Clinical Endocrinology and Metabolism* 2009 **94** 801-808.

73. Liu PY, Xirner LT, Rushford D, McDonald J, Baker HW, Conway AJ & Handelsman DJ. Efficacy and safety of recombinant human follicle stimulating hormone (Gonal-F) with urinary human chorionic gonadotrophin for induction of spermatogenesis and fertility in gonadotrophin-deficient men. *Human Reproduction* 1999 **14** 1540-1545.

74. Liu PY, Gebski VJ, Turner L, Conway AJ, Wishart SM & Handelsman DJ. Predicting pregnancy and spermatogenesis by survival analysis during gonadotrophin treatment of gonadotrophin-deficient infertile men. *Human Reproduction* 2002 **17** 625-633.

75. Ma W, Mao J, Nie M, Wang X, Zheng J, Liu Z, Yu B, Xiong S, Hao M, Gao Y, Ji W, Huang Q, Zhang R, Li S, Zhao Y, Sun B & Wu X. Gonadotropin Therapy Once a Week for Spermatogenesis in Hypogonadotropic Hypogonadism. *Endocrine Practice* 2021 **27** 1119-1127.

76. Mao J, Xu H, Wang X, Huang B, Liu Z, Zhen J, Nie M, Min L & Wu X. Congenital combined pituitary hormone deficiency patients have better responses to gonadotrophin-induced spermatogenesis than idiopathic hypogonadotropic hypogonadism patients. *Human Reproduction* 2015 **30** 2031-2037.

77. Mastrogiacomo I, Motta RG, Botteon S, Bonanni G & Schiesaro M. Achievement of spermatogenesis and genital tract maturation in hypogonadotropic hypogonadic subjects during long term treatment with gonadotropins or LHRH. *Andrologia* 1991 **23** 285-289.

78. Matsumoto AM, Snyder PJ, Bhasin S, Martin K, Weber T, Winters S, Spratt D, Brentzel J & O'Dea L. Stimulation of spermatogenesis with recombinant human follicle-stimulating hormone (follitropin alfa; GONAL-f): long-term treatment in azoospermic men with hypogonadotropic hypogonadism. *Fertility and Sterility* 2009 **92** 979-990.

79. McLachlan RI, Finkel DM, Bremner WJ & Snyder PJ. Serum inhibin concentrations before and during gonadotropin treatment in men with hypogonadotropic hypogonadism: Physiological and clinical implications. *Journal of Clinical Endocrinology and Metabolism* 1990 **70** 1414-1419.

80. Miyagawa Y, Tsujimura A, Matsumiya K, Takao T, Tohda A, Koga M, Takeyama M, Fujioka H, Takada S, Koide T & Okuyama A. Outcome of gonadotropin therapy for male hypogonadotropic hypogonadism at university affiliated male infertility centers: A 30-year retrospective study. *Journal of Urology* 2005 **173** 2072-2075.

81. Morris GC, Lloyd-Evans E & Cahill DJ. Induction of spermatogenesis in men with hypogonadotropic hypogonadism. *Journal of Assisted Reproduction and Genetics* 2021 **38** 803-807.

82. Nieschlag E, Bouloux PMG, Stegmann BJ, Shankar RR, Guan Y, Tzontcheva A, McCrary Sisk C & Behre HM. An open-label clinical trial to investigate the efficacy and safety of corifollitropin alfa combined with hCG in adult men with hypogonadotropic hypogonadism. *Reproductive Biology and Endocrinology* 2017 **15** 17.

83. Oguz A, Tuzun D, Sahin M, Bulbul N, Celik A, Guvenc N, Inanc E & Gul K. Should human chorionic gonadotropine treatment increase thyroid volume? *Archives of endocrinology and metabolism* 2015 **59** 482-486.

84. Okada Y, Kondo T, Okamoto S & Ogawa M. Induction of Ovulation and Spermatogenesis by hMG/hCG in Hypogonadotropic GH-Deficient Patients. *Endocrinologia Japonica* 1992 **39** 31-43.

85. Oldereid NB, Byholm T & Tanbo TG. Spermatogenesis and fertility outcome in male hypogonadotrophic hypogonadism. *Human Fertility* 2010 **13** 83-89.

86. Raivio T, Wikstrom AM & Dunkel L. Treatment of gonadotropin-deficient boys with recombinant human FSH: Long-term observation and outcome. *European Journal of Endocrinology* 2007 **156** 105-111.

87. Rehman KU, Shahid K & Humayun H. Hypogonadotropic hypogonadism: New identification of testicular blood flow and varicocele after treatment with gonadotropins. *Fertility and Sterility* 2014 **102** 700-704.e701.

88. Resorlu B, Abdulmajed MI, Kara C, Unsal A & Aydos K. Is intracytoplasmic sperm injection essential for the treatment of hypogonadotrophic hypogonadism? A comparison between idiopathic and secondary hypogonadotrophic hypogonadism. *Human Fertility* 2009 **12** 204-208.

89. Rohayem J, Sinthofen N, Nieschlag E, Kliesch S & Zitzmann M. Causes of hypogonadotropic hypogonadism predict response to gonadotropin substitution in adults. *Andrology* 2016 **4** 87-94.

90. Rohayem J, Hauffa BP, Zacharin M, Kliesch S, Zitzmann M, Wusthof A, Tittel B, Morlot M, Vorhoff W, Muller-Rossberg E, Wagner V, Konrad K, Salzgeber K, Fricke-Otto S, Wolfle J, Willig P, Karges B, Lankes E, Droste M, Richter-Unruh A, Liesenkotter KP, Hubner A, Zichner S, Welzel M, Dunstheimer D, Potelajew U, Heger S, Hammer E, Oevering R, Schmidt S & Nieschlag E. Testicular growth and spermatogenesis: new goals for pubertal hormone replacement in boys with hypogonadotropic hypogonadism? -a multicentre prospective study of hCG/rFSH treatment outcomes during adolescence. *Clinical Endocrinology* 2017 **86** 75-87.

91. Sato N, Hasegawa T, Hasegawa Y, Arisaka O, Ozono K, Amemiya S, Kikuchi T, Tanaka H, Harada S, Miyata I & Tanaka T. Treatment situation of male hypogonadotropic hypogonadism in pediatrics and proposal of testosterone and gonadotropins replacement therapy protocols. *Clinical Pediatric Endocrinology* 2015 **24** 37-49.

92. Shankar RR, Shah S, Joeng HK, Mendizabal G, Dibello JR, Guan Y, Stegmann BJ, Nieschlag E, Behre HM, Swerdloff RS, Fox MC & Kaufman KD. Corifollitropin Alfa Combined With Human Chorionic Gonadotropin in Adolescent Boys With Hypogonadotropic Hypogonadism. *Journal of Clinical Endocrinology and Metabolism* 2022 **107** 2036-2046.

93. Shiraishi K, Oka S & Matsuyama H. Assessment of quality of life during gonadotrophin treatment for male hypogonadotrophic hypogonadism. *Clinical Endocrinology* 2014 **81** 259-265.

94. Shiraishi K, Ohmi C & Matsuyama H. Patient-reported outcomes and biochemical alterations during hormonal therapy in men with hypogonadotropic hypogonadism who have finished infertility treatment. *Endocrine Journal* 2021 **68** 221-229.

95. Sinisi AA, Esposito D, Maione L, Quinto MC, Visconti D, De Bellis A, Bellastella A, Conzo G & Bellastella G. Seminal anti-Mullerian hormone level is a marker of spermatogenic response during long-term gonadotropin therapy in male hypogonadotropic hypogonadism. *Human Reproduction* 2008 **23** 1029-1034.

96. Sinisi AA, Esposito D, Bellastella G, Maione L, Palumbo V, Gandini L, Lombardo F, De Bellis A, Lenzi A & Bellastella A. Efficacy of recombinant human follicle stimulating hormone at low doses in inducing spermatogenesis and fertility in hypogonadotropic hypogonadism. *Journal of Endocrinological Investigation* 2010 **33** 618-623.

97. Trinh TS, Hung NB, Hien LTT, Tuan NA, Pho DC, Dung QA, Do DA, Quang HD, Van Ai H & Hung PN. Evaluating the combination of human chorionic gonadotropin and clomiphene citrate in treatment of male hypogonadotropic hypogonadism: A prospective study. *Research and Reports in Urology* 2021 **13** 357-366.

98. Unluhizarci K, Sik SK, Keti DB, Kose K, Hacioglu A & Karaca Z. Treatment of male hypogonadism partially reverses oxidative stress in patients with hypogonadism. *Endocrine Journal* 2020 **67** 935-940.

99. Wang Q, Jiang W, Li G, Tang L & Hu Y. Comparison of therapeutic response to gonadotropin therapy between chinese male adolescents and young adults with hypogonadotropic hypogonadism caused by pituitary stalk interruption. *Hormone and Metabolic Research* 2014 **46** 668-673.

100. Warne DW, Decosterd G, Okada H, Yano Y, Koide N & Howles CM. A combined analysis of data to identify predictive factors for spermatogenesis in men with hypogonadotropic hypogonadism treated with recombinant human follicle-stimulating hormone and human chorionic gonadotropin. *Fertility and Sterility* 2009 **92** 594-604.

101. Yang L, Zhang SX, Dong Q, Xiong ZB & Li X. Application of hormonal treatment in hypogonadotropic hypogonadism: More than ten years experience. *International Urology and Nephrology* 2012 **44** 393-399.

102. Young J, Rey R, Couzinet B, Chanson P, Josso N & Schaison G. Antimullerian hormone in patients with hypogonadotropic hypogonadism. *Journal of Clinical Endocrinology and Metabolism* 1999 **84** 2696-2699.

103. Zacharin M, Sabin MA, Nair VV & Dagabdhao P. Addition of recombinant follicle-stimulating hormone to human chorionic gonadotropin treatment in adolescents and young adults with hypogonadotropic hypogonadism promotes normal testicular growth and may promote early spermatogenesis. *Fertility and Sterility* 2012 **98** 836-842.

**
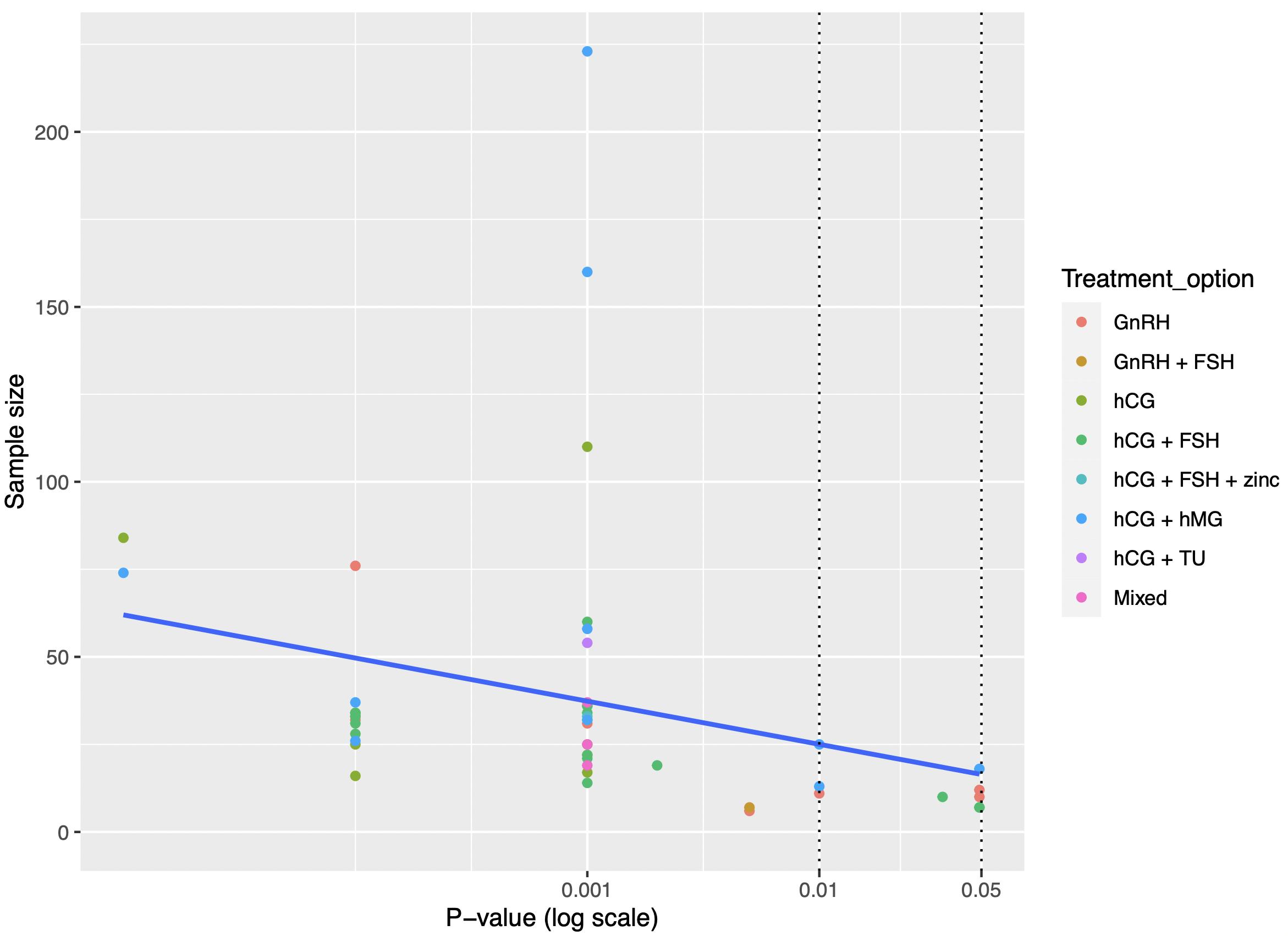
**

**Supplementary Figure 1**: **Scatter plot of p-values for significance of the increase in testicular volume observed post-treatment with gonadotropin therapy**

**[GnRH = Gonadotropin-releasing hormone; hCG = Human chorionic gonadotropin; hMG = Human menopausal gonadotropins; FSH = Follicle-stimulating hormone; TU = Testosterone undecanoate]** Studies listing p<0.05 were approximated to p=0.049. Four studies which did not provide p-values (three stating a significant increase, one no significant change) were not plotted.

**
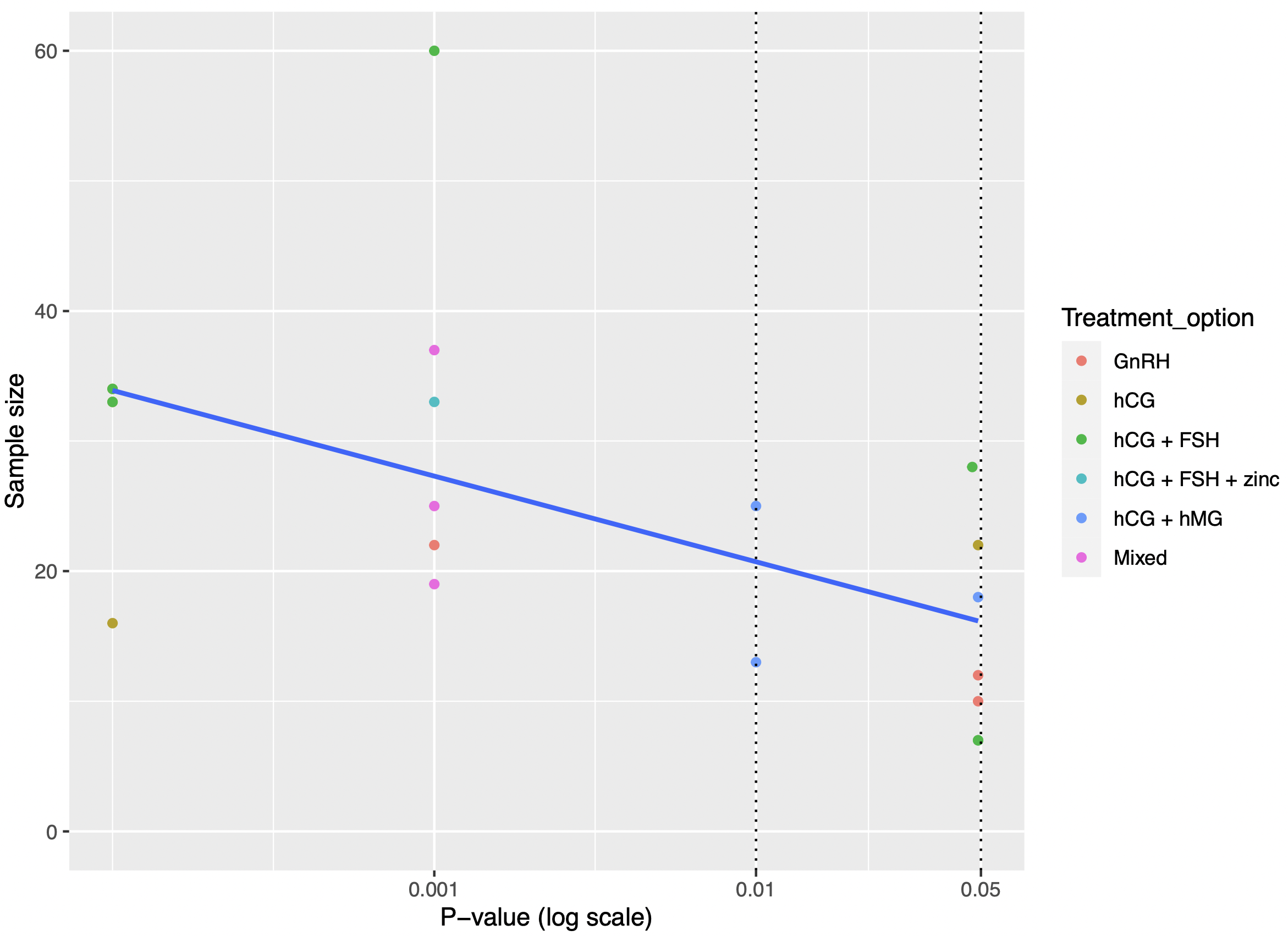
**

**Supplementary Figure 2: Scatter plot of p-values for significance of the increase penile length observed post-treatment with gonadotropin therapy [GnRH = Gonadotropin-releasing hormone; hCG = Human chorionic gonadotropin; hMG = Human menopausal gonadotropins; FSH = Follicle-stimulating hormone]**


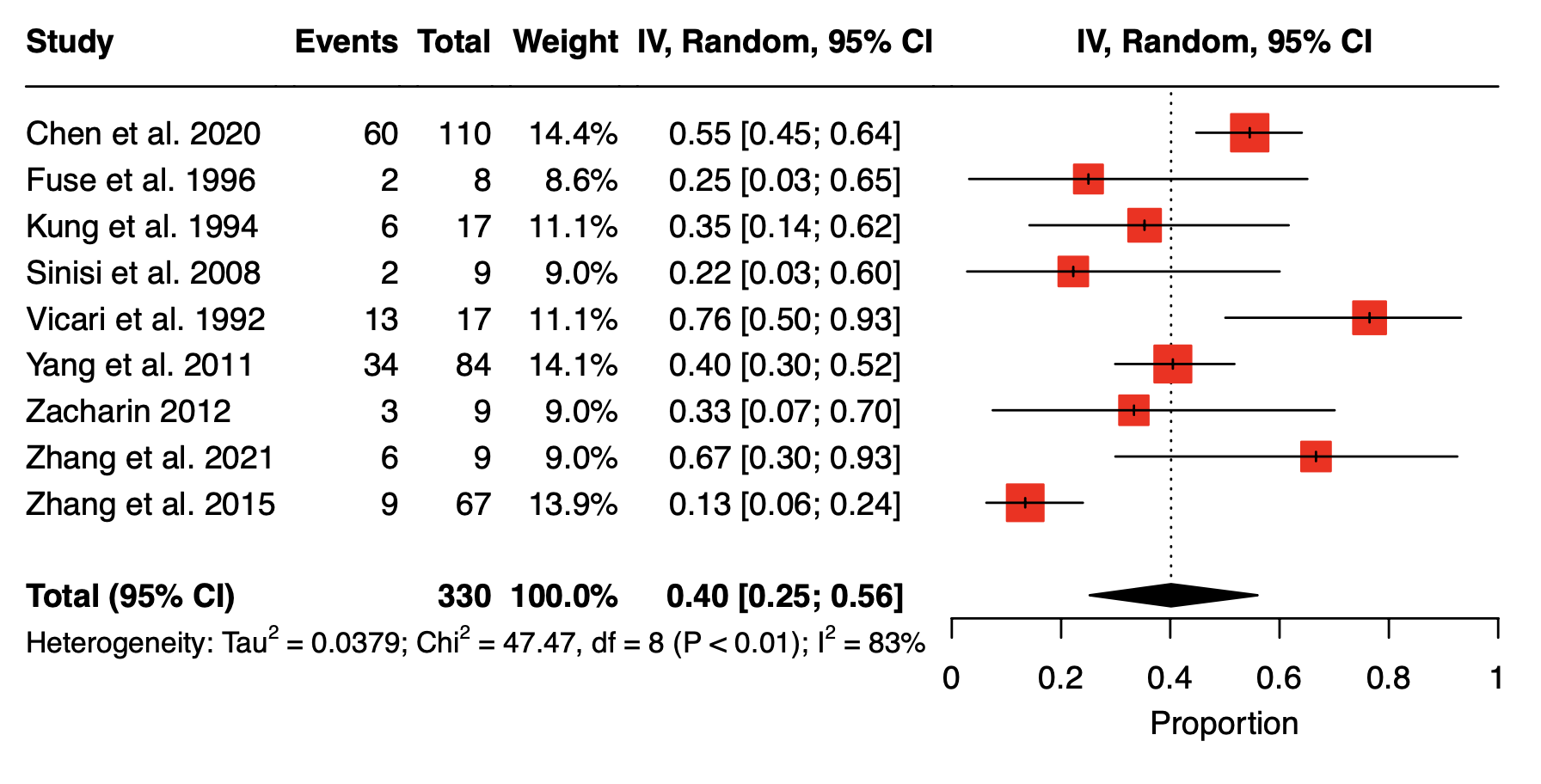


**Supplementary Figure 3:**  **Meta-analysis of proportions for spermatogenesis achieved by hCG in patients with hypogonadotropic hypogonadism – pooled proportion (random effect) 0.40 (95% CI 0.25-0.56)**

**
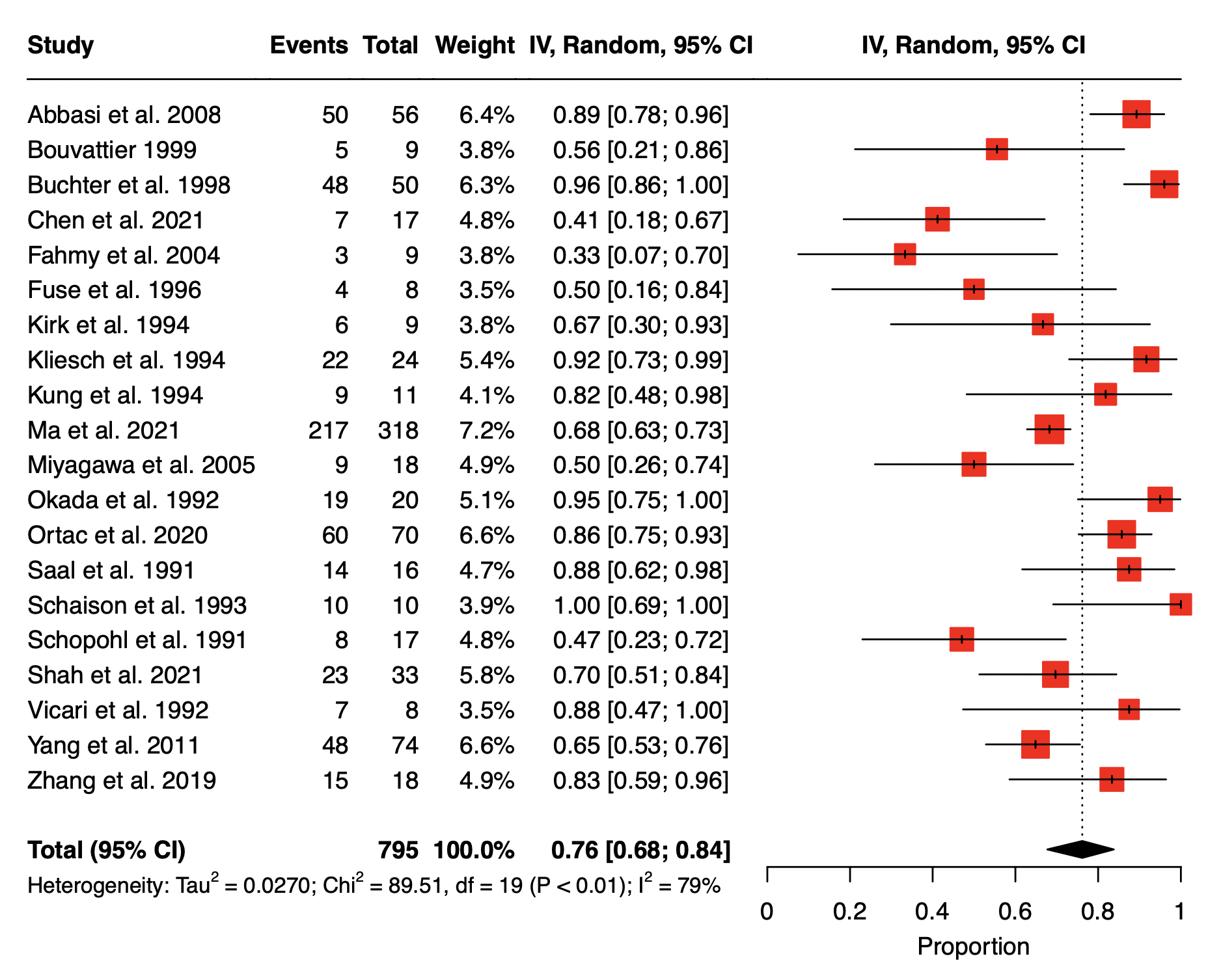
**

**Supplementary Figure 4: Meta-analysis of proportions for spermatogenesis achieved by hCG + hMG in patients with hypogonadotropic hypogonadism – pooled proportion (random effect) 0.76 (95% CI 0.68-0.84)**


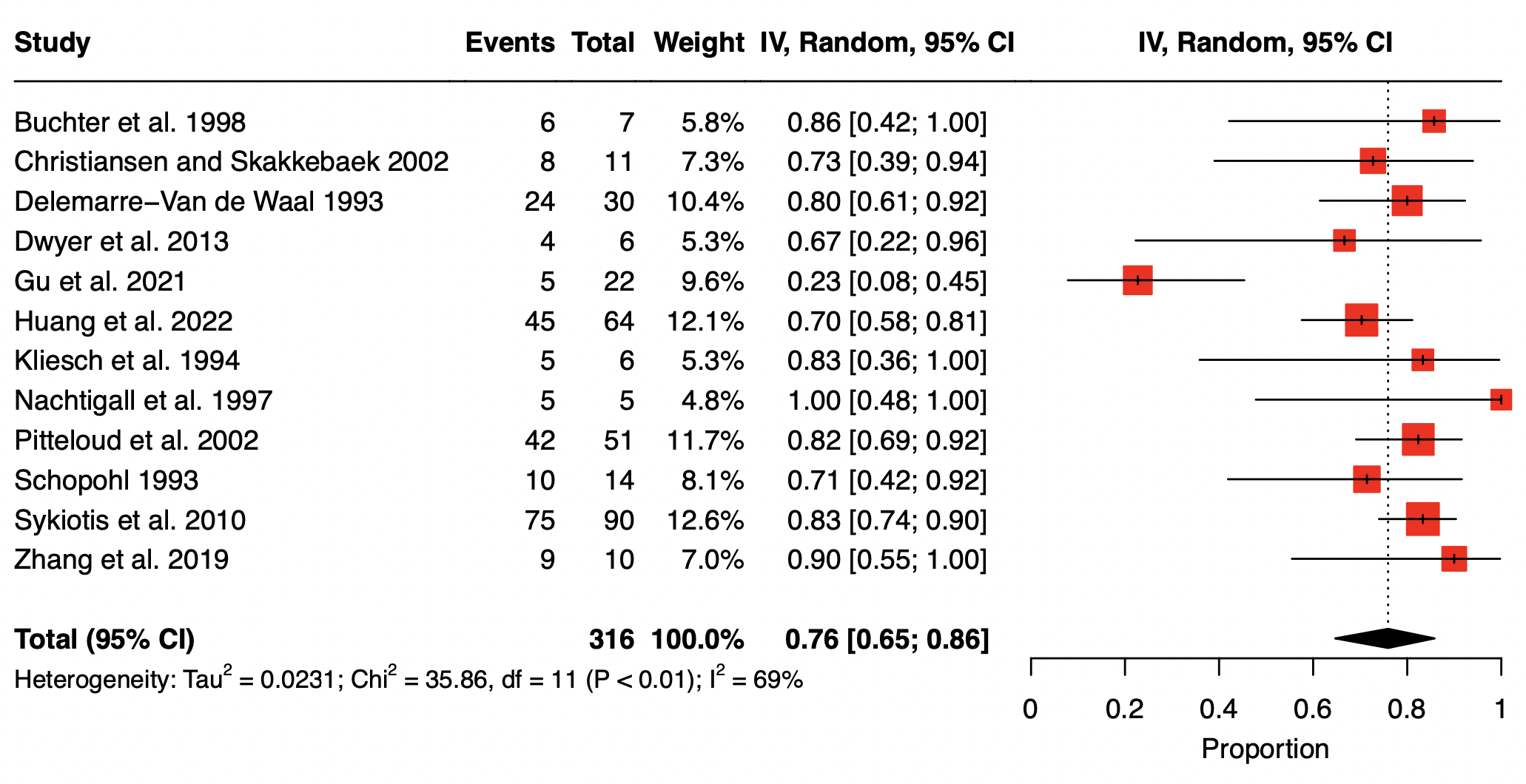


**Supplementary Figure 5: Meta-analysis of proportions for spermatogenesis achieved by GnRH in patients with hypogonadotropic hypogonadism – pooled proportion (random effect) 0.76 (95% CI 0.65-0.86)**

**
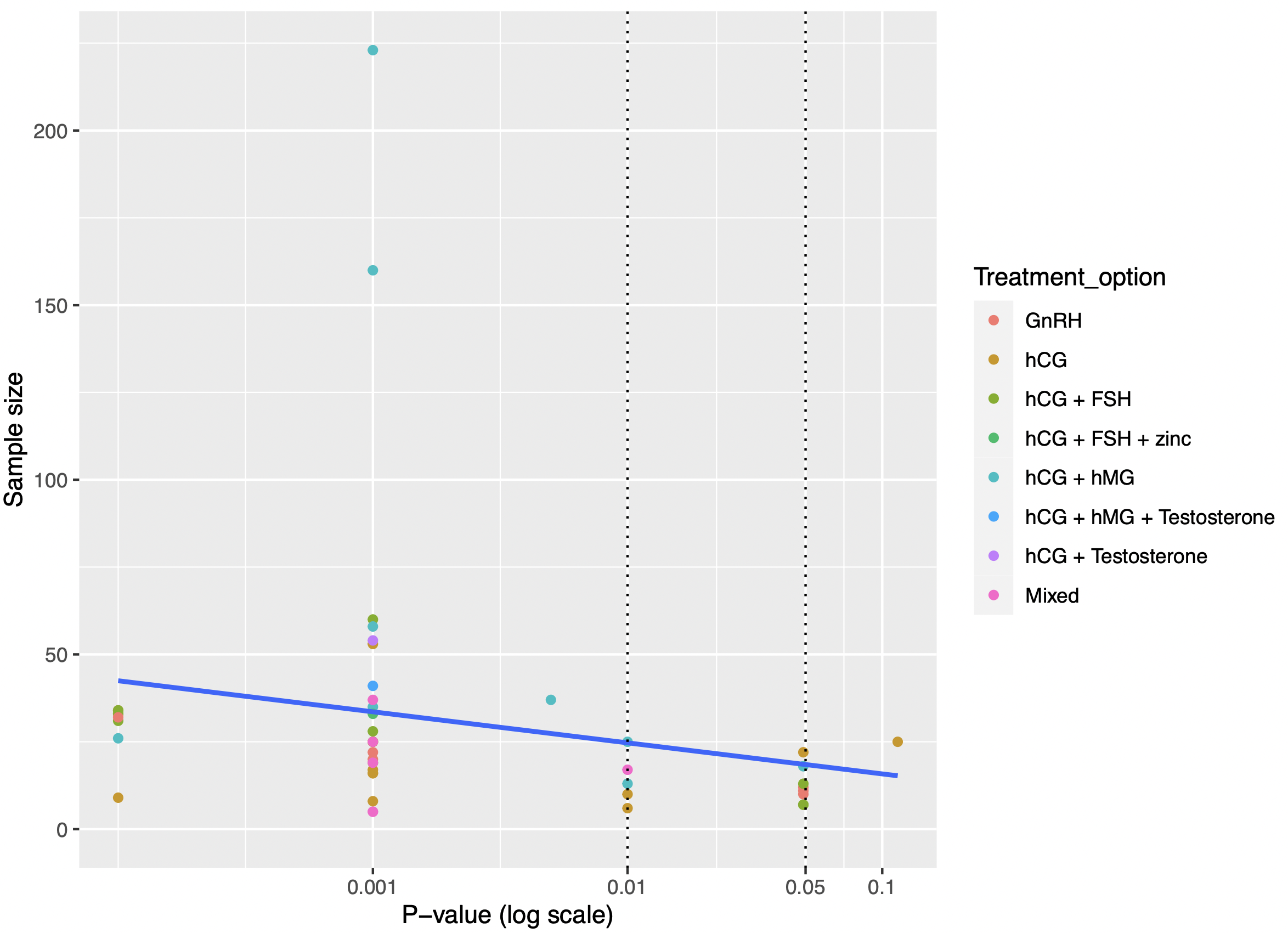
**

**Supplementary Figure 6: Scatter plot of p-values for significance of the increase in testosterone concentration observed post-treatment with gonadotropin therapy [T = testosterone; GnRH = Gonadotropin-releasing hormone; hCG = Human chorionic gonadotropin; hMG = Human menopausal gonadotropins; FSH = Follicle-stimulating hormone; TU = Testosterone undecanoate]** Two cohorts which did not list p-values were not plottable.

**
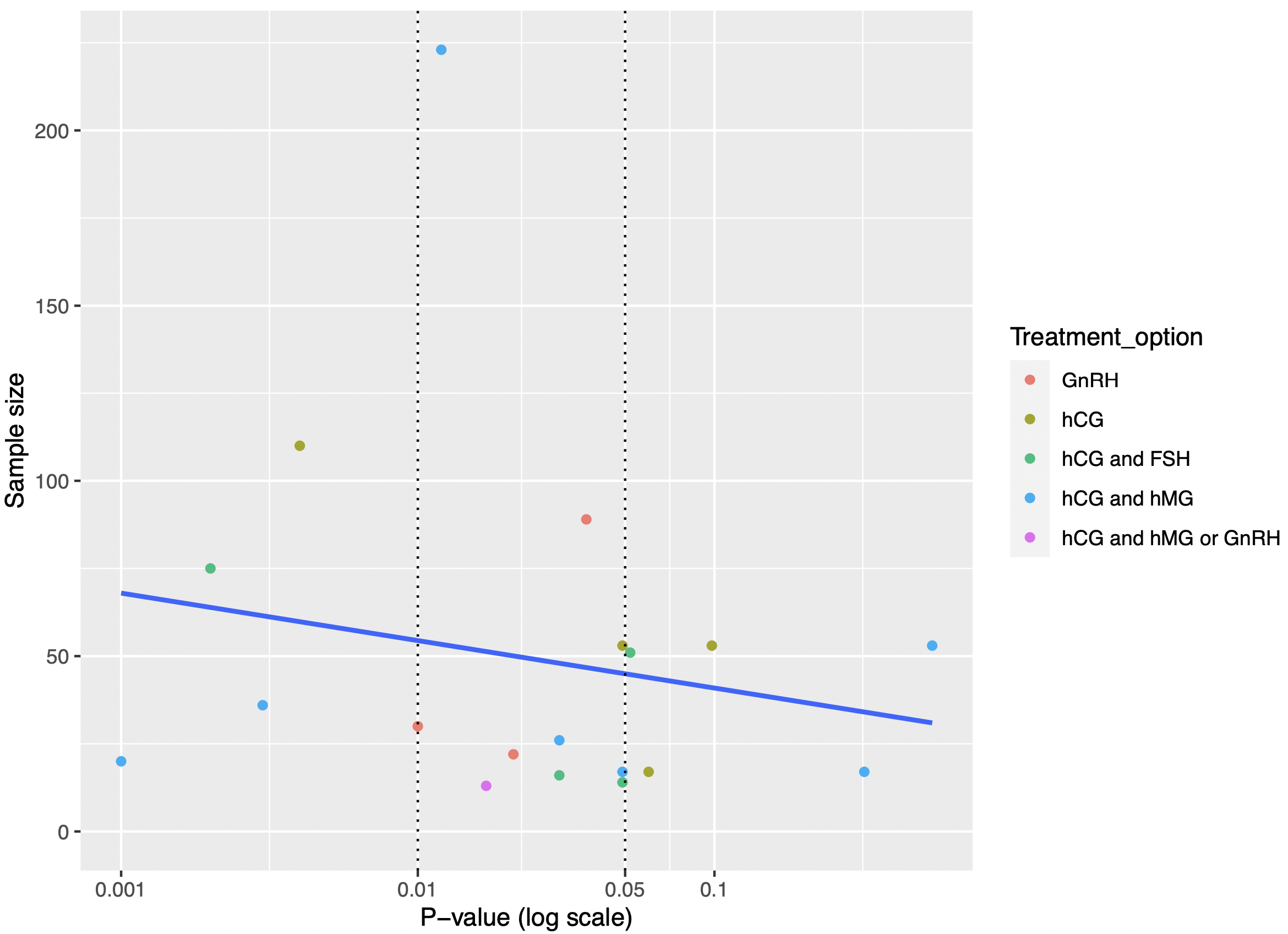
**

**Supplementary Figure 7: Scatter plot of relationship between sample size and impact of initial testicular volume on spermatogenesis markers (pooled). It was not possible to plot studies which did not provide p-values in the main text, of which seven described no significant impact of initial testicular volume on components of spermatogenesis (with a median sample size 18) ^9, 11, 19, 50, 56, 63, 70^, and two which found a significant impact (samples of 13 and 100 subjects) ^85, 100^.**


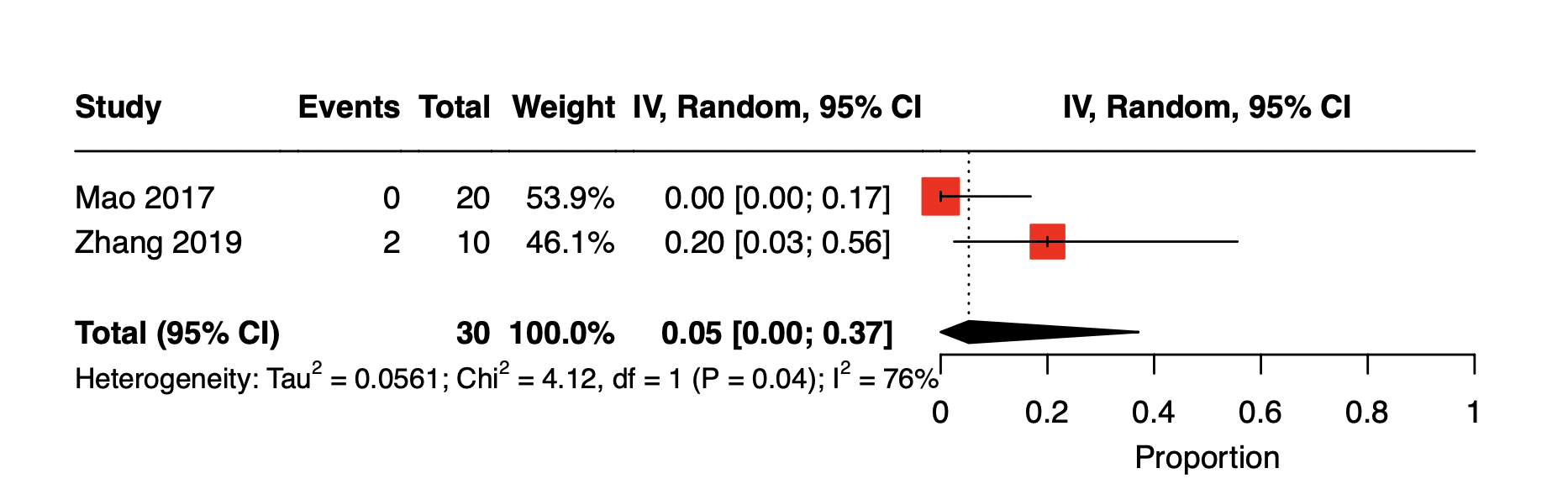


**Supplementary Figure 8: Meta-analysis of proportions of patients developing acne when treated with GnRH**


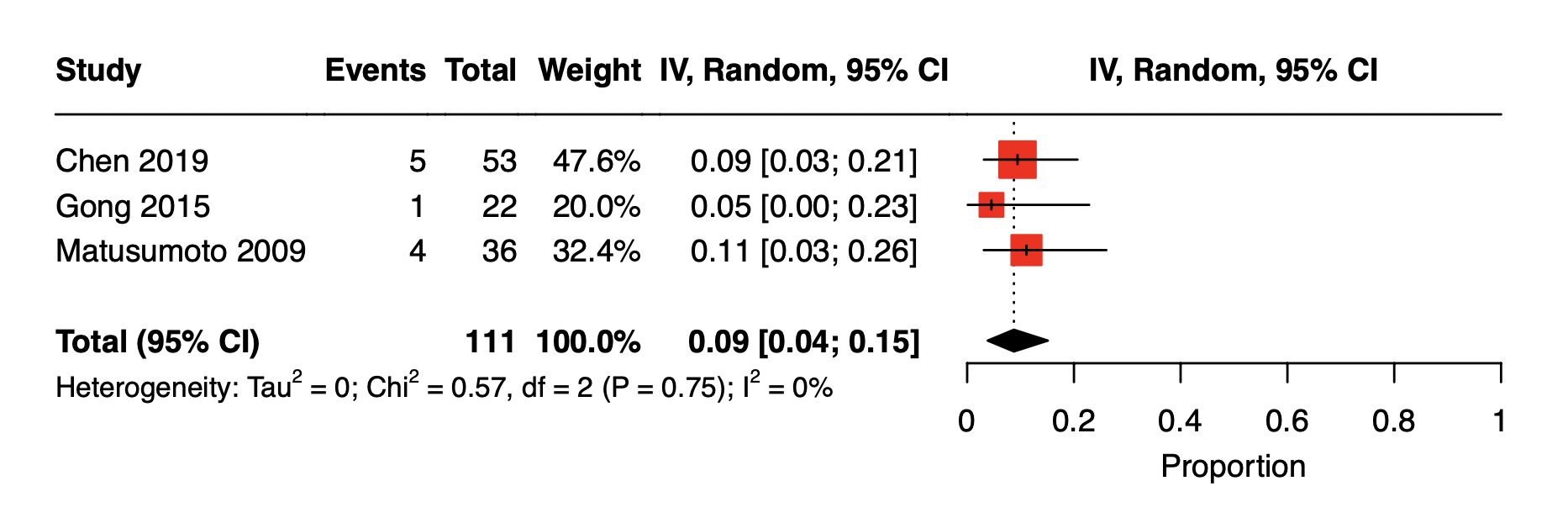


**Supplementary Figure 9: Meta-analysis of proportions of patients developing acne when treated with hCG**


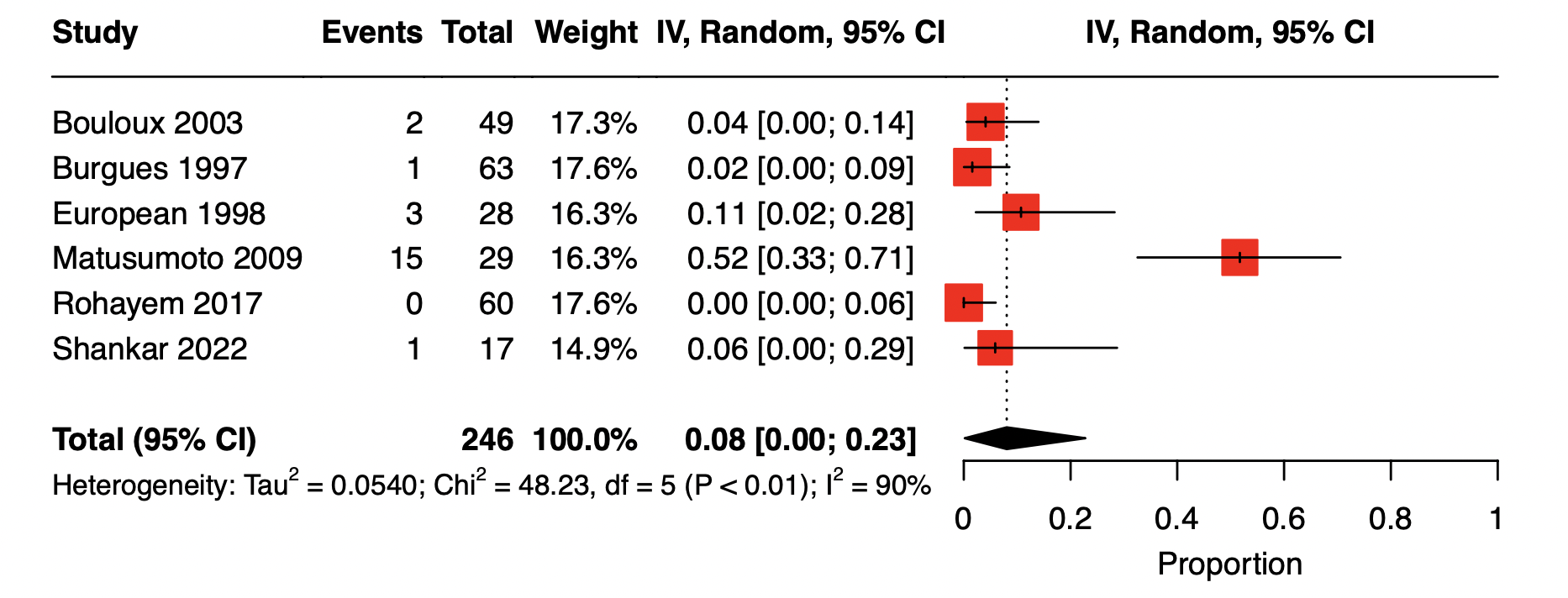


**Supplementary Figure 10: Meta-analysis of proportions of patients developing acne when treated with hCG + FSH**

**
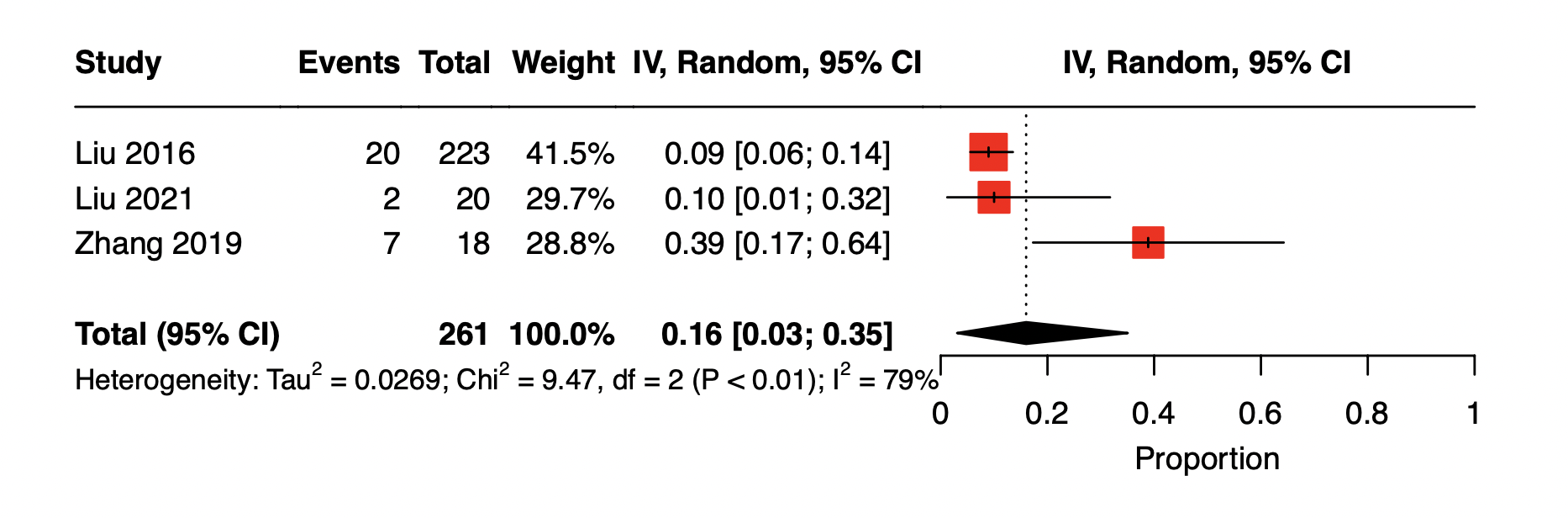
**

**Supplementary Figure 11: Meta-analysis of proportions of patients developing acne when treated with hCG + hMG**

**
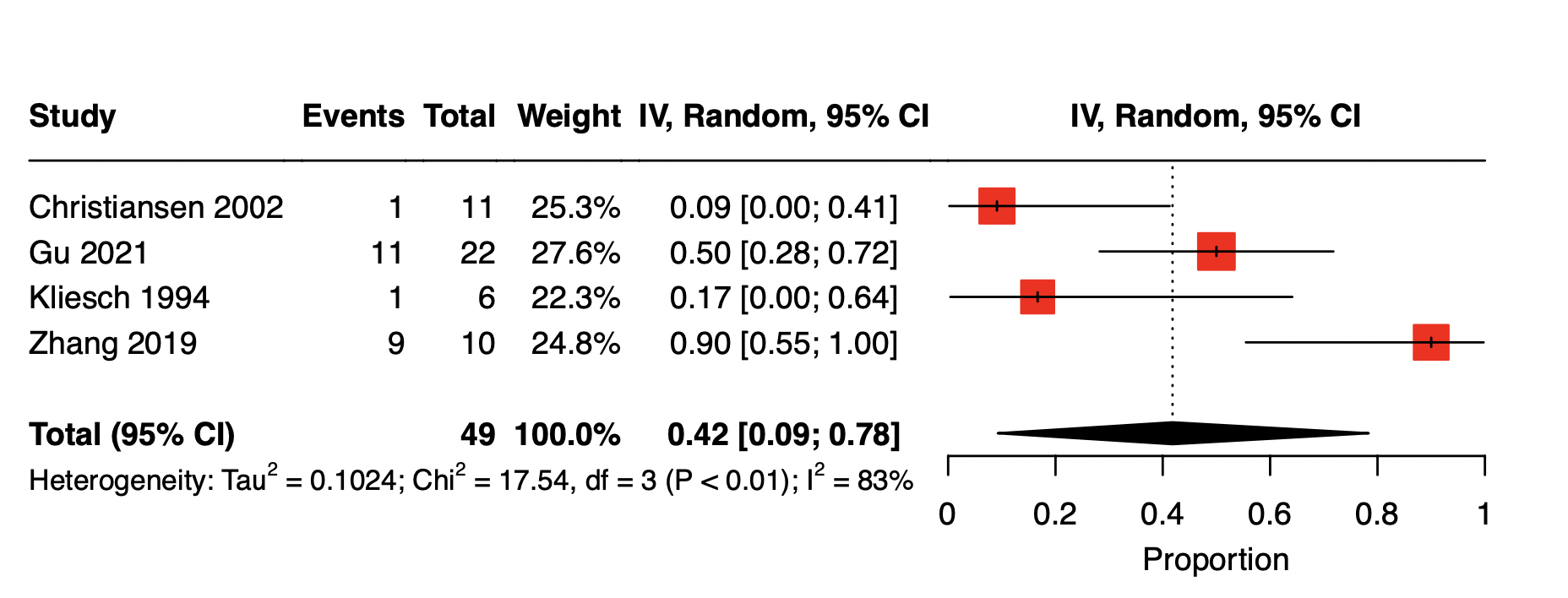
**

**Supplementary Figure 12: Meta-analysis of proportions of patients developing injection site reaction/pain when treated with GnRH**

**
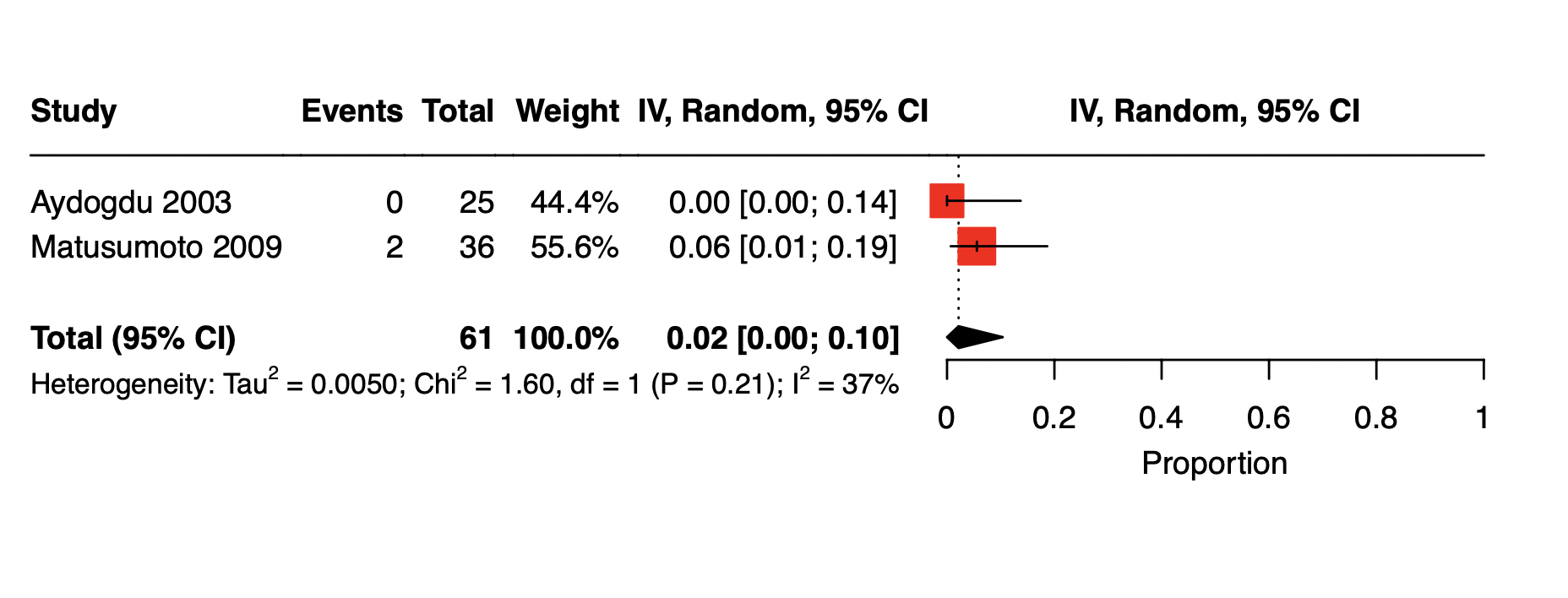
**

**Supplementary Figure 13: Meta-analysis of proportions of patients developing injection site reaction/pain when treated with hCG**

**
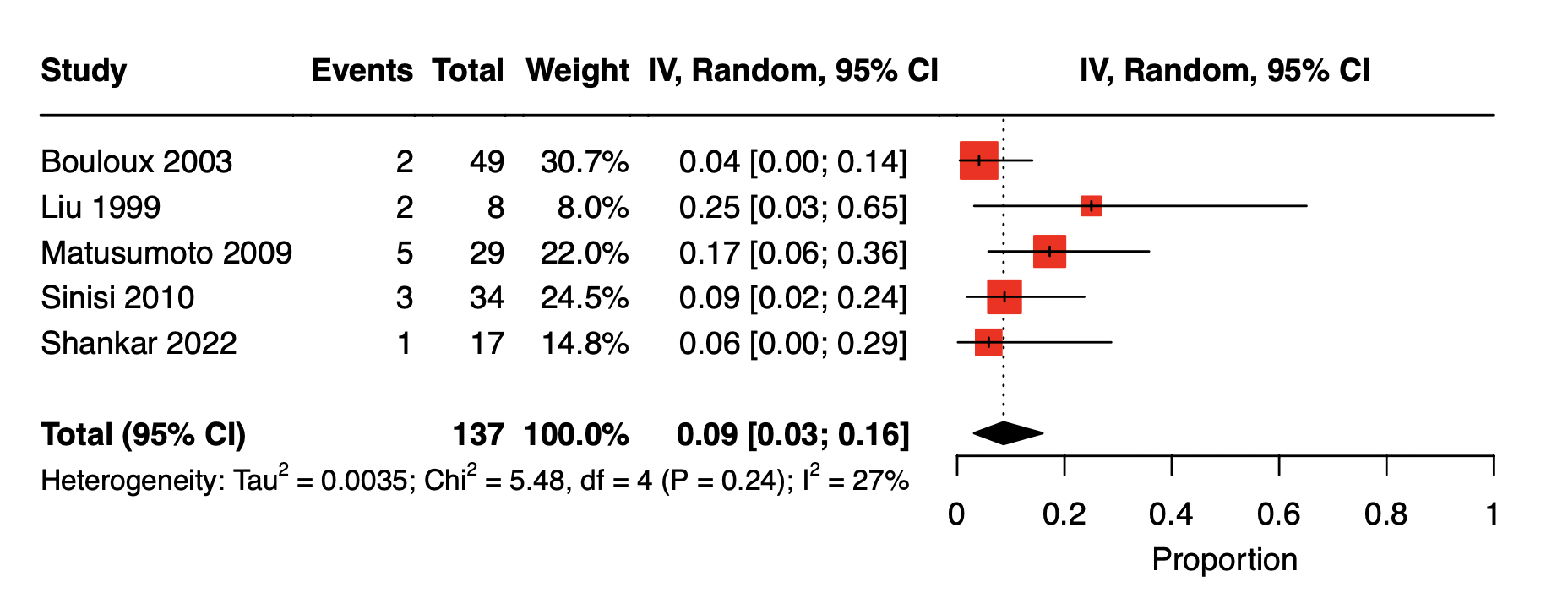
**

**Supplementary Figure 14: Meta-analysis of proportions of patients developing injection site reaction/pain when treated with hCG + FSH**

**
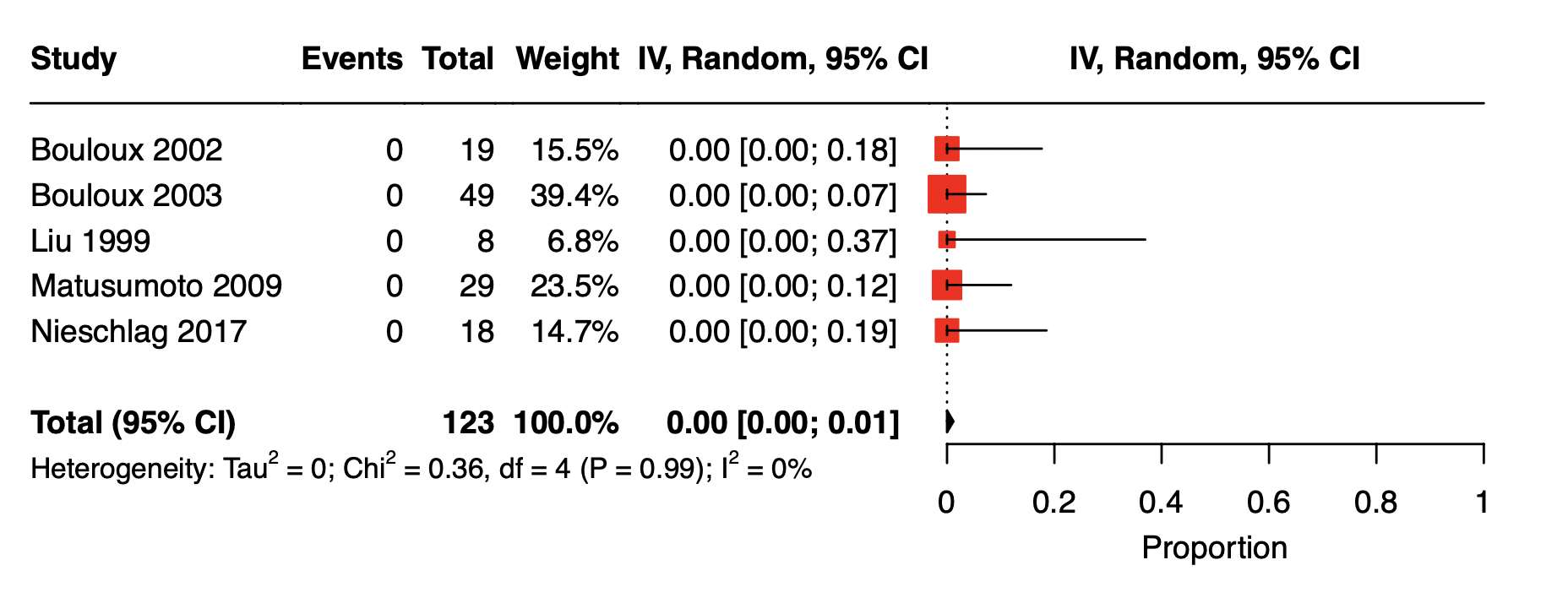
**

**Supplementary Figure 15: Meta-analysis of proportions of patients developing anti-FSH antibodies when treated with hCG + FSH**

**
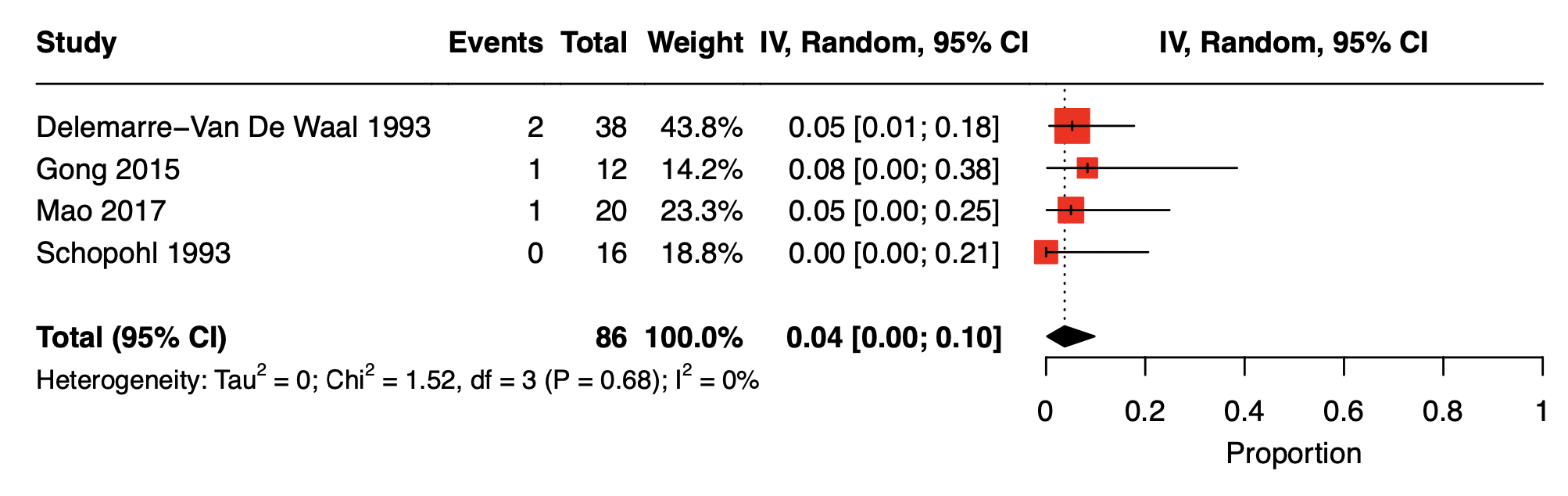
**

**Supplementary Figure 16: Meta-analysis of proportions of patients developing gynaecomastia when treated with GnRH**


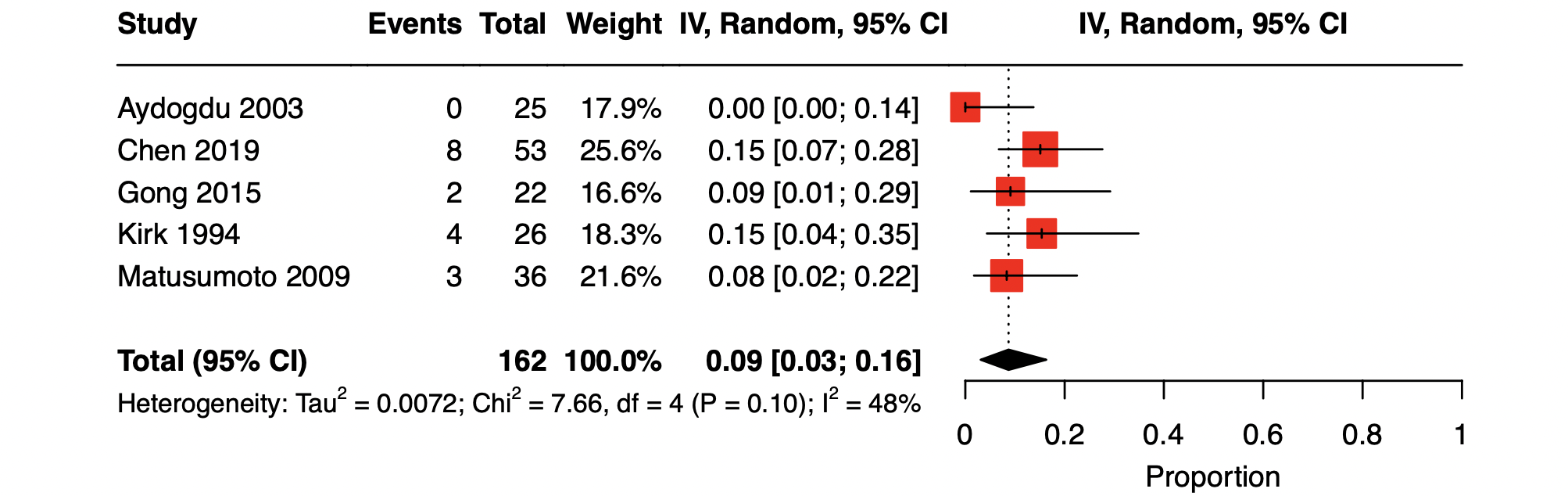


**Supplementary Figure 17: Meta-analysis of proportions of patients developing gynaecomastia when treated with hCG**

**
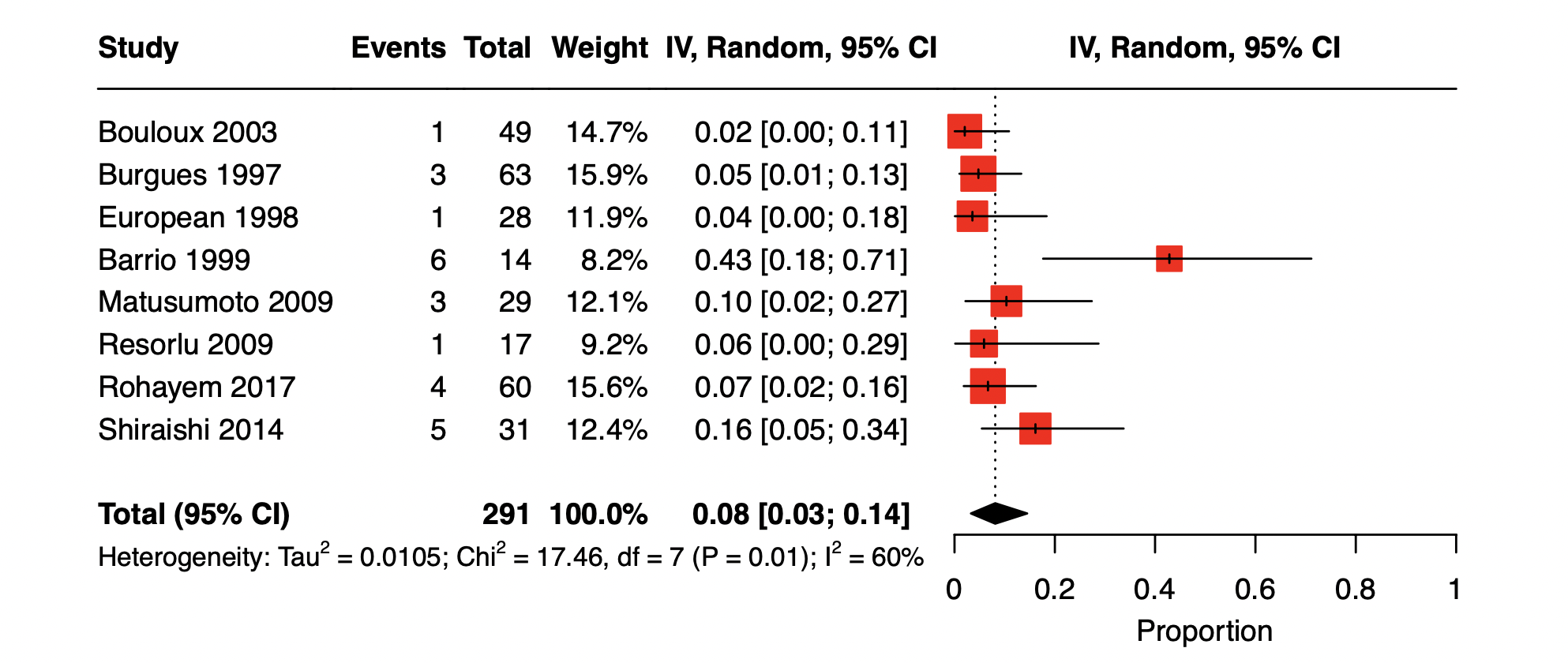
**

**Supplementary Figure 18: Meta-analysis of proportions of patients developing gynaecomastia when treated with hCG + FSH**

**
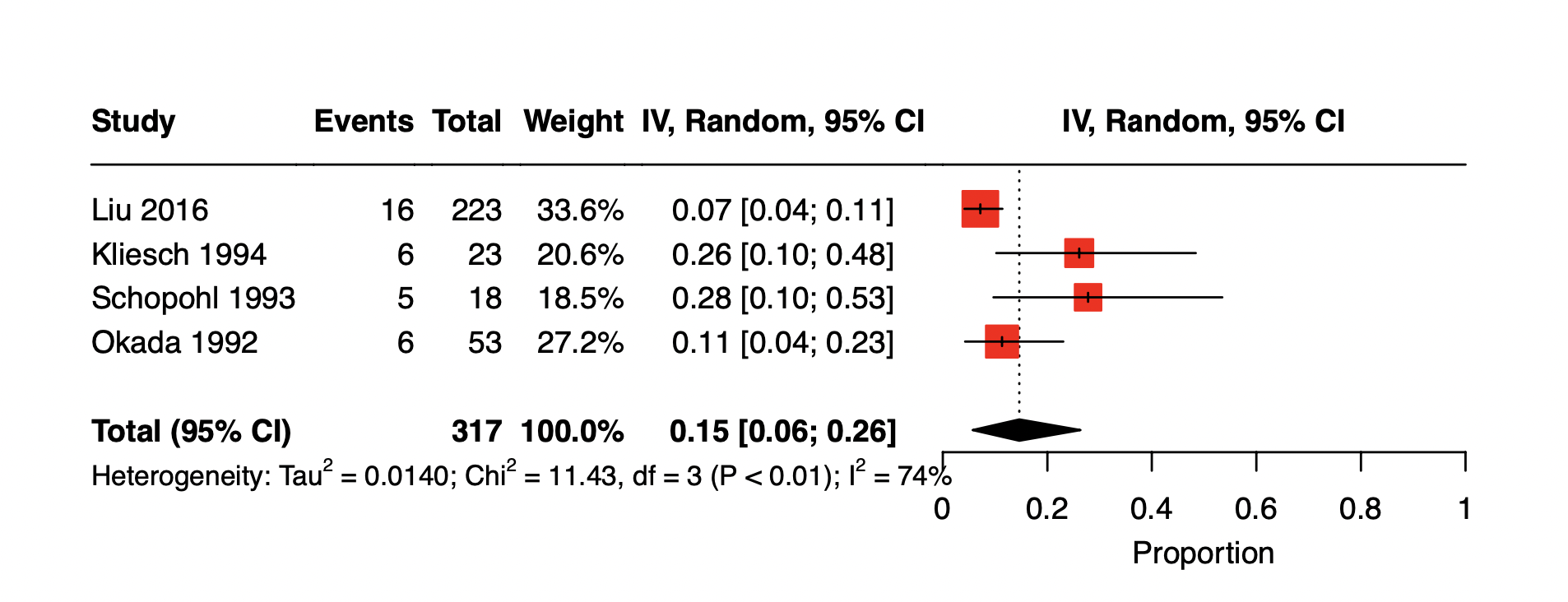
**

**Supplementary Figure 19: Meta-analysis of proportions of patients developing gynaecomastia when treated with hCG + hMG**
